# Supplementary material for: Antibiotic-chemoattractants enhance neutrophil clearance of Staphylococcus aureus
Source: Nat Commun. 2021 Oct 25;12:6157. doi: 10.1038/s41467-021-26244-5 (PMC8546149; doi:10.1038/s41467-021-26244-5)
Supplement: Supplementary file 1 — Supplementary Information [file 41467_2021_26244_MOESM1_ESM.pdf]

SUPPLEMENTARY INFORMATION

SUPPLEMENTARY FIGURES

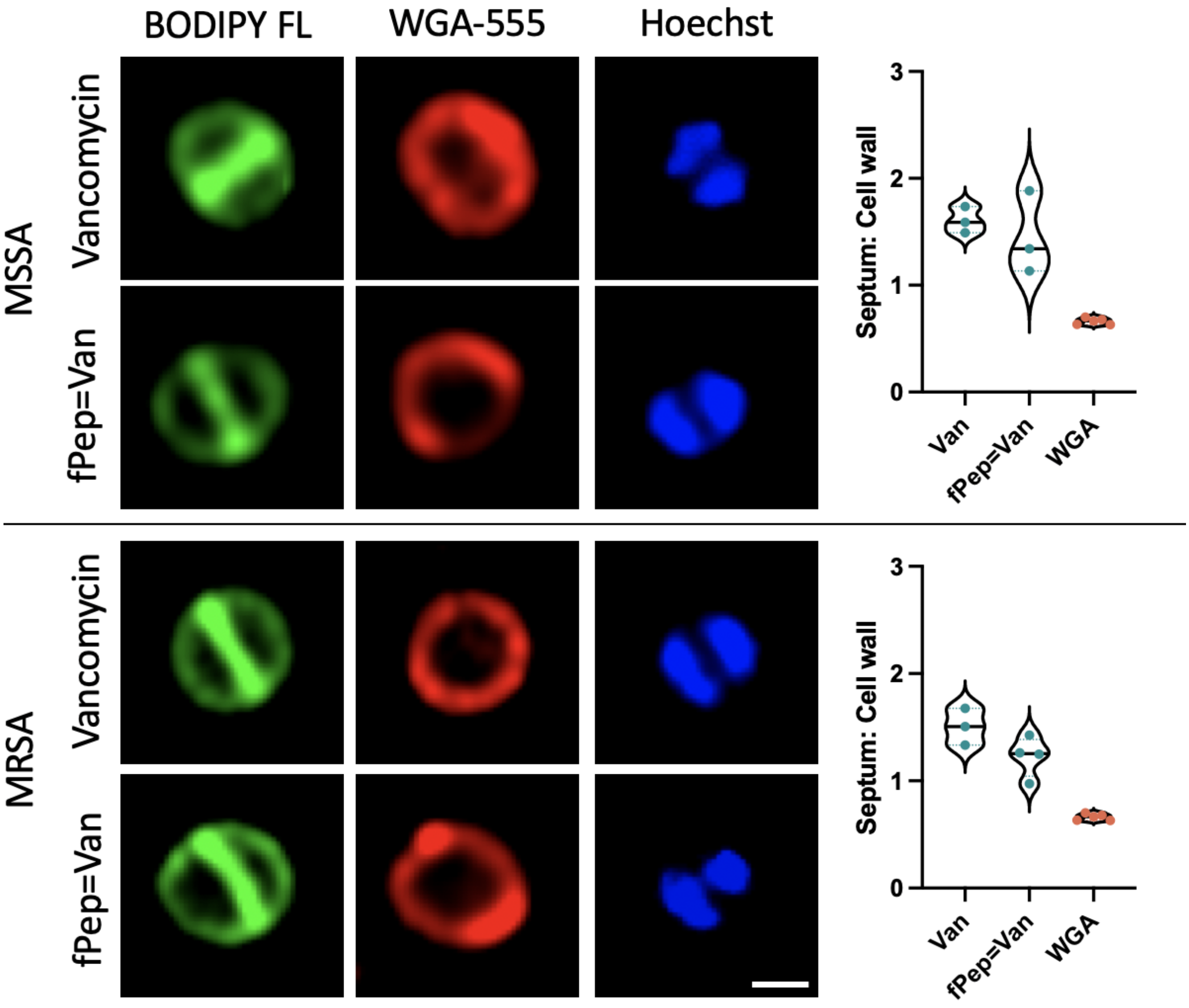

Supplementary Figure 1. Distribution of vancomycin binding to *S. aureus*
Binding of BODIPY labelled vancomycin (van, **B2**) or fMLFG conjugated to vancomycin (fPep=van, **B3**) to *S. aureus* strains (MSSA, ATCC strain; MRSA, A8090). The average ratio in fluorescence intensity was calculated at the septum vs the wall of the *S. aureus* cell from Airyscan microscope images of at least 120 cells per condition across at least three biological replicates and plotted with median and quartiles as violin plots. The median ratio of 1.5 was observed for vancomycin, and 1.3 for fPep=van binding to MRSA and MSSA. This indicates higher intensity of fluorescence at the septum compared to the wall. In contrast, Wheat germ agglutin staining (WGA-555nm), with a ratio of 0.6 had greater binding to the cell wall. Scale bar shown is 0.5 microns.

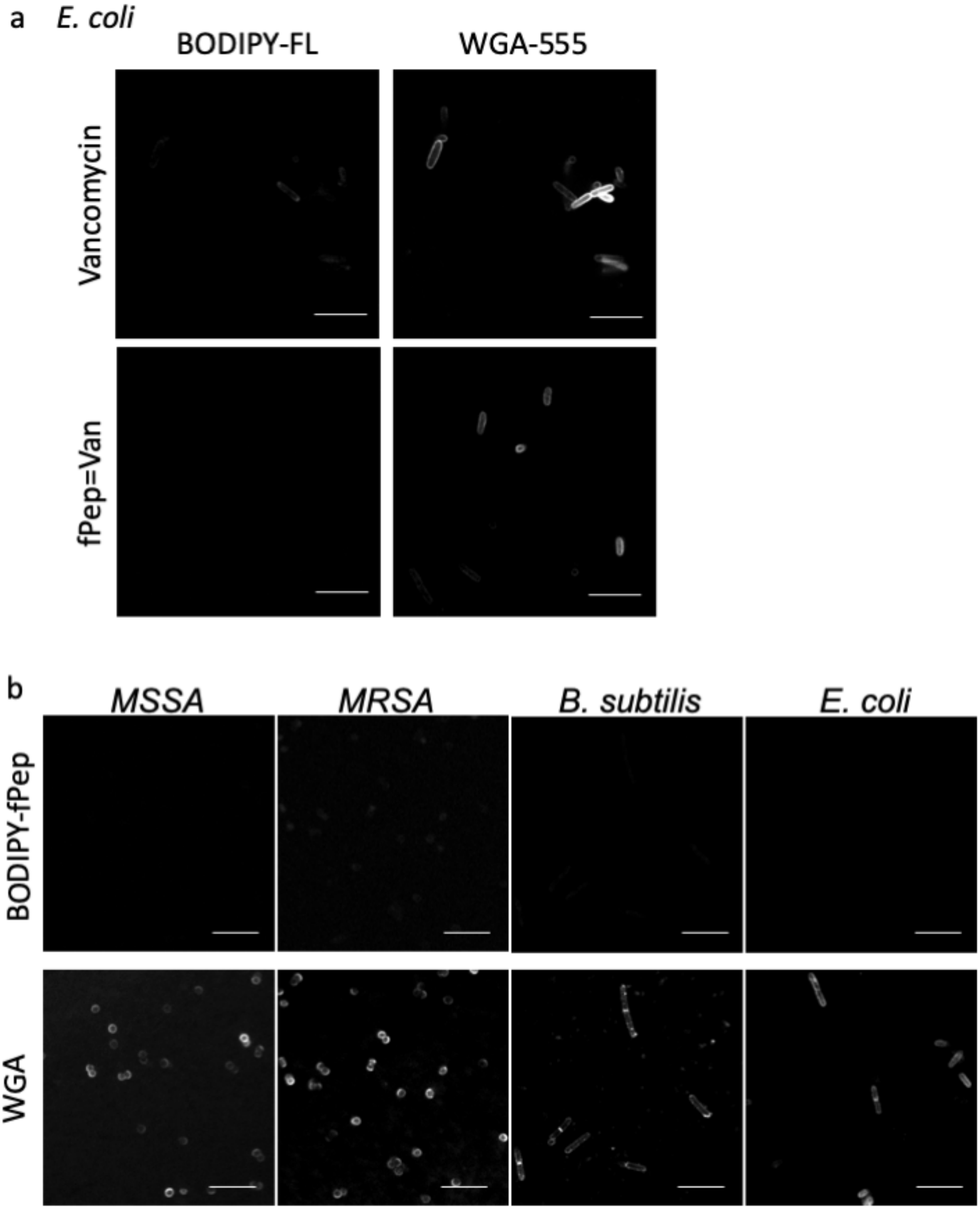

Supplementary Figure 2. Qualifying vancomycin and fPep binding to various bacterial strains

(a) Analysis of the binding of BODIPY labelled vancomycin (van, **B2**) or fPep conjugated to vancomycin (fPep=van, **B3**) to the Gram-negative bacteria *E. coli*. (b) The BODIPY labelled fPep (**B1**) alone interaction with bacterial strains. Fluorescent intensity was set relative to fluorescence observed for binding to Gram-positive strains. Scale bars are 3 micron and cells were counterstained with wheat germ agglutinin 555nm (WGA). Images are representative of 3 biological replicates.

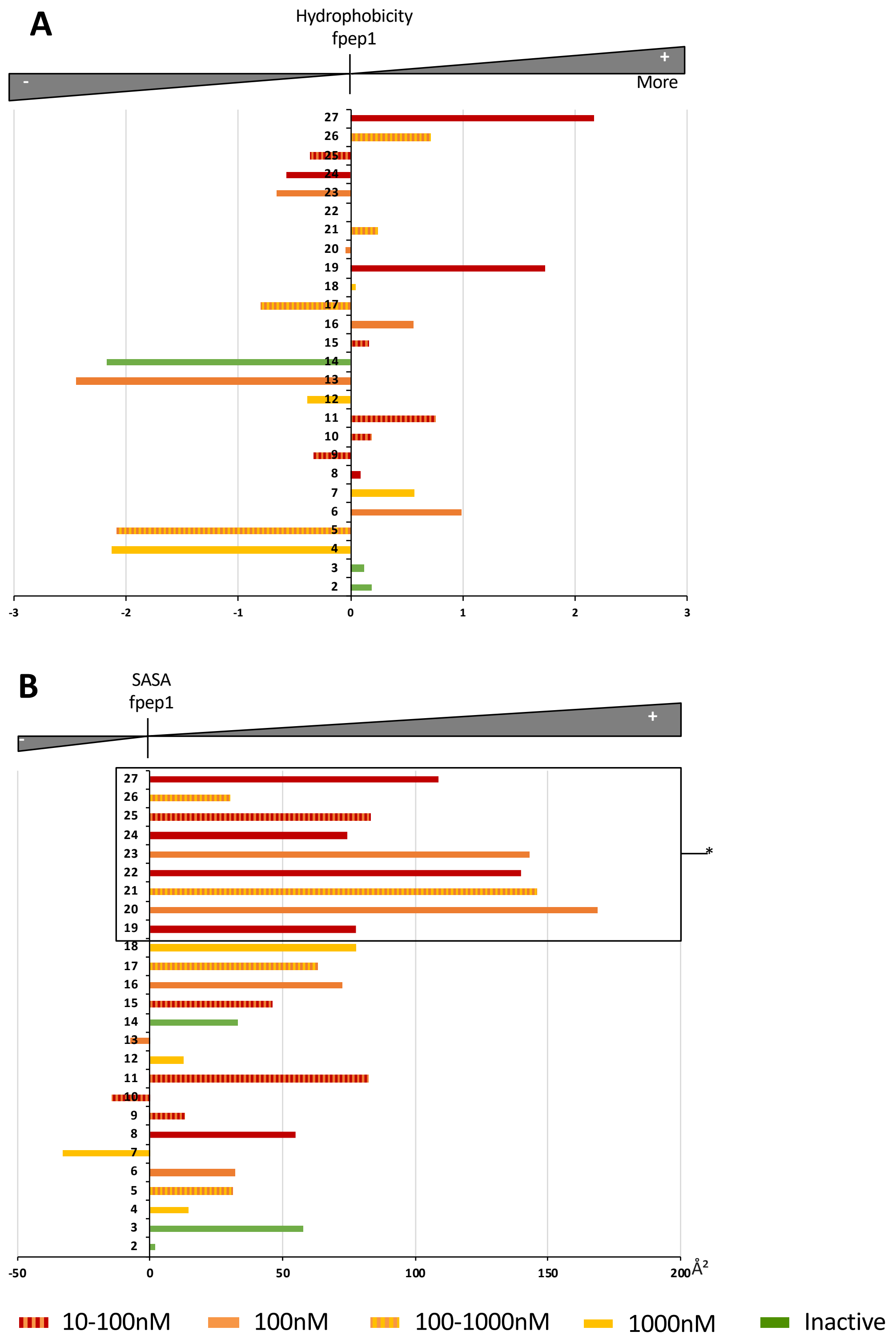

Supplementary Figure 3. *In silico* comparison of LogP (A) and solvent accessible surface area/ SASA (B) for fPep FP1 and FP2-27. The chemotactic trends are highlighted in the legend to contextualize the *in-silico* analysis. For the logP analysis (panel A), there is no clear link between chemotaxis and hydrophobicity. For the SASA analysis (panel B), there is a clear increase of the solvent accessible surface area; exceptions are seen for modifications with shorter aliphatic amino acid **FP7** (Nva) and **FP10** (tLeu).

* Position **L19-27**: SASA biased comparison with glycine

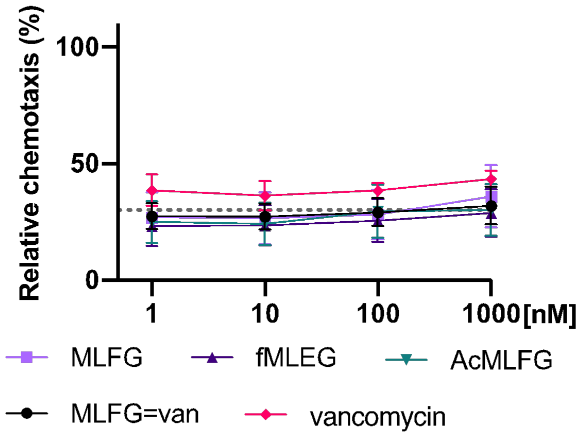

Supplementary Figure 4. Peptides that resulted in no recruitment of human neutrophils in a transwell assay. A transwell assay was used to determine the chemotaxis of human neutrophils to these peptides at 1, 10, 100, and 1000 nM. Chemotaxis was calculated relative to the no protein control and 100% chemotaxis set as the neutrophil recruitment observed to fMLFG at 100 nM for each donor, n=3 biologically independent experiments, error bars are SEM. Dotted line is the media only control recruitment level.

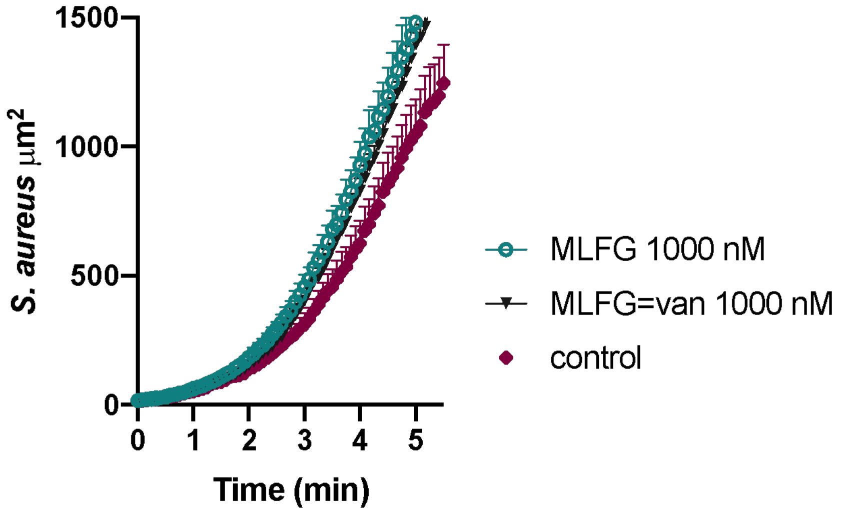

Supplementary Figure 5. *S. aureus* growth in the presence of MLFG linked to vancomycin in a microfluidic device. The growth of *S. aureus* expressing GFP was determined by area of GFP fluorescence within the microfluidic chamber in the presence of MLFG alone (**FP2**) or conjugated to vancomycin (MLFG=van, **C12**). Data are representative of 3 biological replicates, error bars are SEM.

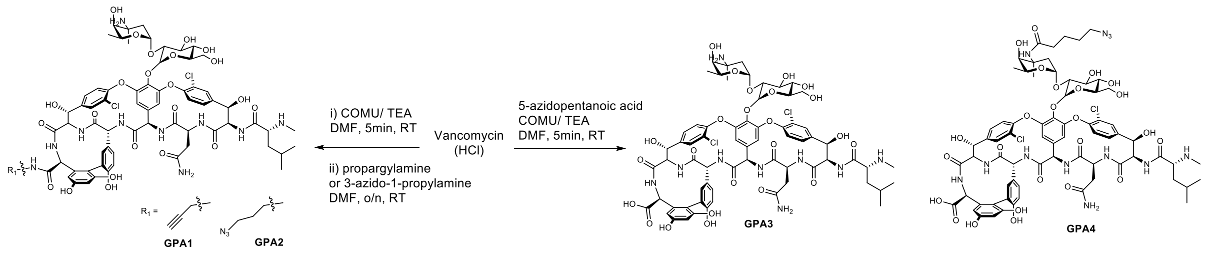

GPA No.	Abbreviated name	MW Theoretical (Da)	m/z Experimental (Da)	LC t_R (min)	Yield
**GPA1**	Van-C (propargyl amide)	1484.5	1486.2	11.5	49%
**GPA2**	Van-C (azidopropyl amide)	1529.5	1531.4	16.6	45%
**GPA3**	Van-N (azidopentyl amide)	1572.5	1574.3	11.4	26%
**GPA4**	Van-V (azidopentyl amide)	1572.5	1574.3	16.4	9%

Supplementary Figure 6. Summary of modified vancomycin (GPA1-4) chemical synthesis and characterization. For **GPA1** and **2**, Vancomycin (1 eq) and COMU (1 eq) were dissolved in DMF and activation of the C-terminal carboxylic acid was achieved upon addition of TEA (1 eq). After 5 minutes, propargylamine or 3-azidopropylamine was added to allow the formation of the amide bond. For **GPA3-4**, 5-azidopentanoic acid (1 eq) and COMU (1 eq) were dissolved in DMF and activation was achieved upon addition of TEA (1 eq). After 15 minutes, this solution was added to vancomycin dissolved in DMF to allow the formation of the amide bond on the methylated amine and the vancosamine. These reactions resulted in double incorporation of 5-azidopentanoic on both moieties, resulting in lower yields than for **GPA1** and **2.** In all reactions, the pH of the reaction was adjusted with TEA and the reaction monitored by LCMS until completion. Modified **GPA1-4** were isolated after precipitation in diethyl ether to give light brown powders and purified by preparative RP-HPLC. The identities of the regioisomers **GPA3** and **4** were confirmed by comparing the LCMS analysis after acidolysis with TFA. In the case of **GPA3**, acidolysis led to the characterization of the vancomycin aglycone bearing the azidopentyl moiety at the N-terminus (MW = 1267.4 Da), while **GPA4** acidolysis led to formation of the vancomycin aglycone (MW = 1142.4 Da).

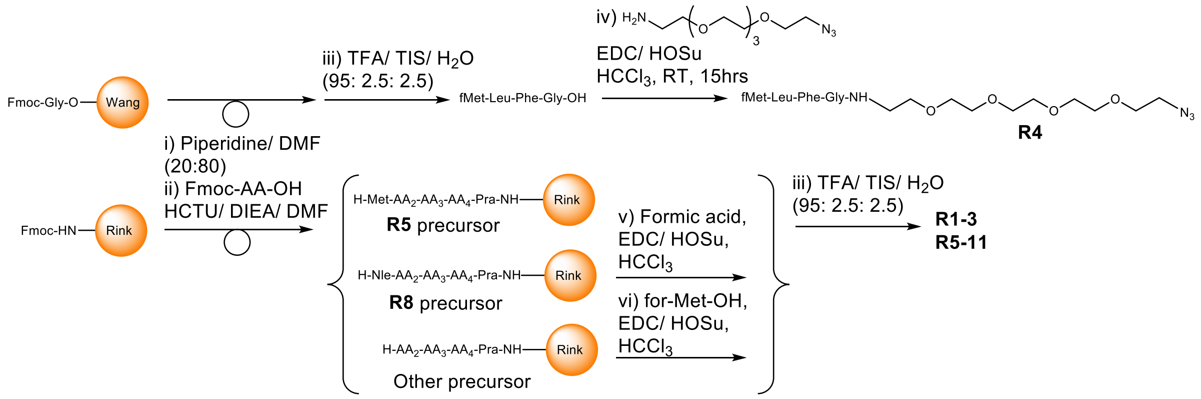

Peptide-R No.	Abbreviated name	MW Theoretical (Da)	m/z Experimental (Da)	LC t_R (min)	Yield
**R1**	fMLFG-Pra-NH_2	588.3	588.4	21.8	14%
**R2**	fMLFG-PEG3-Pra-NH_2	791.4	791.4	20.2	13%
**R3**	fMLFG-(PEG3)3-Pra-NH_2	994.5	995.2	19.7	15%
**R4**	fMLFG-PEG_4-azido	738.4	738.4	20.5	11%
**R5**	H-MLFG-Pra-NH_2	560.3	560.4	13.9	46%
**R6**	fM-Cha-FG-Pra-NH_2	628.3	628.5	21.8	31%*
**R7**	f-Nle-LFG-Pra-NH_2	570.3	570.3	24.5	34%*
**R8**	fM-Nle-FG-Pra-NH_2	588.3	588.2	23.3	34%*
**R9**	fML-Phe(4-F)-G-Pra-NH_2	606.3	606.7	23.8	32%
**R10**	fMLF-Pro-Pra-NH_2	628.3	628.3	23.5	41%
**R11**	fMLF-Lys-Pra-NH_2	659.4	659.4	20.7	40%
**R12**	fMLF-Leu-Pra-NH_2	644.3	644.2	27.8	25%*
**R13**	fML-Tyr-G- Pra-NH_2	604.3	604.4	17.5	43%

Supplementary Figure 7. Summary of fPep chemical synthesis and characterization of fPep precursors R1-12. The original strategy used for the preparation of **R4** was not efficient since it required an extra step with the subsequent coupling of azido-PEG_4-amine. Later on, all the peptides were readily prepared with a C-terminal propargylglycine (**R13 and R5-13**). *Crude compounds dissolved in DMSO prior preparative RP-HPLC purification.

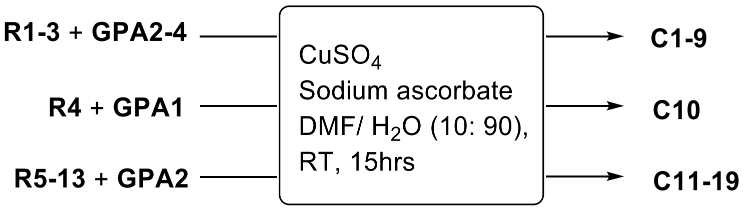

Conjugate No.	Abbreviated name	MW Theoretical (Da)	m/z Experimental (Da)	LC t_R (min)	Yield
**C1**	Van-C(fMLFG-Tz)	2117.8	2117.7	14.3	47%
**C2**	Van-C(fMLFG-PEG3-Tz)	2320.9	2322.9	14.9	38%
**C3**	Van-C(fMLFG-(PEG3)2-Tz)	2524.0	2526.2	15.4	33%
**N4**	Van-N(fMLFG-Tz)	2160.8	2162.8	19.6	36%
**N5**	Van-N(fMLFG-PEG3-Tz)	2363.9	2366.1	19.3	28%
**N6**	Van-N(fMLFG-(PEG3)2-Tz)	2566.9	2569.3	19.1	34%
**V7**	Van-V(fMLFG-Tz)	2160.8	2162.8	15.0	20%
**V8**	Van-V(fMLFG-PEG3-Tz)	2363.9	2366.4	15.8	30%
**V9**	Van-V(fMLFG-(PEG3)2-Tz)	2566.9	2569.0	16.4	17%
**C10**	Van-C(fMLFG-PEG4-Tz)	2222.8	2225.0	10.8	42%
**C11**	Van-C(fM-Cha-FG-Tz)	2157.8	2160.3	15.8	36%
**C12**	Van-C(H-MLFG-Tz)	2089.8	2087.8	17.7	45%
**C13**	Van-C(fNle-LFG-Tz)	2099.8	2101.8	16.9	37%
**C14**	Van-C(fM-Nle-FG-Tz)	2117.8	2119.8	15.9	42%
**C15**	Van-C(fML-Phe(4-F)-G-Tz)	2135.8	2137.8	16.4	47%
**C16**	Van-C(fMLF-Pro-Tz)	2157.8	2159.7	16.3	46%
**C17**	Van-C(fMLF-Lys-Tz)	2188.8	2190.0	14.2	44%
**C18**	Van-C(fMLF-Leu-Tz)	2173.8	2175.6	22.2	47%
**C19**	Van-C(fML-Tyr-G-Tz)	2133.8	2135.6	17.5	46%

Supplementary Figure 8. Summary of the chemical synthesis and characterization of conjugated fPep to vancomycin. All fPeps were readily dissolved in DMF (preferred to DMSO, which increased the conversion of methionine (Met) into methionine oxide (Met(O))). Chemical reaction: $CuSO_4$ (4 eq), Sodium ascorbate (10 eq), H_2O: DMF (90: 10), RT, 15h.[2]

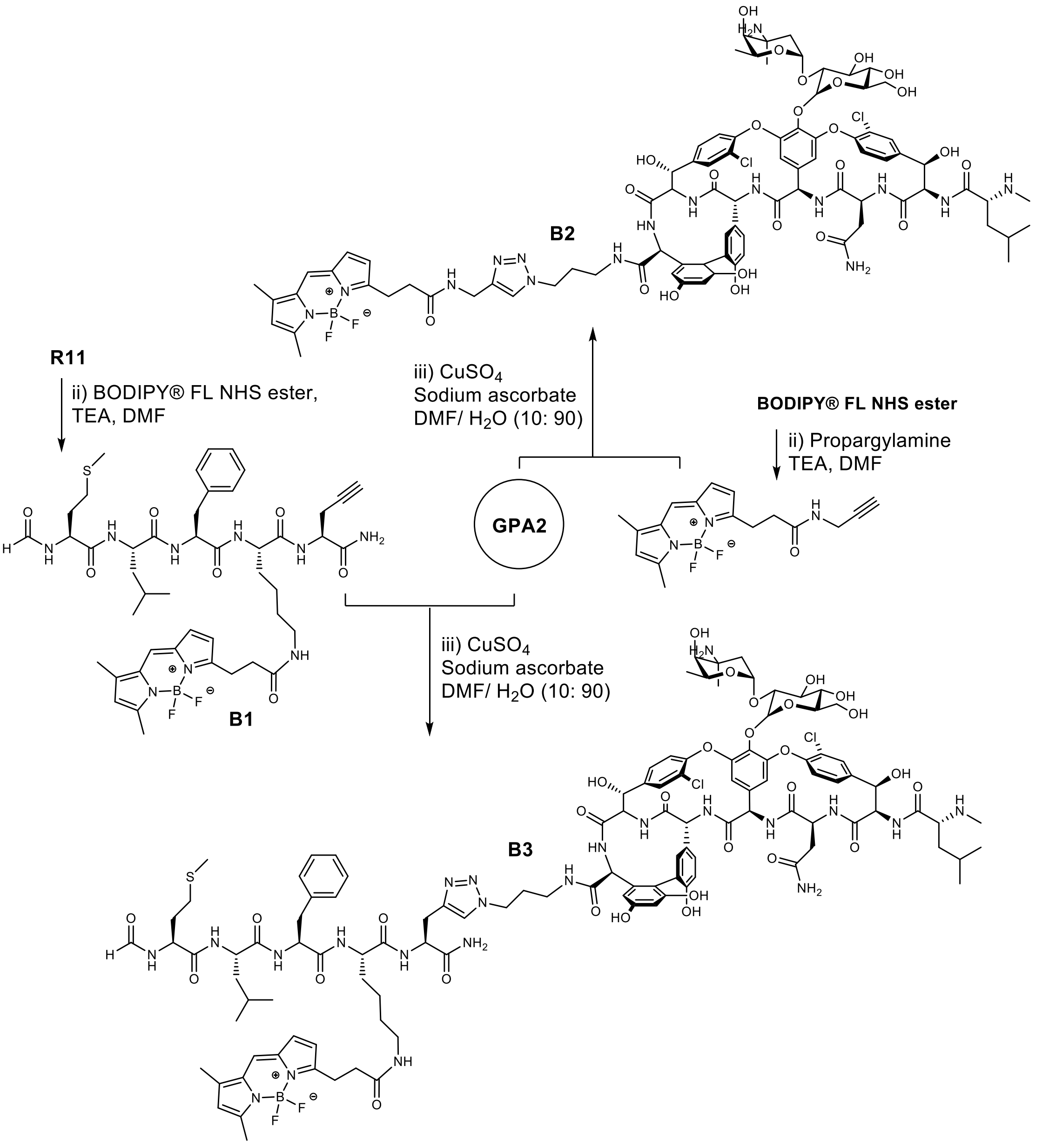

BODIPY No.	Abbreviated name	MW Theoretical (Da)	m/z Experimental (Da)	LC t_R (min)	Yield
B1	fMLF-Lys(B*)-NH2	933.4	933.4 [b]	26.1	84%
B2	Van-C(B*)	1860.7	1860.2 [b]	20.2	65%[#]
B3	Van-C-(fMLF Lys(B*)-Tz)	2462.9499 ($C_{115}H_{143}BCl_2F_2N_{22}O_{30}S$)	2462.9807 [a] (12.5 Δppm)	26.6	ND

Supplementary Figure 9. Summary of the chemical synthesis and characterization of fluorescently labelled compounds (B1-3). Chemical reactions: i) BODIPY® FL NHS ester, **R11**, TEA, DMF, RT, 2h, affording **B1**; ii) BODIPY® FL NHS ester, propagylamine, TEA, DMF, RT, 2h, affording the alkyne-BODIPY; iii) $CuSO_4$, Sodium ascorbate, H_2O: DMF (90; 10), RT, 15h. **B2** and **B3** were obtained by reacting **GPA1** with the alkyne- BODIPY® and **B1**. * BODIPY. [#] Yield calculated over 2 steps. ND: Not determined. [a] Determined by HRMS. [b] Determined by LCMS.

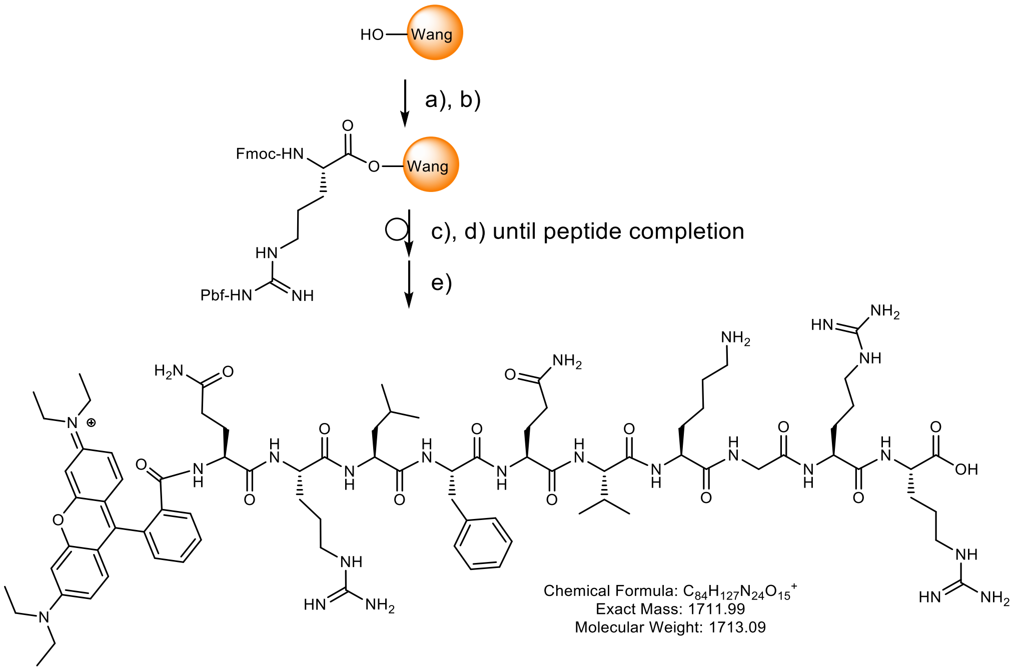

Supplementary Figure 10. Preparation of Rhodamine B-PB10 peptide inhibitor of FRP2 by solid phase peptide synthesis. Chemical reactions: a) Fmoc-L-Arg(Pbf)-OH, DIC, DMAP, DMF, RT, 15 h; b) acetic anhydride, DMAP, DMF, RT, 15 min; c) Piperidine/ DMF (20: 80), RT, 15 min; d) Rhodamine B, DIC, Oxyma, DMF, RT, 1 h; e) TFA/ TIS/ H_2O (95: 2.5: 2.5), RT, 2 h[3]. Yield of pure compound 1.3%.

FP1

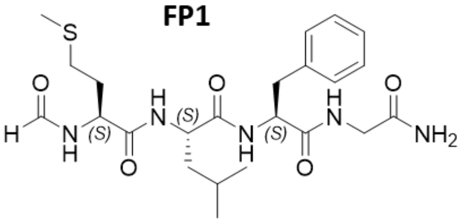

Chemical Formula: $C_{23}H_{35}N_5O_5S$
Exact Mass: 493.24
Molecular Weight: 493.62

Log P: -0.95

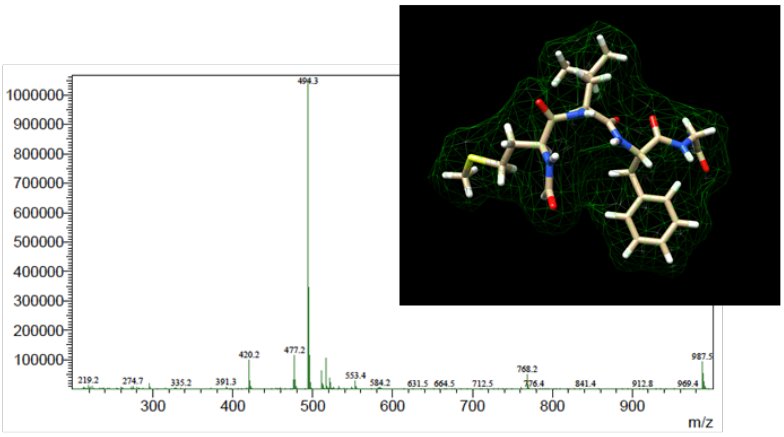

FP2

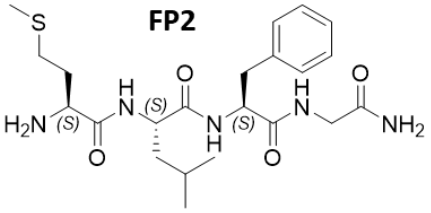

Chemical Formula: $C_{22}H_{35}N_5O_4S$
Exact Mass: 465.24
Molecular Weight: 465.61

Log P: -0.77

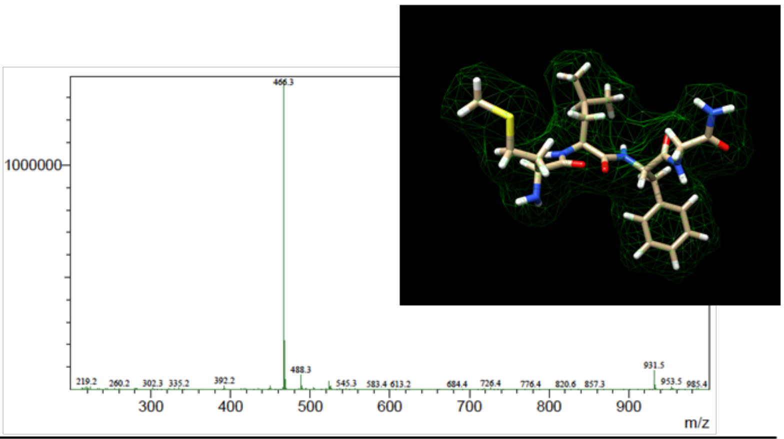

FP3

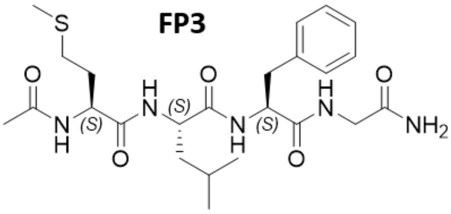

Chemical Formula: $C_{24}H_{37}N_5O_5S$
Exact Mass: 507.25
Molecular Weight: 507.65

Log P: -0.83

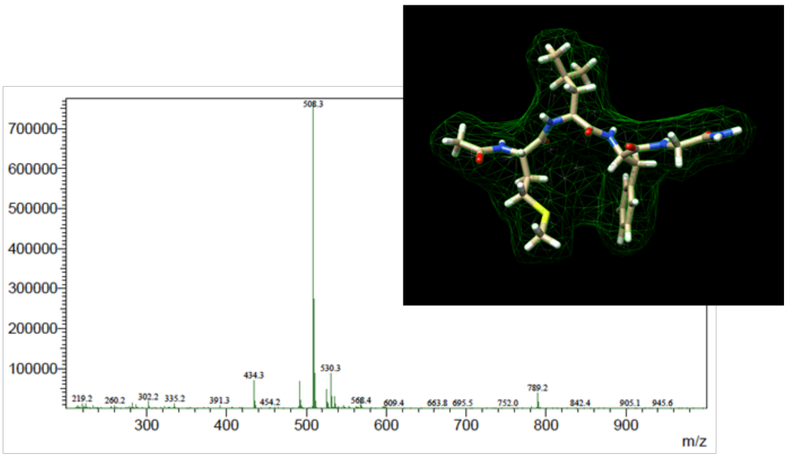

FP4

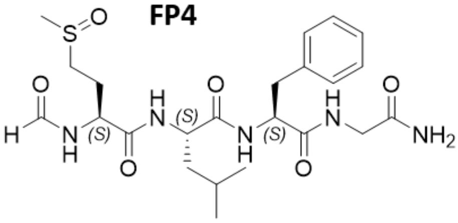

Chemical Formula: $C_{23}H_{35}N_5O_6S$
Exact Mass: 509.23
Molecular Weight: 509.62

Log P: -3.08

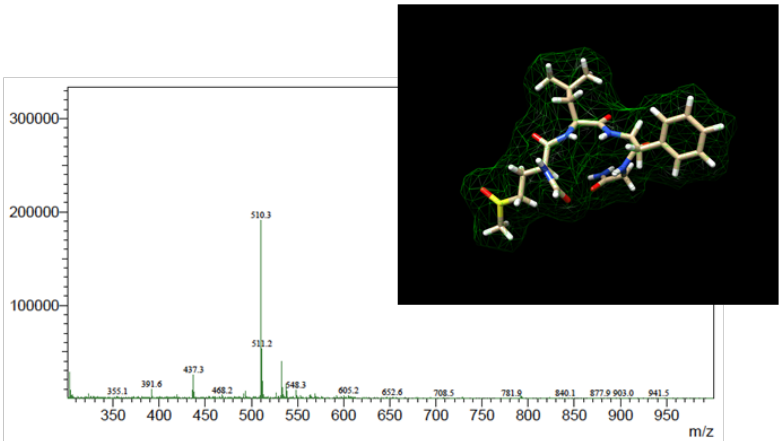

Supplementary Figure 11. Characterisation of fPeptide library

FP5

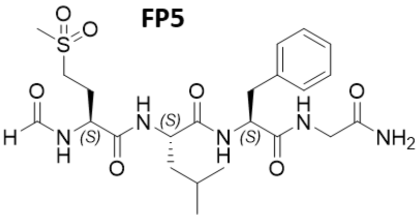

Chemical Formula: $C_{23}H_{35}N_5O_7S$
Exact Mass: 525.23
Molecular Weight: 525.62

Log P: -3.03

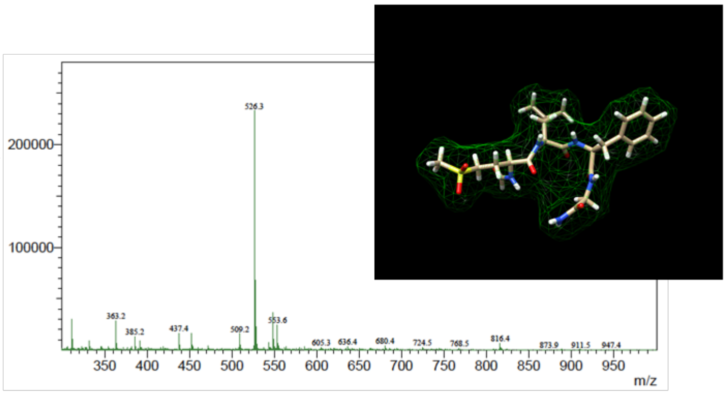

FP6

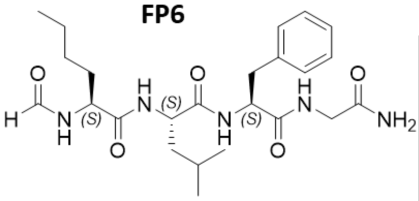

Chemical Formula: $C_{24}H_{37}N_5O_5$
Exact Mass: 475.28
Molecular Weight: 475.59

Log P: 0.04

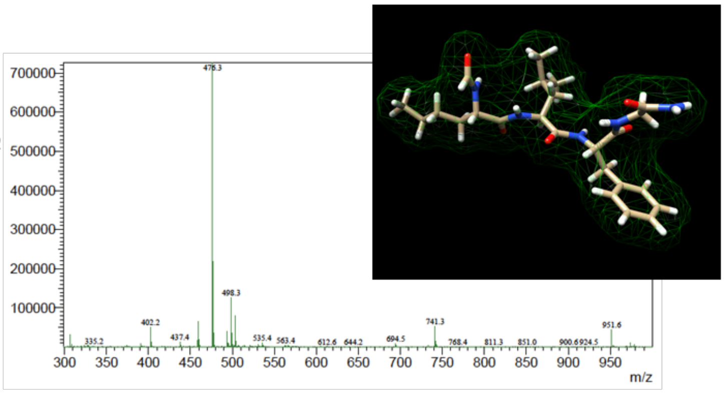

FP7

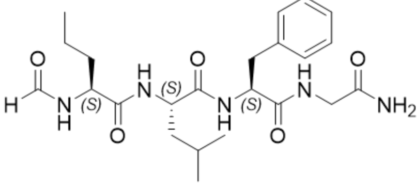

Chemical Formula: $C_{23}H_{35}N_5O_5$
Exact Mass: 461.26
Molecular Weight: 461.56

Log P: -0.38

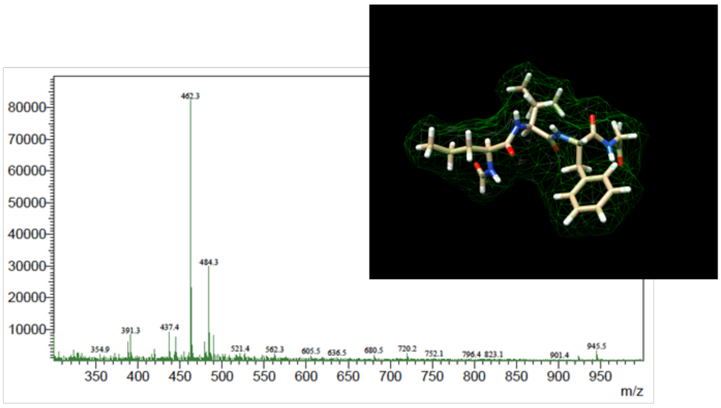

FP8

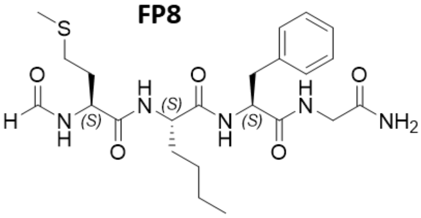

Chemical Formula: $C_{23}H_{35}N_5O_5S$
Exact Mass: 493.24
Molecular Weight: 493.62

Log P: -0.86

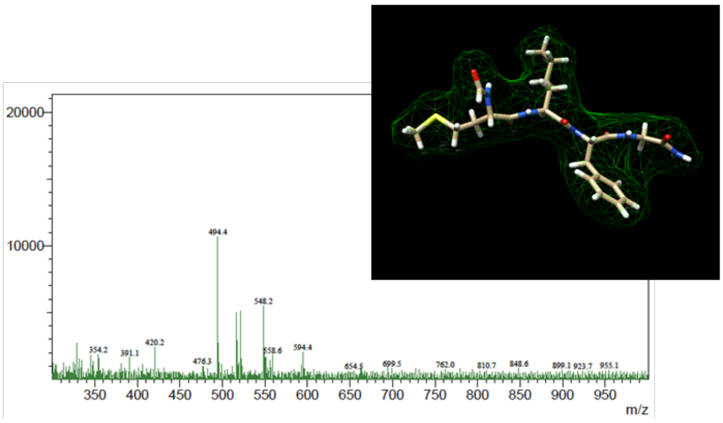

Supplementary Figure 11. Characterisation of fPeptide library- continued

FP9

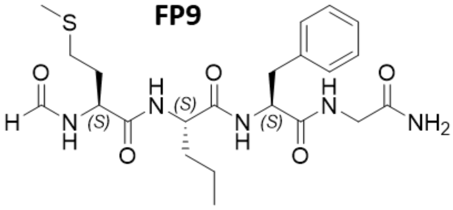

Chemical Formula: $C_{22}H_{33}N_{5}O_{5}S$
Exact Mass: 479.22
Molecular Weight: 479.60

Log P: -1.28

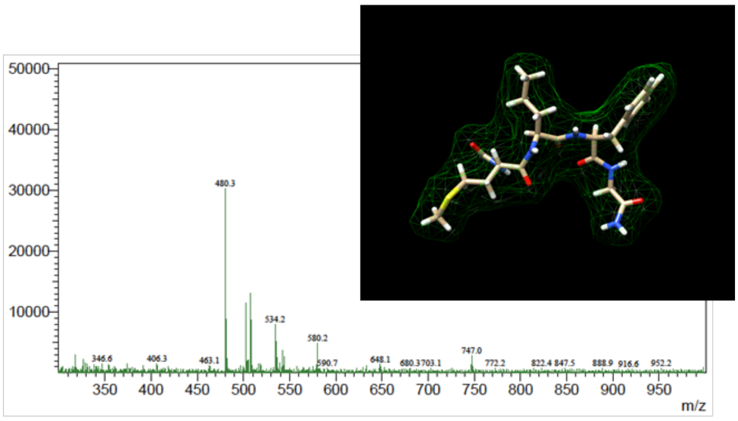

FP10

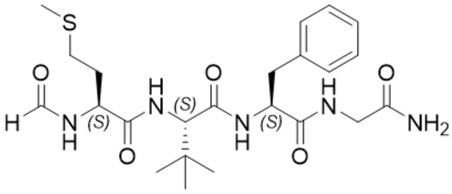

Chemical Formula: $C_{23}H_{35}N_{5}O_{5}S$
Exact Mass: 493.24

Log P: -0.76

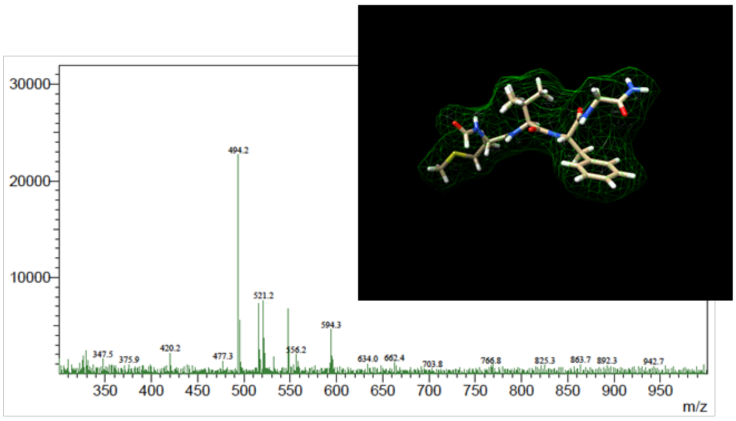

FP11

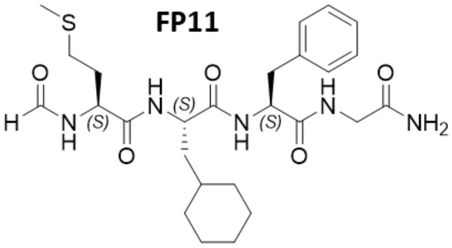

Chemical Formula: $C_{26}H_{39}N_{5}O_{5}S$
Exact Mass: 533.27
Molecular Weight: 533.69

Log P: -0.19

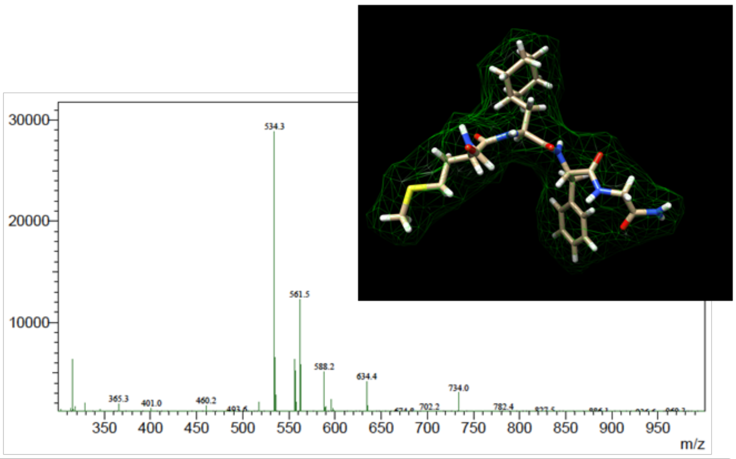

FP12

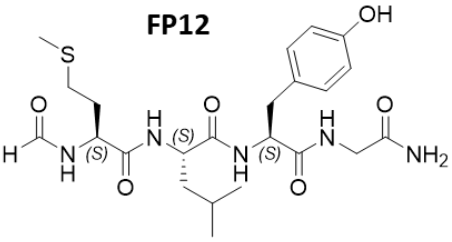

Chemical Formula: $C_{23}H_{35}N_{5}O_{6}S$
Exact Mass: 509.23
Molecular Weight: 509.62

Log P: -1.34

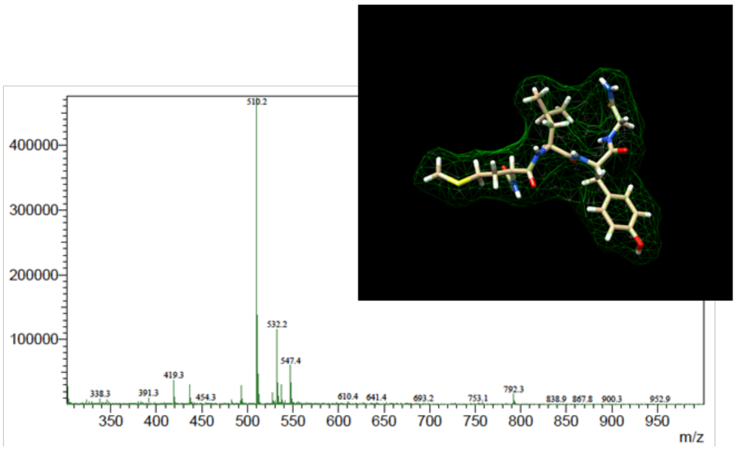

Supplementary Figure 11. Characterisation of fPeptide library- continued

FP13

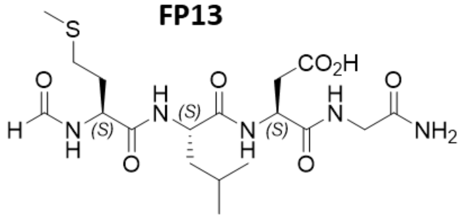

Chemical Formula: $C_{18}H_{31}N_5O_7S$
Exact Mass: 461.19
Molecular Weight: 461.53

Log P: -3.4

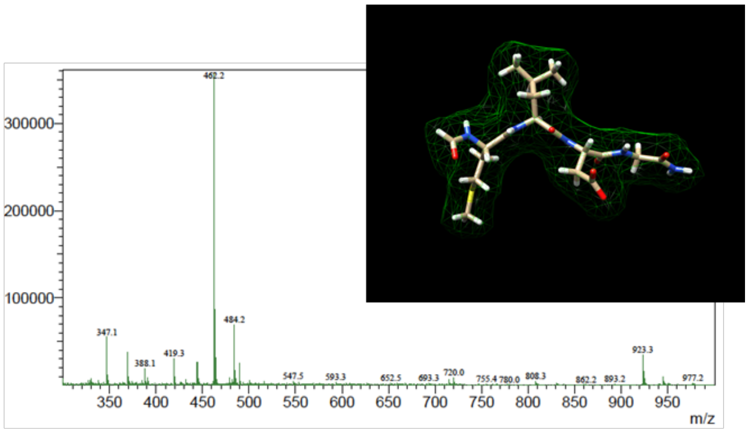

FP14

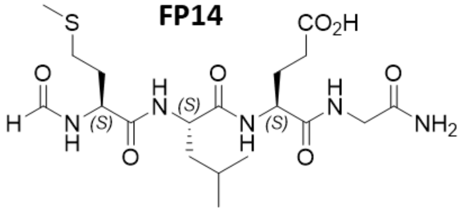

Chemical Formula: $C_{19}H_{33}N_5O_7S$
Exact Mass: 475.21
Molecular Weight: 475.56

Log P: -3.12

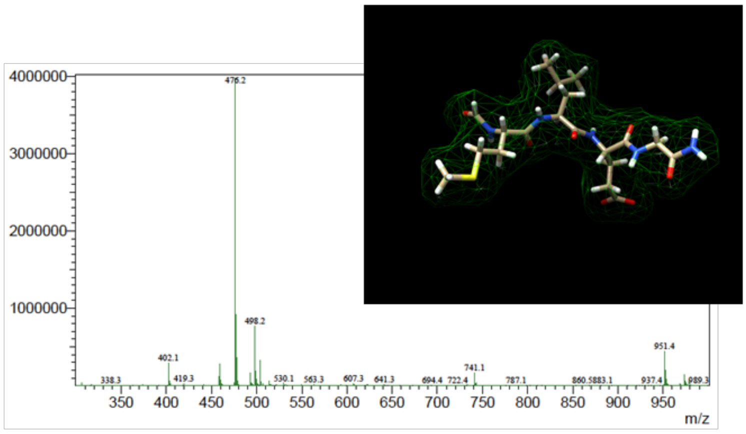

FP15

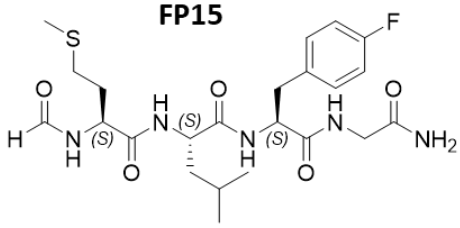

Chemical Formula: $C_{23}H_{34}FN_5O_5S$
Exact Mass: 511.23
Molecular Weight: 511.61

Log P: -0.79

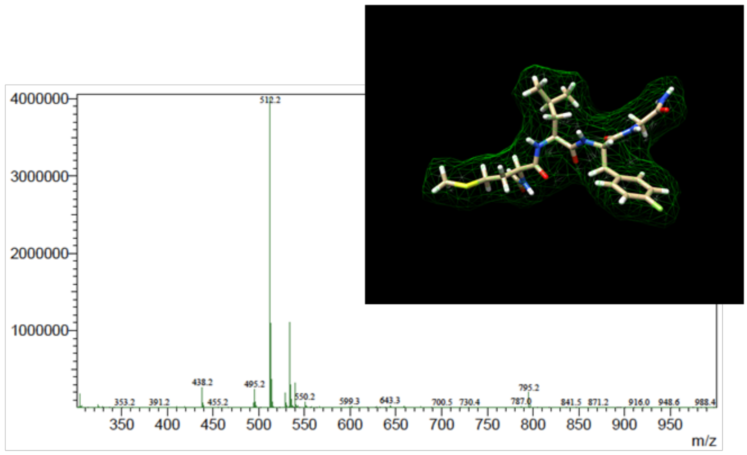

FP16

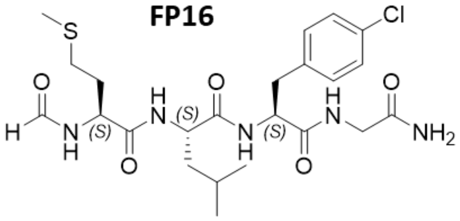

Chemical Formula: $C_{23}H_{34}ClN_5O_5S$
Exact Mass: 527.20
Molecular Weight: 528.07

Log P: -0.39

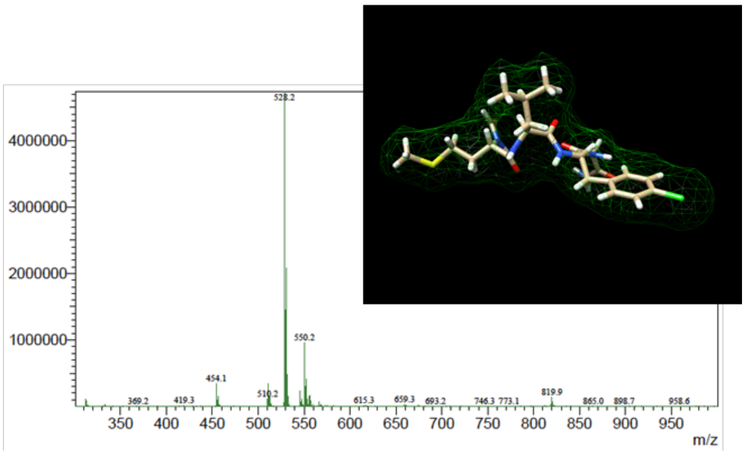

Supplementary Figure 11. Characterisation of fPeptide library- continued

FP17

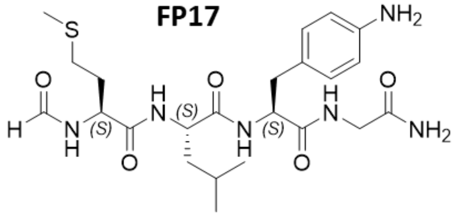

Chemical Formula: $C_{23}H_{36}N_6O_5S$
Exact Mass: 508.25
Molecular Weight: 508.64

Log P: -1.75

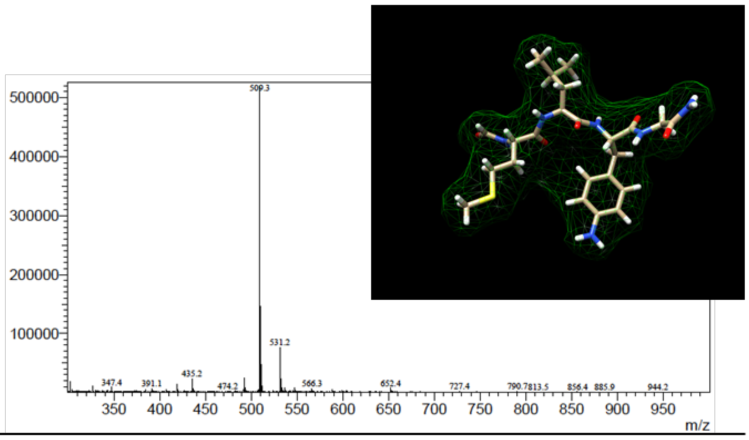

FP18

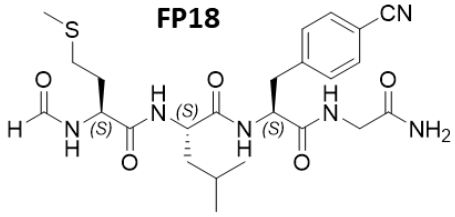

Chemical Formula: $C_{24}H_{34}N_6O_5S$
Exact Mass: 518.23
Molecular Weight: 518.63

Log P: -0.91

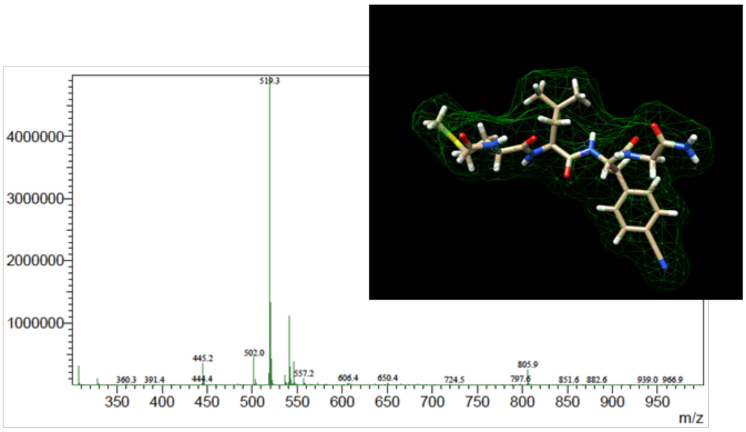

FP19

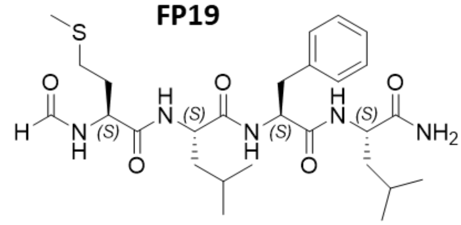

Chemical Formula: $C_{27}H_{43}N_5O_5S$
Exact Mass: 549.30
Molecular Weight: 549.73

Log P: 0.78

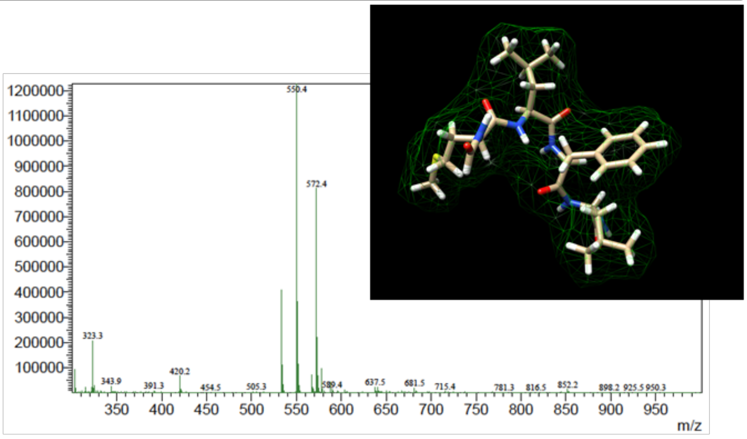

FP20

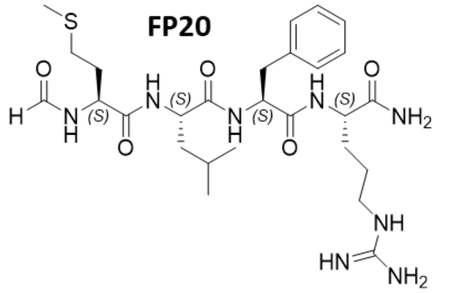

Chemical Formula: $C_{27}H_{44}N_8O_5S$
Exact Mass: 592.32
Molecular Weight: 592.76

Log P: -0.99

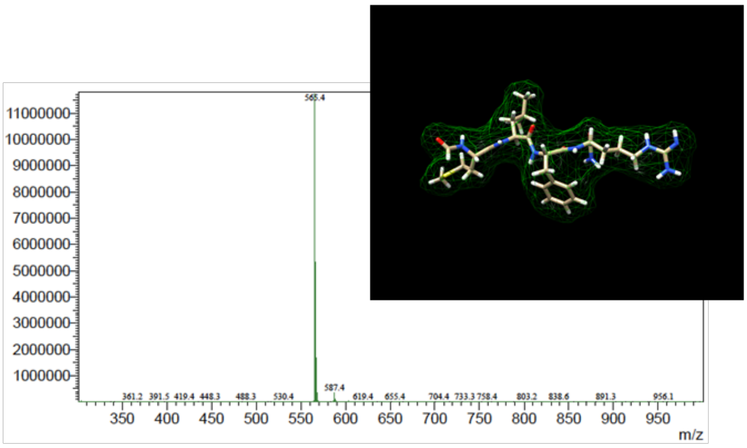

Supplementary Figure 11. Characterisation of fPeptide library- continued

FP21

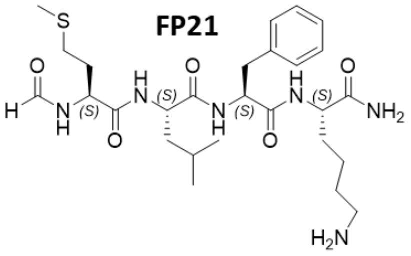

Chemical Formula: $C_{27}H_{44}N_6O_5S$
Exact Mass: 564.31
Molecular Weight: 564.75

Log P: -0.71

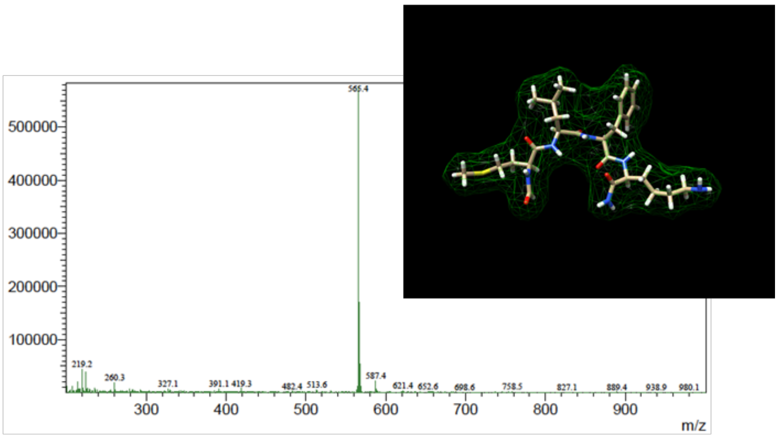

FP22

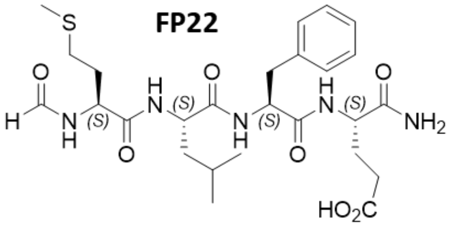

Chemical Formula: $C_{26}H_{39}N_5O_7S$
Exact Mass: 565.26
Molecular Weight: 565.69

Log P: -0.95

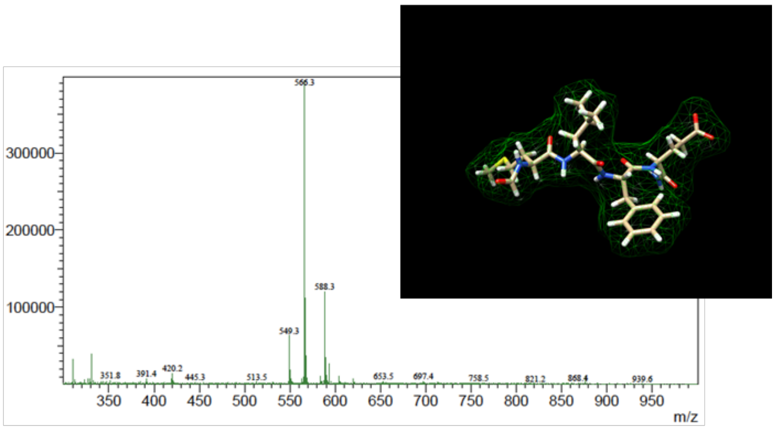

FP23

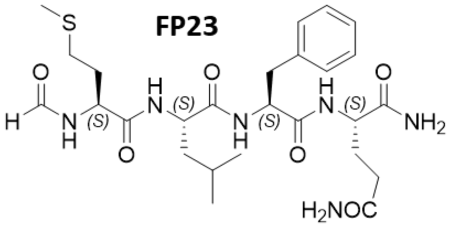

Chemical Formula: $C_{26}H_{40}N_6O_6S$
Exact Mass: 564.27
Molecular Weight: 564.70

Log P: -1.61

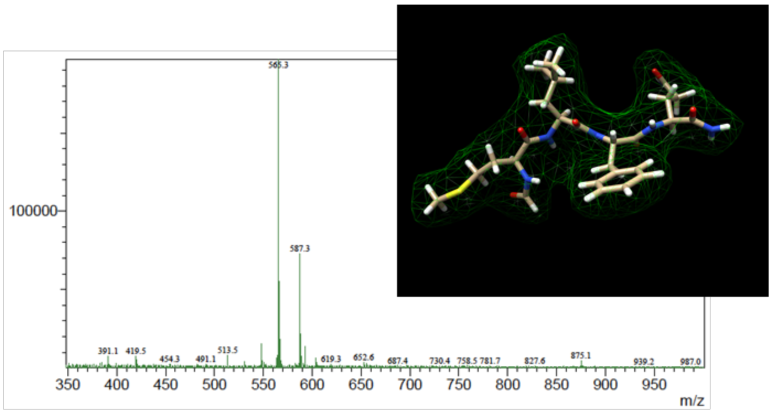

FP24

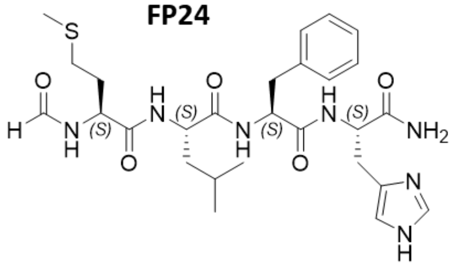

Chemical Formula: $C_{27}H_{39}N_7O_5S$
Exact Mass: 573.27
Molecular Weight: 573.71

Log P: -1.52

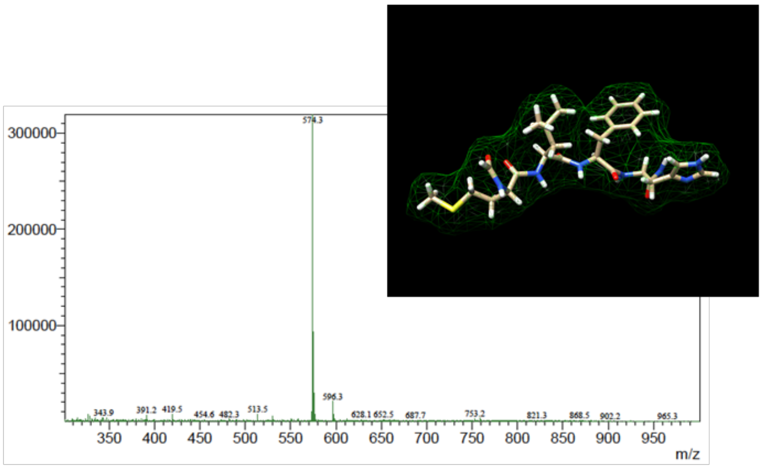

Supplementary Figure 11. Characterisation of fPeptide library- continued

FP25

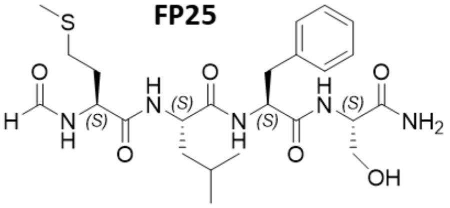

Chemical Formula: $C_{24}H_{37}N_5O_6S$
Exact Mass: 523.25
Molecular Weight: 523.65

Log P: -1.31

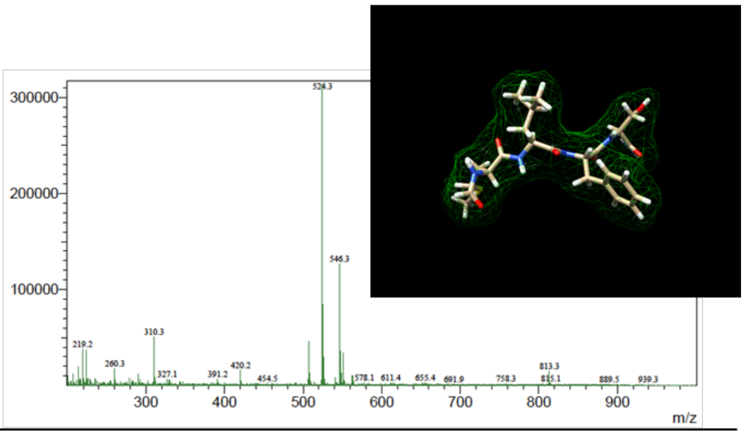

FP26

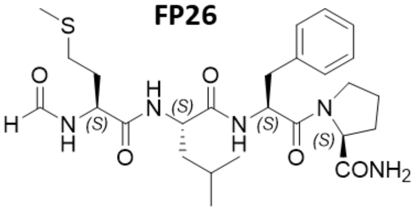

Chemical Formula: $C_{26}H_{39}N_5O_5S$
Exact Mass: 533.27
Molecular Weight: 533.69

Log P: -0.24

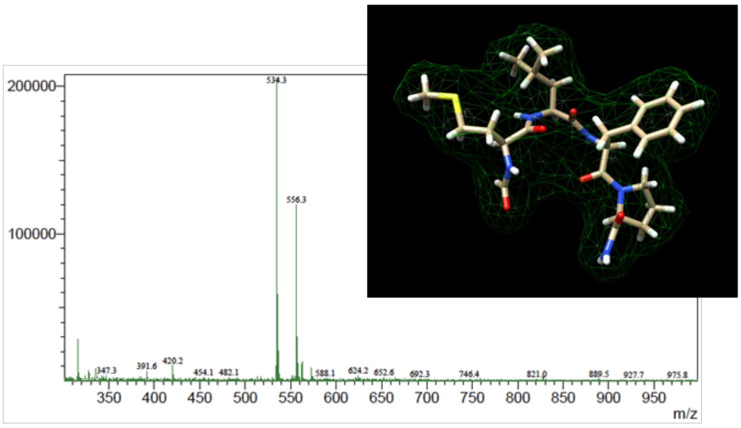

FP27

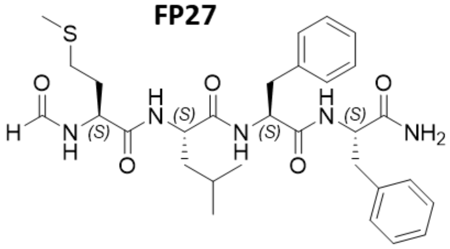

Chemical Formula: $C_{30}H_{41}N_5O_5S$
Exact Mass: 583.28
Molecular Weight: 583.75

Log P: 1.22

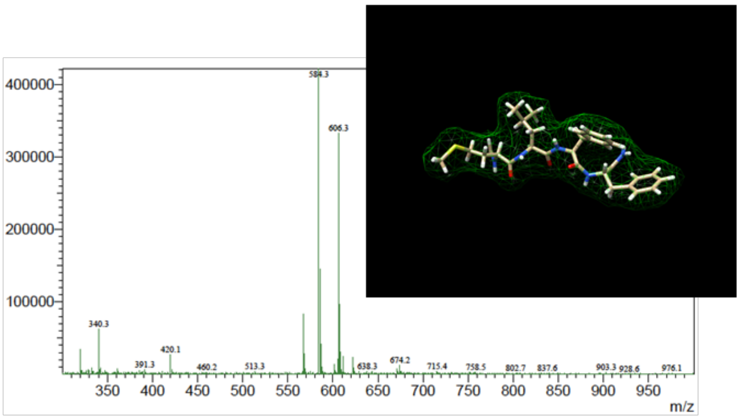

Supplementary Figure 11. Characterisation of fPeptide library- continued
Structure, logP, solvent accessible surface area (SASA), and LC-MS data for each of the fPeptides (**FP1-27**) made for the library.

GPA1
Van-C(propargyl amide)

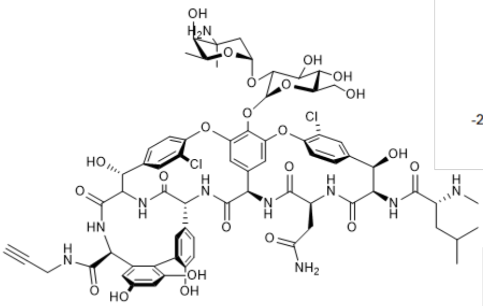

Chemical Formula: $C_{69}H_{78}Cl_2N_{10}O_{23}$
Exact Mass: 1484.46
Molecular Weight: 1486.33

Expected MW – 1484.5 Da;
Found m/z – 1486.2 Da.

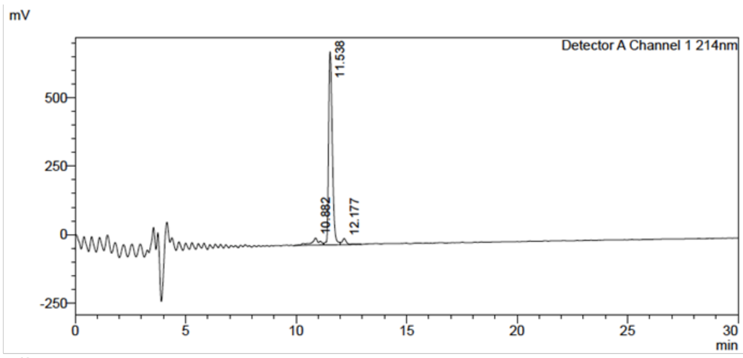

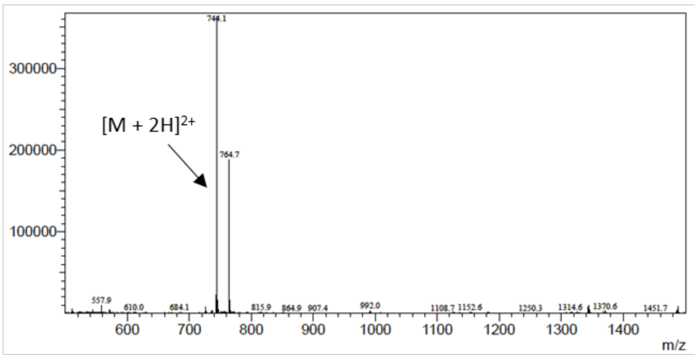

GPA2
Van-C(azidopropyl amide)

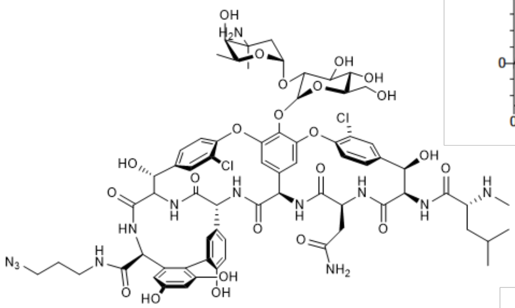

Chemical Formula: $C_{69}H_{81}Cl_2N_{13}O_{23}$
Exact Mass: 1529.49
Molecular Weight: 1531.38

Expected MW – 1529.5 Da;
Found m/z – 1531.4 Da.

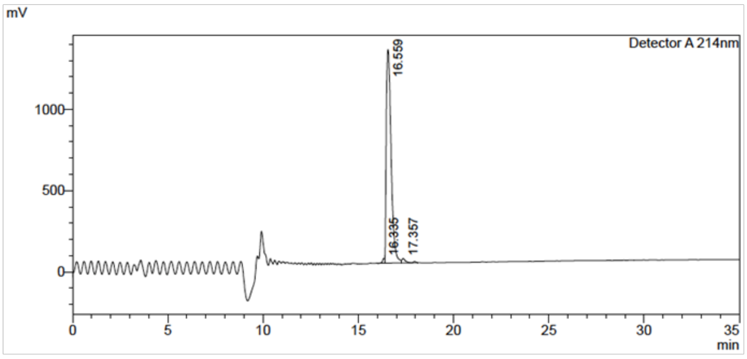

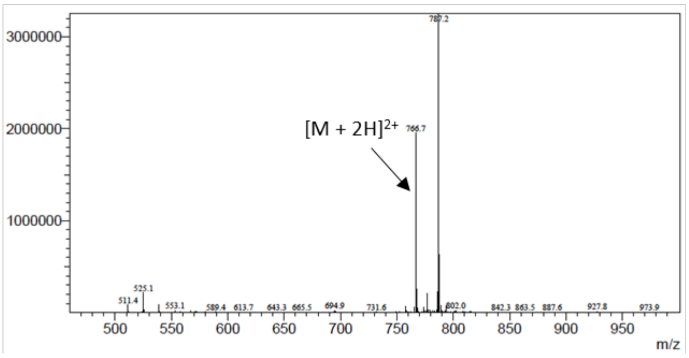

Supplementary Figure 12. Characterisation of vancomycin precursors- continued
Structure, and LC-MS profiles for each of the vancomycin precursors (**GPA 1-4**) used in this study.

GPA3
Van-N(azidopentyl amide)

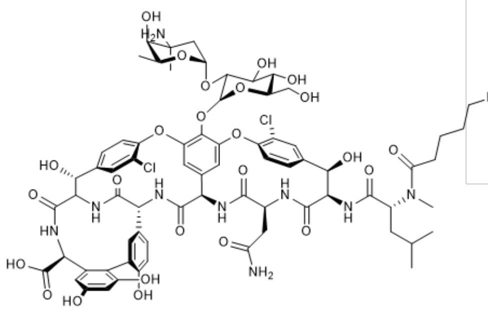

Chemical Formula: $C_{71}H_{82}Cl_2N_{12}O_{25}$
Exact Mass: 1572.49
Molecular Weight: 1574.40

Expected MW – 1572.5 Da;
Found m/z – 1574.3 Da.

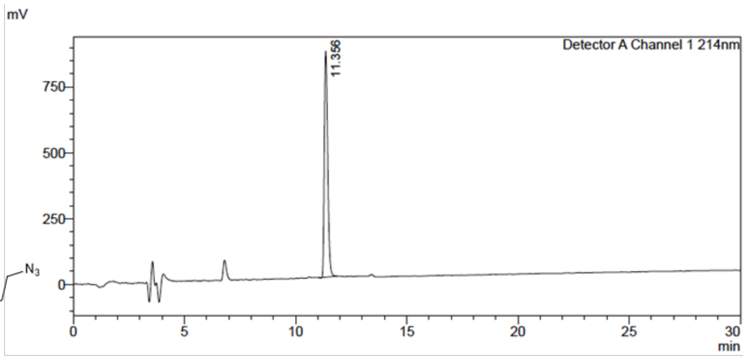

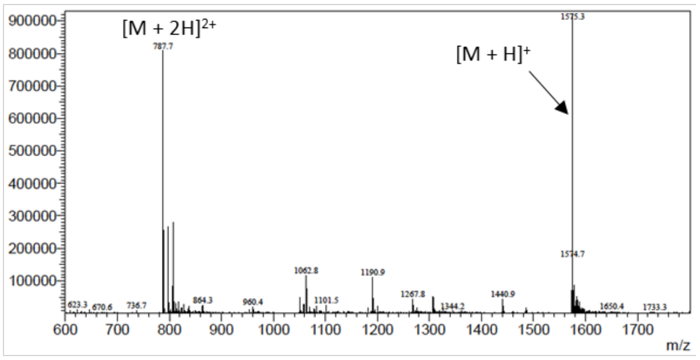

GPA4
Van-V(azidopentyl amide)

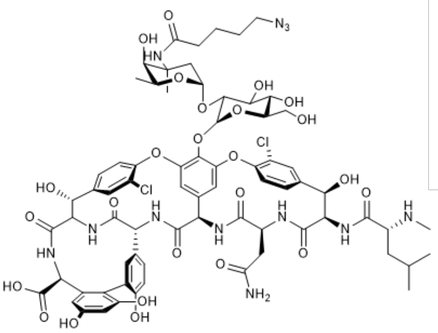

Chemical Formula: $C_{71}H_{82}Cl_2N_{12}O_{25}$
Exact Mass: 1572.49
Molecular Weight: 1574.40

Expected MW – 1572.5 Da;
Found m/z – 1574.3 Da.

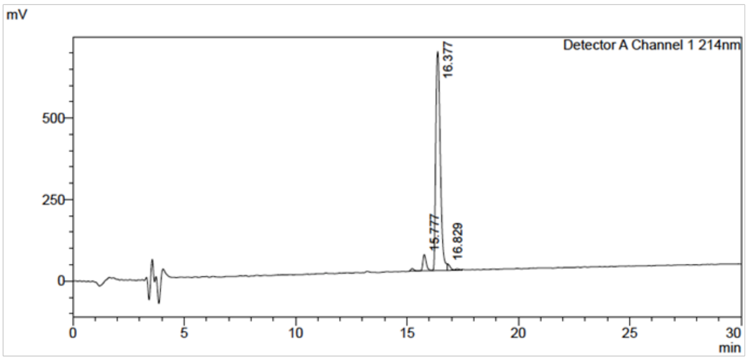

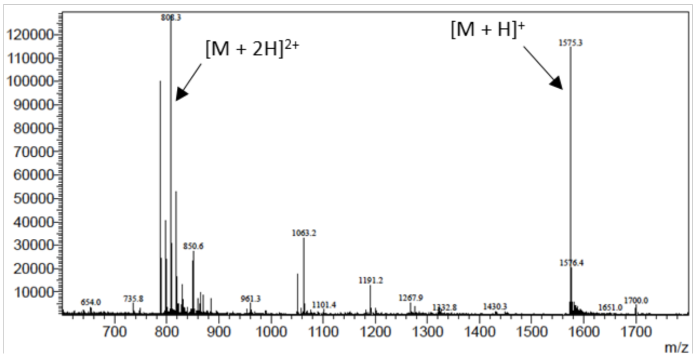

Supplementary Figure 12. Characterisation of vancomycin precursors- continued
Structure, and LC-MS profiles for each of the vancomycin precursors (**GPA 1-4**) used in this study.

R1
fMLFG-Pra-NH_2

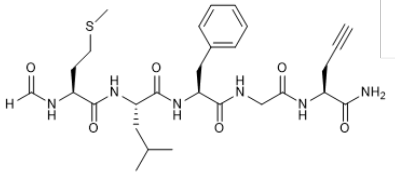

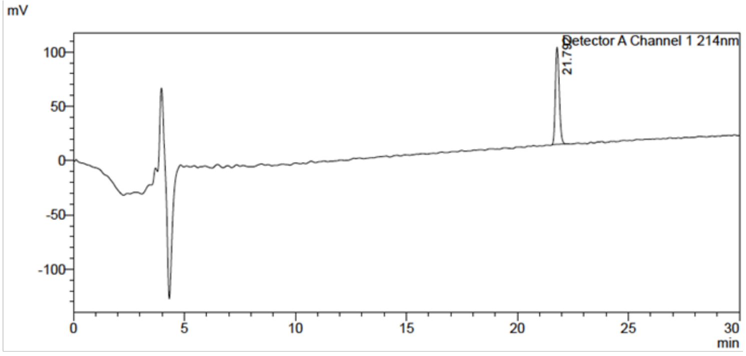

Chemical Formula: $C_{28}H_{40}N_6O_6S$
Exact Mass: 588.27
Molecular Weight: 588.72

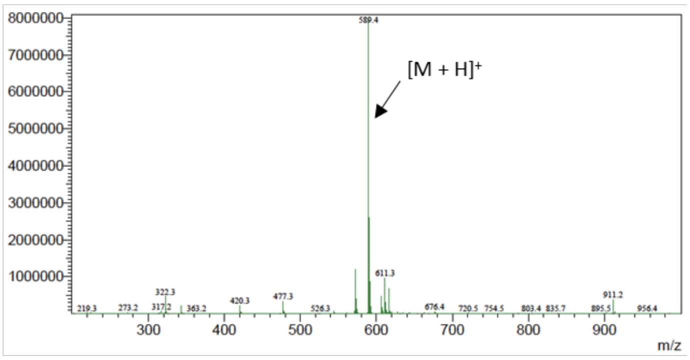

Expected MW – 588.3 Da;
Found m/z – 588.4 Da.

R2
fMLFG-PEG3-Pra-NH_2

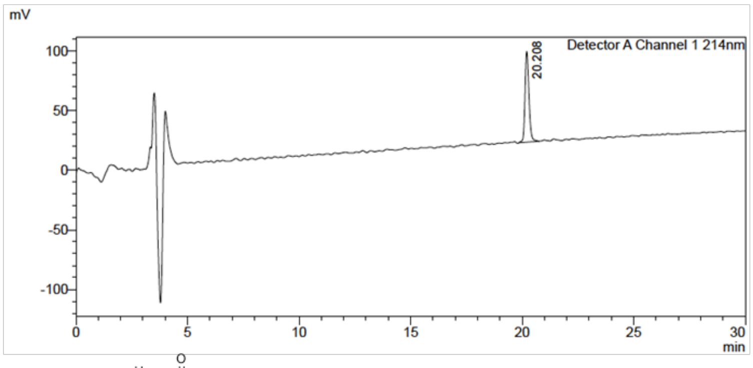

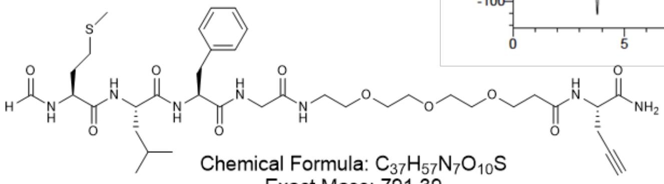

Chemical Formula: $C_{37}H_{57}N_7O_{10}S$
Exact Mass: 791.39
Molecular Weight: 791.96

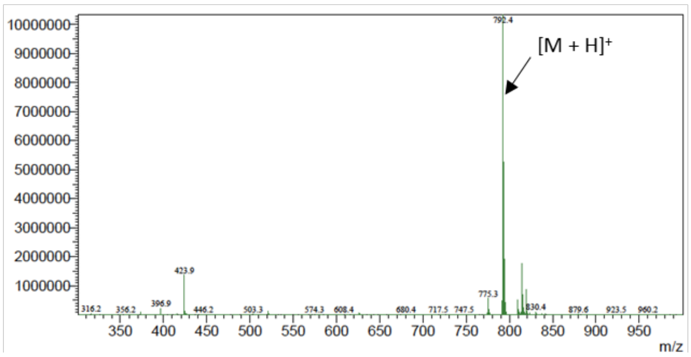

Expected MW – 791.4 Da;
Found m/z – 791.4 Da.

Supplementary Figure 13. Characterisation of fPeptide precursors- continued
Structure, and LC and MS profiles for each of the fPeptide precursors (**R1-13**) used in this study.

R3
fMLFG-(PEG3)2-Pra-NH_2

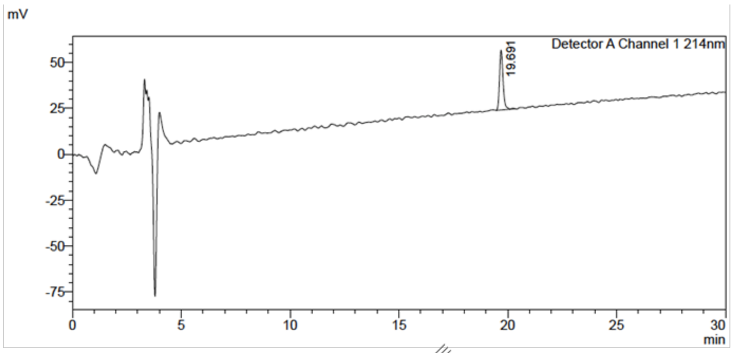

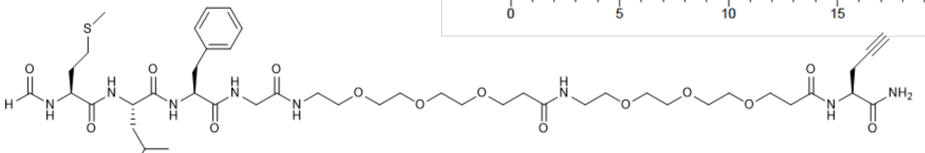

Chemical Formula: $C_{46}H_{74}N_8O_{14}S$
Exact Mass: 994.50
Molecular Weight: 995.20

Expected MW – 994.5 Da;
Found m/z – 995.2 Da.

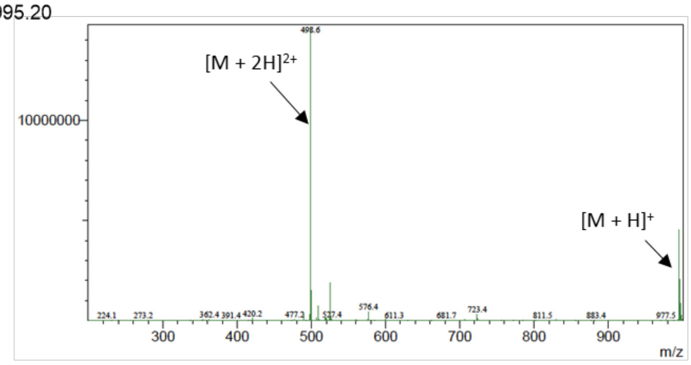

R4
fMLFG-PEG4-azido

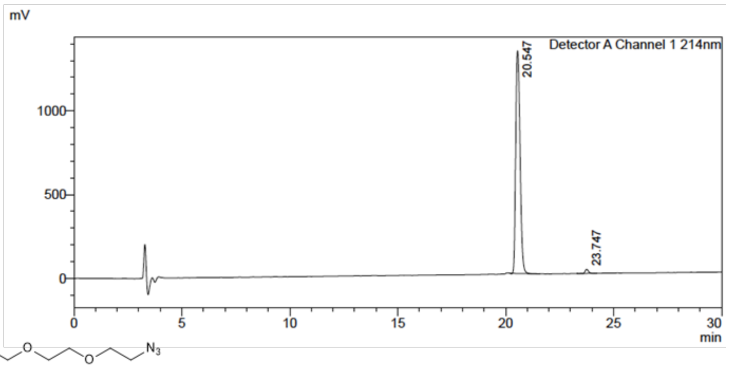

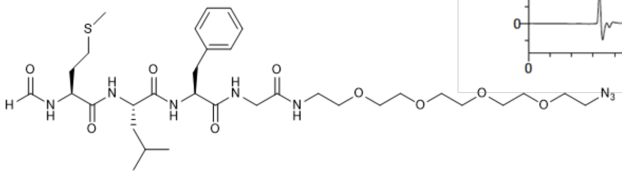

Chemical Formula: $C_{33}H_{54}N_8O_9S$
Exact Mass: 738.37
Molecular Weight: 738.90

Expected MW – 738.4 Da;
Found m/z – 738.4 Da.

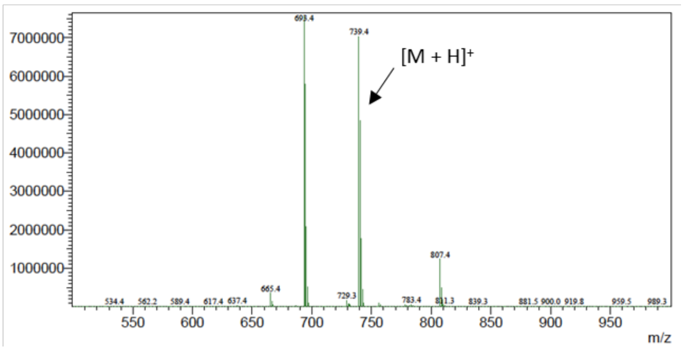

Supplementary Figure 13. Characterisation of fPeptide precursors- continued
Structure, and LC and MS profiles for each of the fPeptide precursors (**R1-13**) used in this study.

R5
H-MLFG-Pra-NH_2

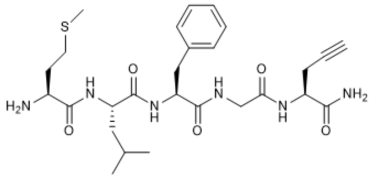

Chemical Formula: $C_{27}H_{40}N_6O_5S$
Exact Mass: 560.28
Molecular Weight: 560.71

Expected MW – 560.3 Da;
Found m/z – 560.4 Da.

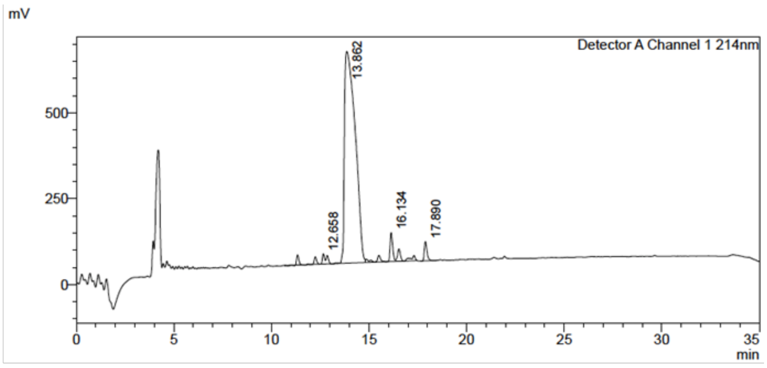

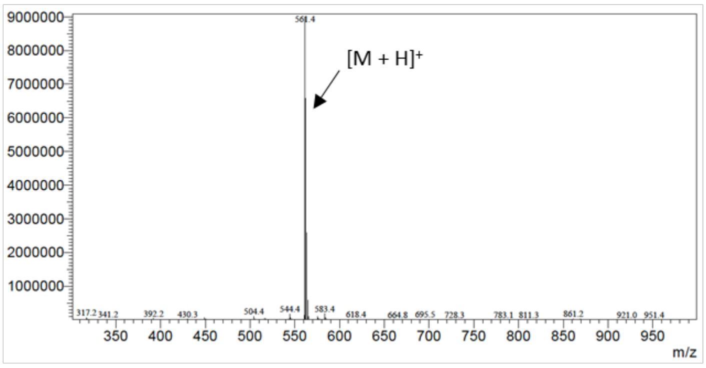

R6
fM-Cha-FG-Pra-NH_2

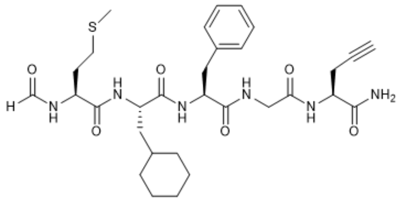

Chemical Formula: $C_{31}H_{44}N_6O_6S$
Exact Mass: 628.30
Molecular Weight: 628.79

Expected MW – 628.3 Da;
Found m/z – 628.5 Da.

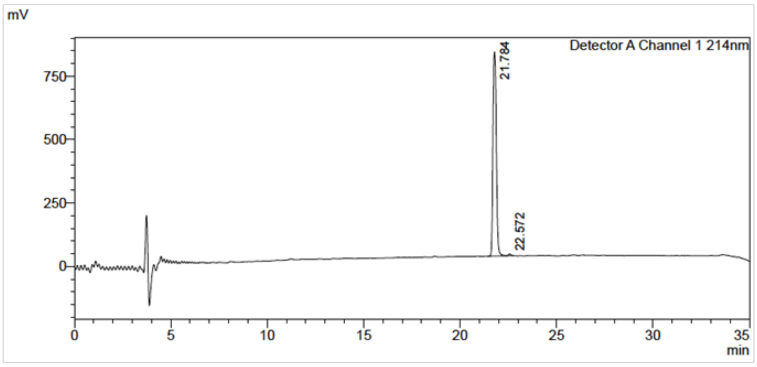

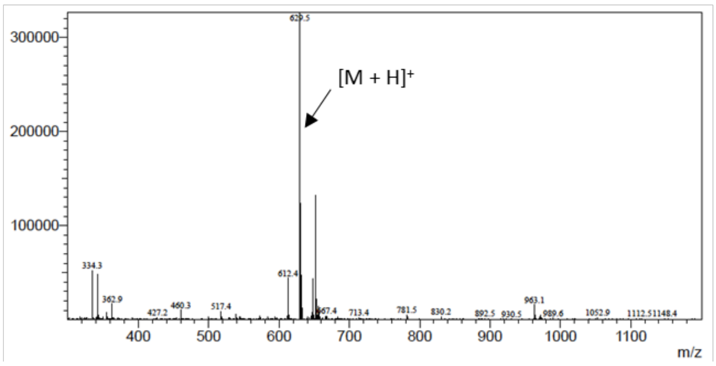

Supplementary Figure 13. Characterisation of fPeptide precursors- continued
Structure, and LC and MS profiles for each of the fPeptide precursors (**R1-13**) used in this study.

R7
f-Nle-LFG-Pra-NH_2

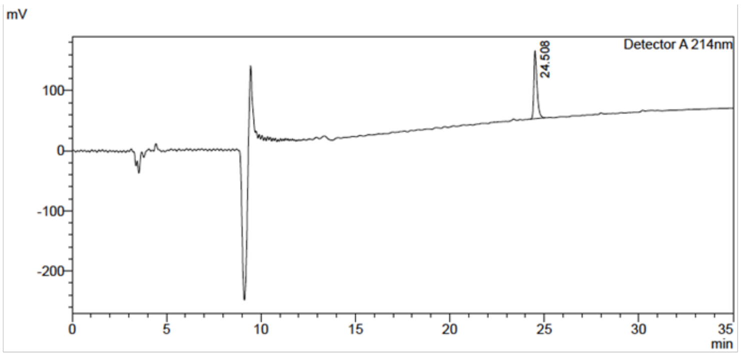

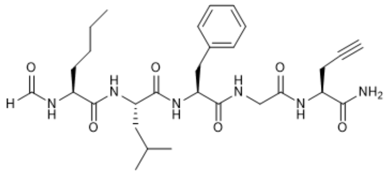

Chemical Formula: $C_{29}H_{42}N_6O_6$
Exact Mass: 570.32
Molecular Weight: 570.69

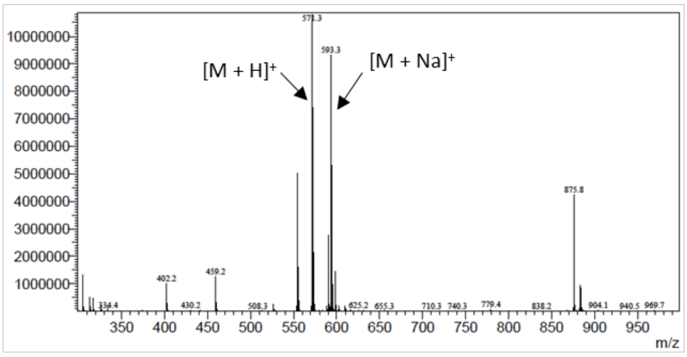

Expected MW – 570.3 Da;
Found m/z – 570.3 Da.

R8
fM-Nle-FG-Pra-NH_2

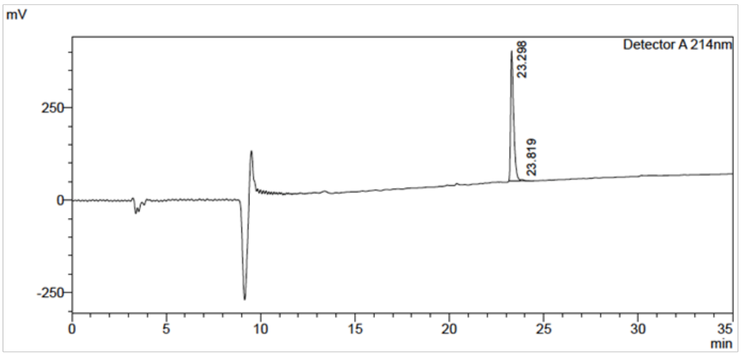

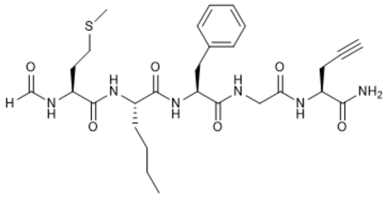

Chemical Formula: $C_{28}H_{40}N_6O_6S$
Exact Mass: 588.27
Molecular Weight: 588.72

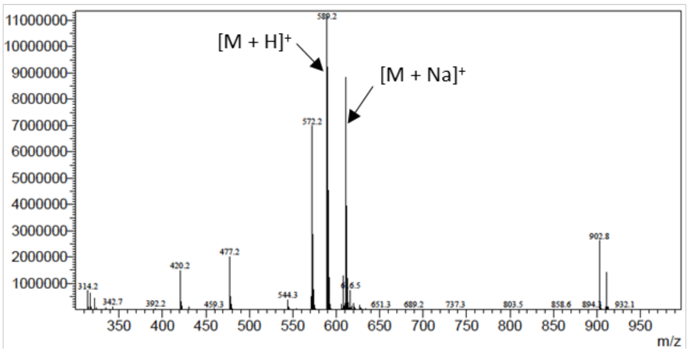

Expected MW – 588.3 Da;
Found m/z – 588.2 Da.

Supplementary Figure 13. Characterisation of fPeptide precursors- continued
Structure, and LC and MS profiles for each of the fPeptide precursors (**R1-13**) used in this study.

R9
fML-Phe(4-F)-G-Pra-NH_2

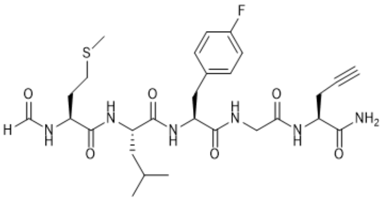

Chemical Formula: $C_{28}H_{39}FN_6O_6S$
Exact Mass: 606.26
Molecular Weight: 606.71

Expected MW – 606.3 Da;
Found m/z – 606.7 Da.

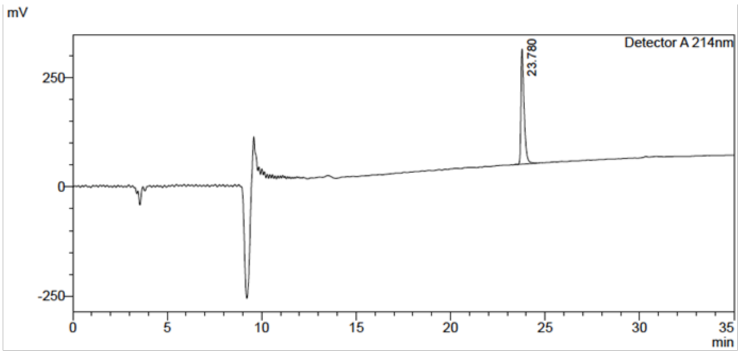

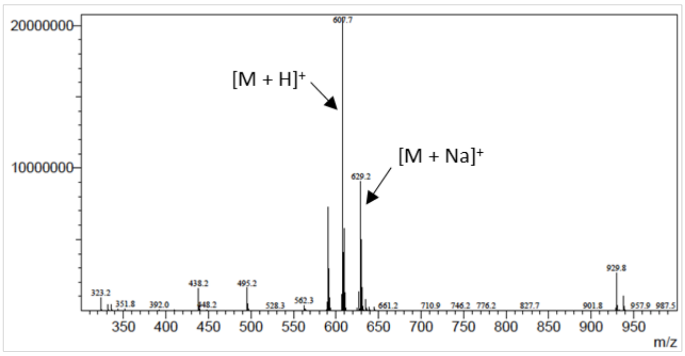

R10
fMLF-Pro-Pra-NH_2

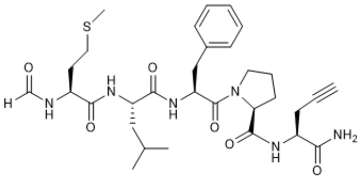

Chemical Formula: $C_{31}H_{44}N_6O_6S$
Exact Mass: 628.30
Molecular Weight: 628.79

Expected MW – 628.3 Da;
Found m/z – 628.3 Da.

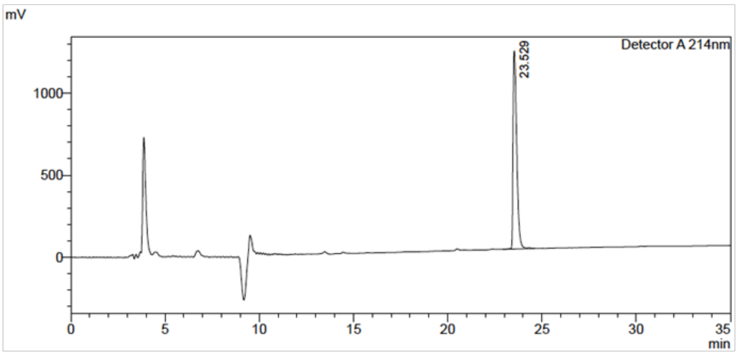

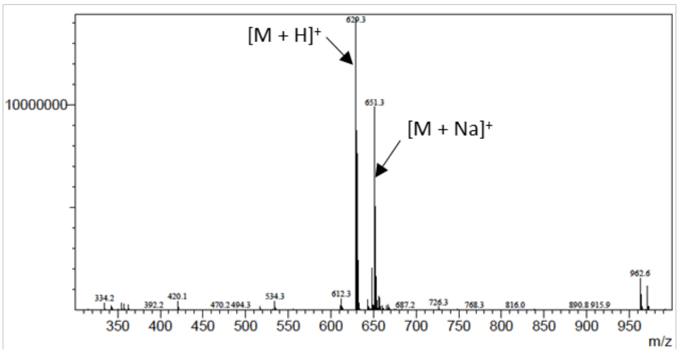

Supplementary Figure 13. Characterisation of fPeptide precursors- continued
Structure, and LC and MS profiles for each of the fPeptide precursors (**R1-13**) used in this study.

R11
fMLF-Lys-Pra-NH_2

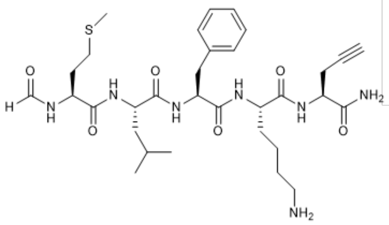

Chemical Formula: $C_{32}H_{49}N_7O_6S$
Exact Mass: 659.35
Molecular Weight: 659.85

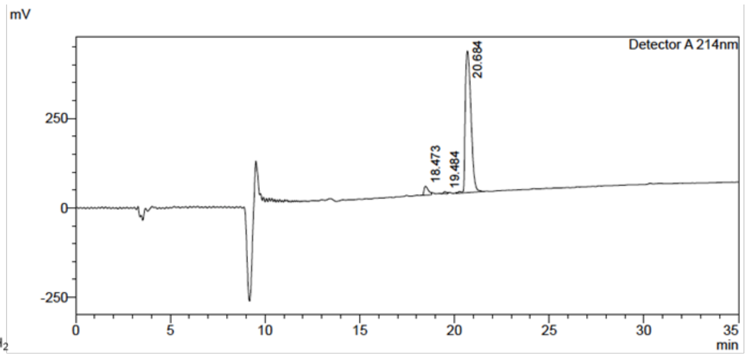

Expected MW – 659.4 Da;
Found m/z – 659.4 Da.

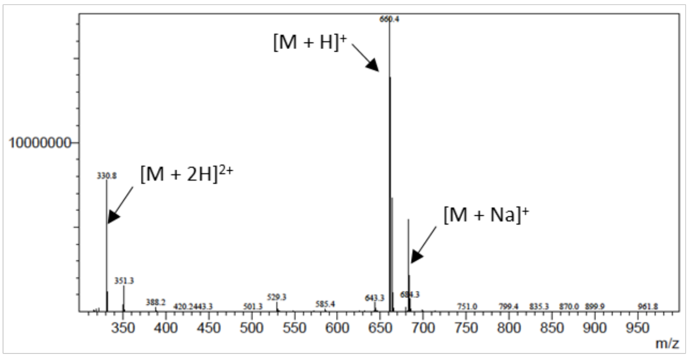

R12
fMLF-Leu-Pra-NH_2

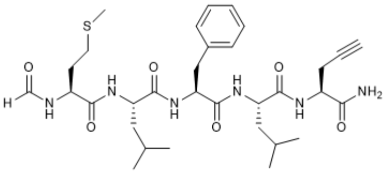

Chemical Formula: $C_{32}H_{48}N_6O_6S$
Exact Mass: 644.34
Molecular Weight: 644.83

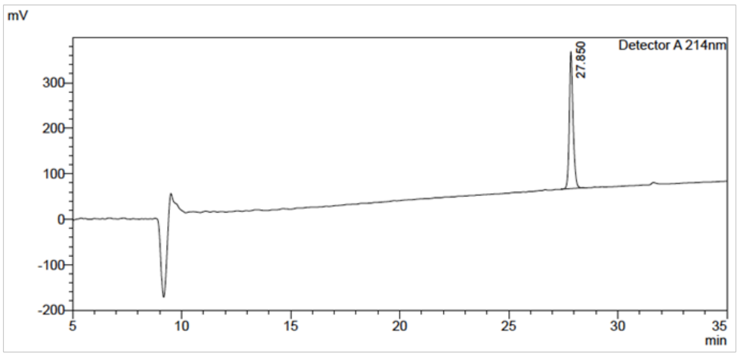

Expected MW – 644.3 Da;
Found m/z – 644.2 Da.

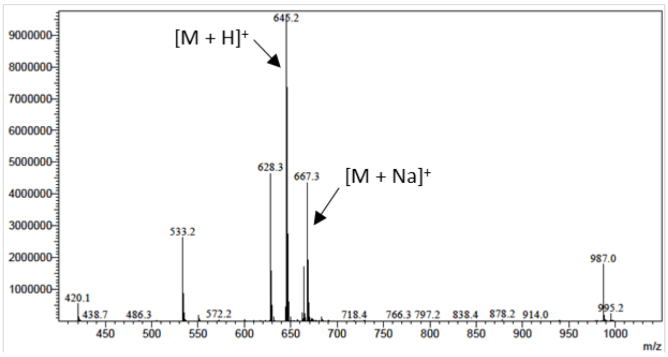

Supplementary Figure 13. Characterisation of fPeptide precursors- continued
Structure, and LC and MS profiles for each of the fPeptide precursors (**R1-13**) used in this study.

R13
fML-Tyr-G-Pra-NH_2

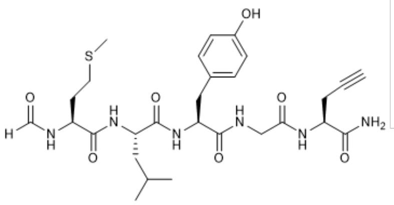

Chemical Formula: $C_{28}H_{40}N_6O_7S$
Exact Mass: 604.27
Molecular Weight: 604.72

Expected MW – 604.3 Da;
Found m/z – 604.4 Da.

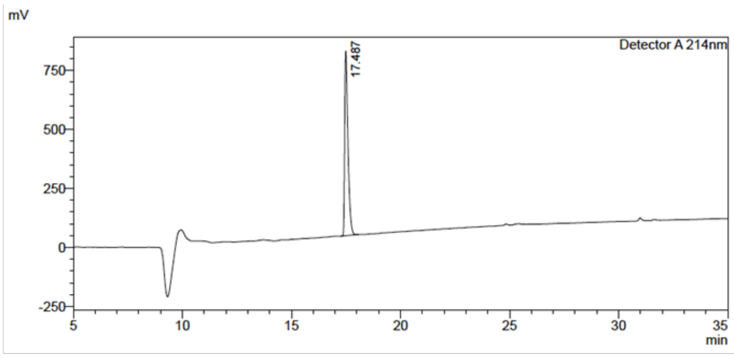

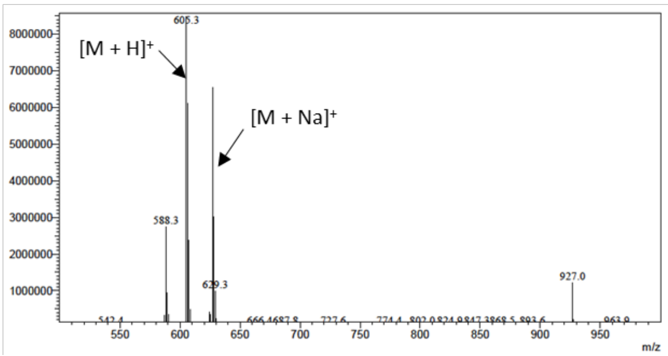

Supplementary Figure 13. Characterisation of fPeptide precursors- continued
Structure, and LC and MS profiles for each of the fPeptide precursors (**R1-13**) used in this study.

C1
Van-C(fMLFG-Tz)

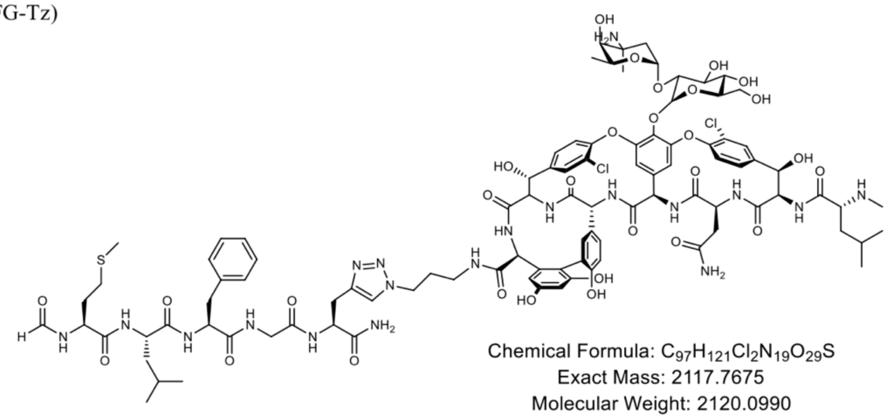

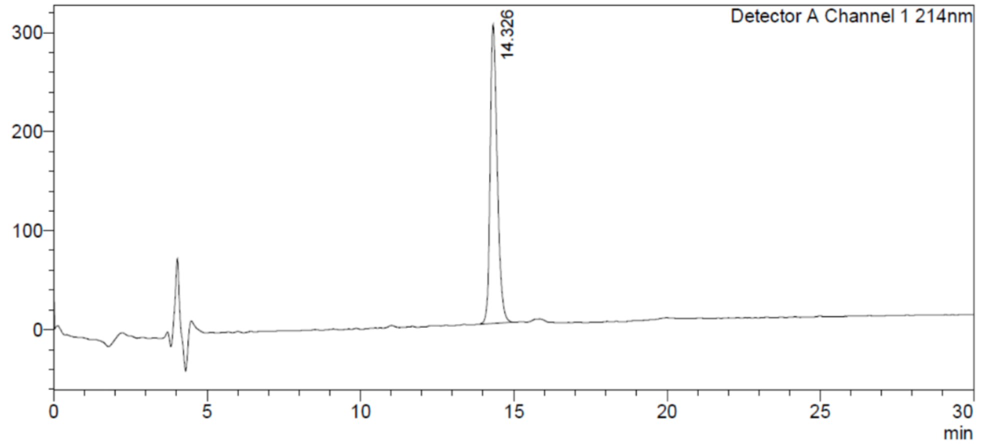

RT = 14.3 min in a gradient of 15-45% ACN in 30 minutes

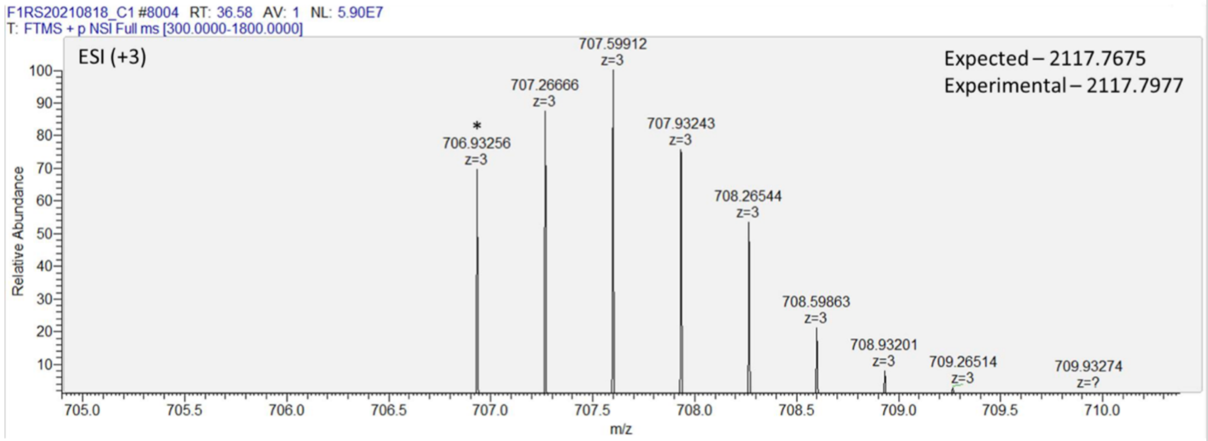

Supplementary Figure 14. Characterisation of fPeptide linked to vancomycin conjugates - continued
Structure, and LC and MS profiles for each of the conjugates (**C1-19)** used in this study.

C2
Van-C(fMLFG-PEG3-Tz)

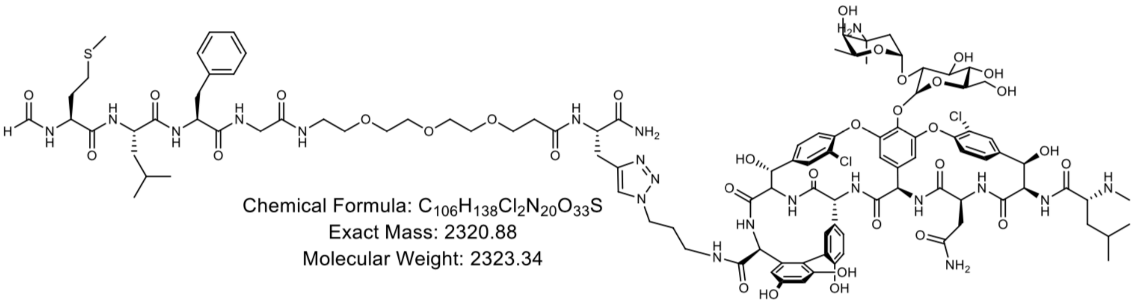

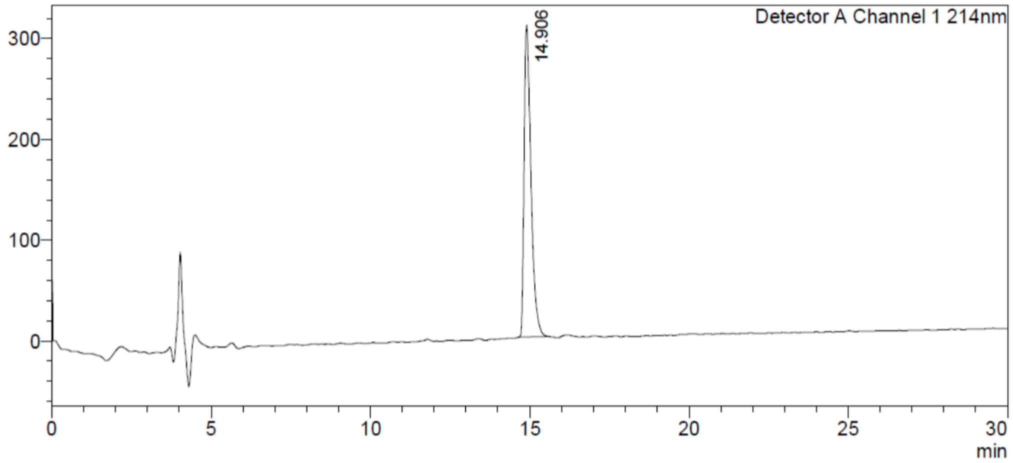

RT = 14.9 min in a gradient of 15-45% ACN in 30 minutes

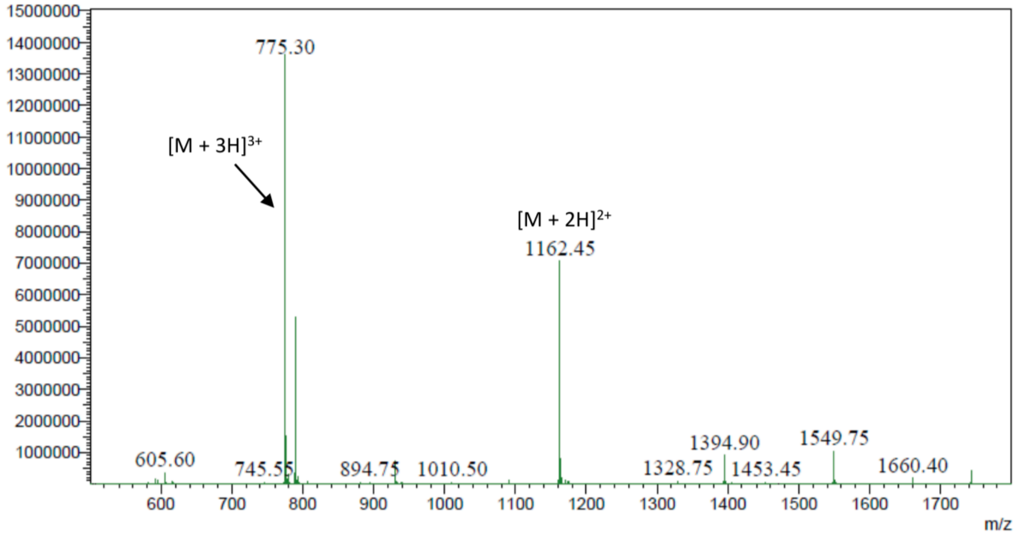

Expected MW – 2320.9 Da;
Found m/z – 2322.9 Da.

Supplementary Figure 14. Characterisation of fPeptide linked to vancomycin conjugates - continued
Structure, and LC and MS profiles for each of the conjugates (**C1-19**) used in this study.

C3

Van-C(fMLFG-(PEG3)2-Tz)

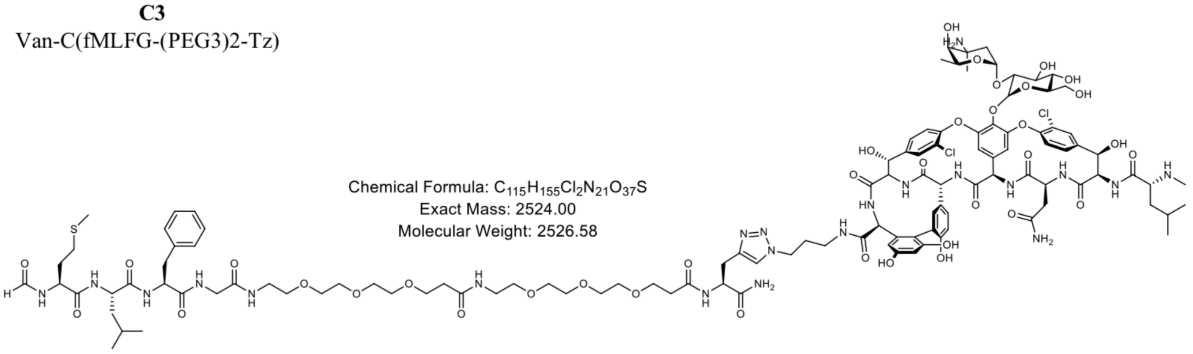

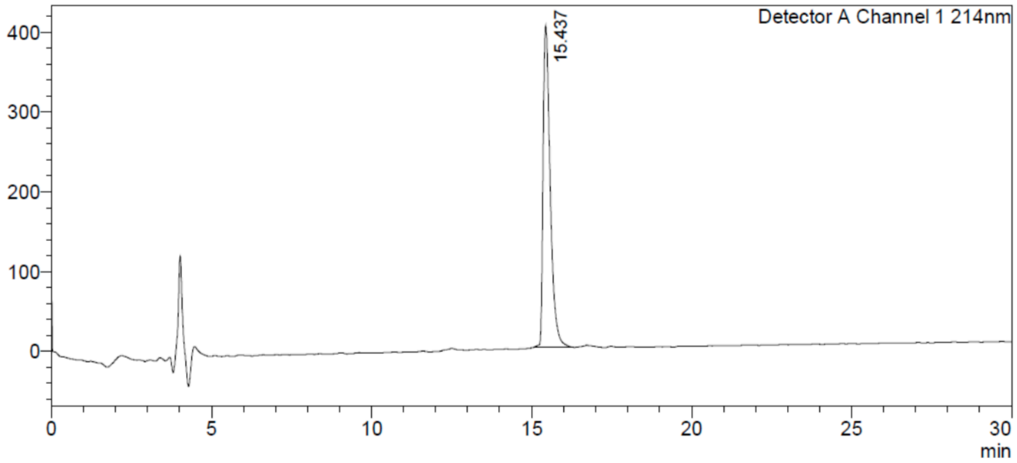

RT = 15.4 min in a gradient of 15-45% ACN in 30 minutes

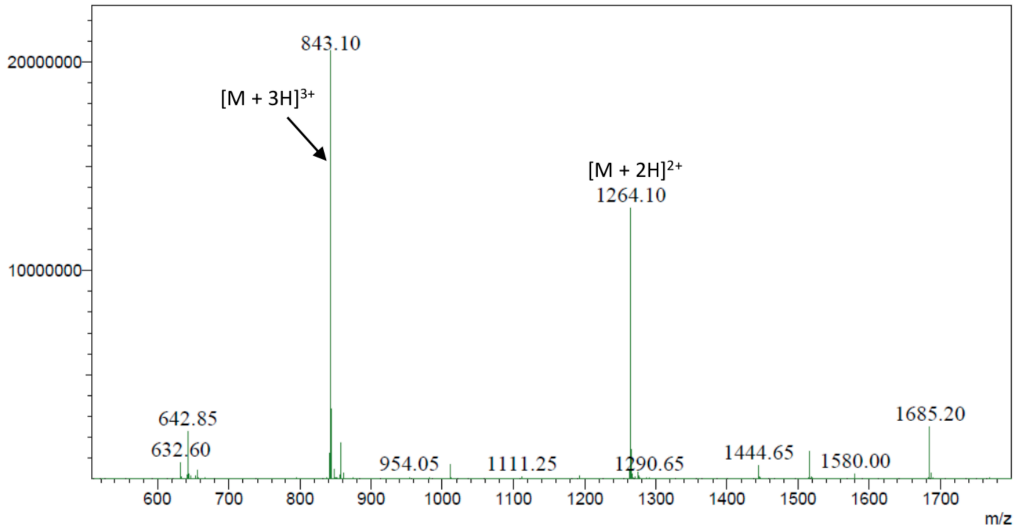

Expected MW – 2524.0 Da;

Found m/z – 2526.2 Da.

Supplementary Figure 14. Characterisation of fPeptide linked to vancomycin conjugates - continued

Structure, and LC and MS profiles for each of the conjugates (**C1-19)** used in this study.

N4
Van-N(fMLFG-Tz)

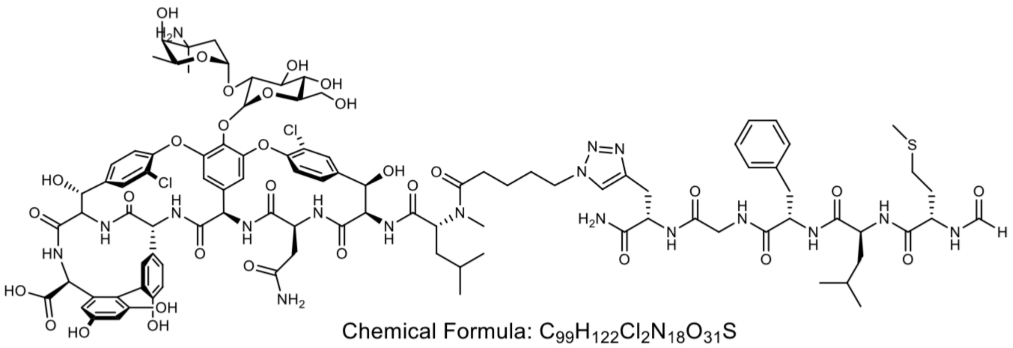

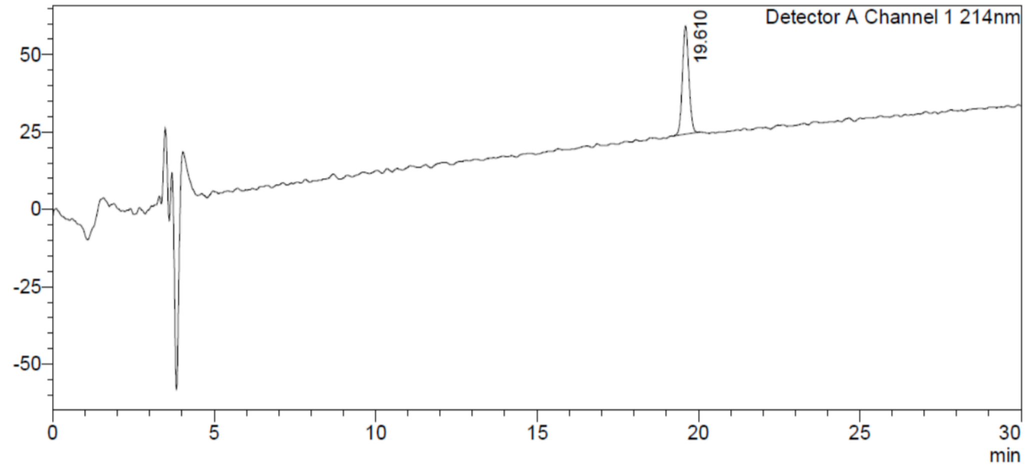

RT = 19.6 min in a gradient of 15-45% ACN in 30 minutes

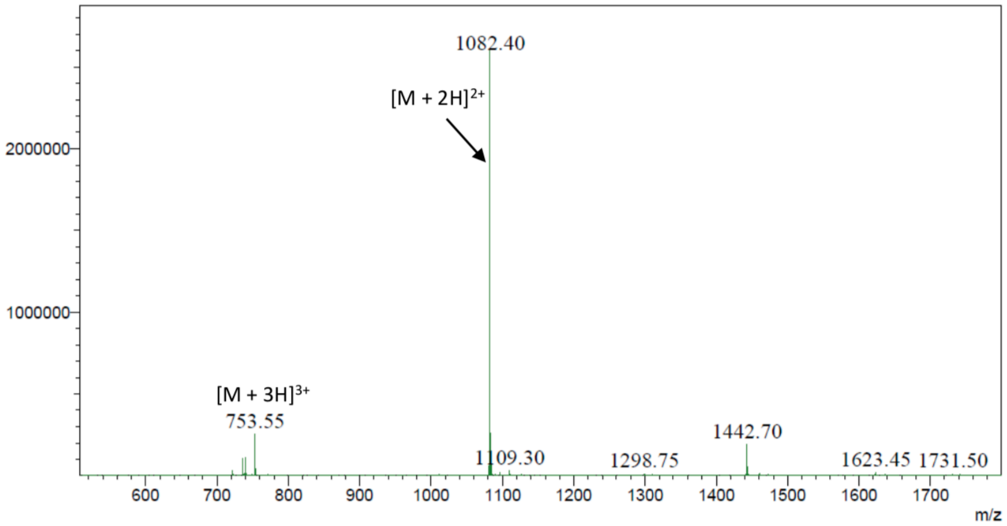

Expected MW – 2160.8 Da;
Found m/z – 2162.8 Da.

Supplementary Figure 14. Characterisation of fPeptide linked to vancomycin conjugates - continued
Structure, and LC and MS profiles for each of the conjugates (**C1-19)** used in this study.

N5
Van-N(fMLFG-PEG3-Tz)

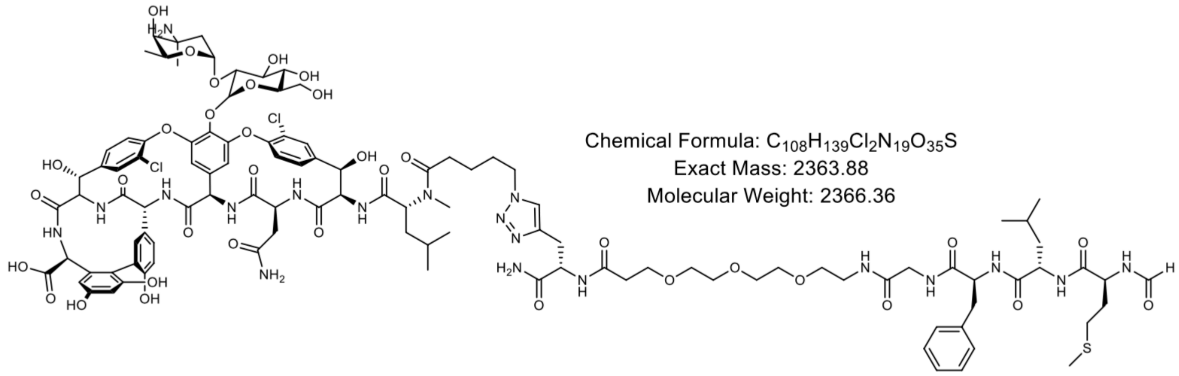

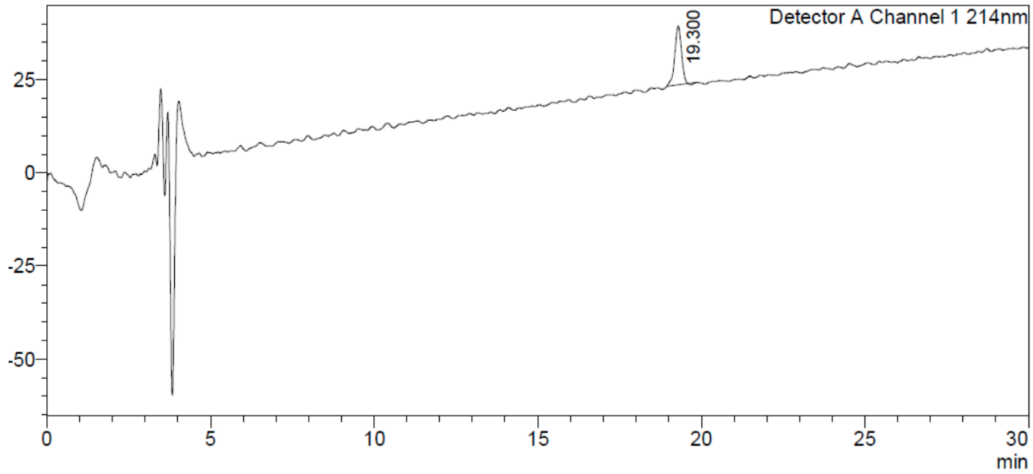

RT = 19.3 min in a gradient of 15-45% ACN in 30 minutes

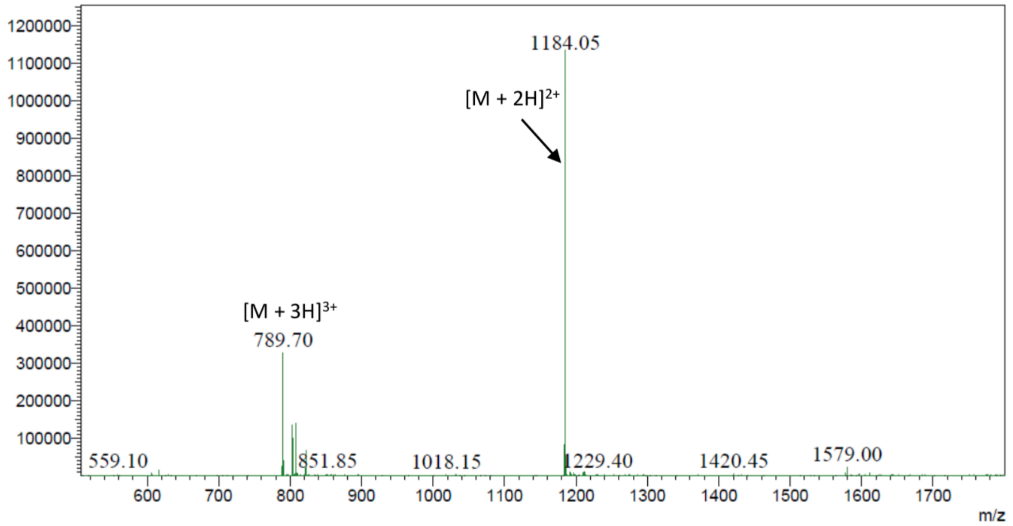

Expected MW – 2363.9 Da;
Found m/z – 2366.1 Da.

Supplementary Figure 14. Characterisation of fPeptide linked to vancomycin conjugates - continued
Structure, and LC and MS profiles for each of the conjugates (**C1-19**) used in this study.

N6

Van-N(fMLFG-(PEG3)2-Tz)

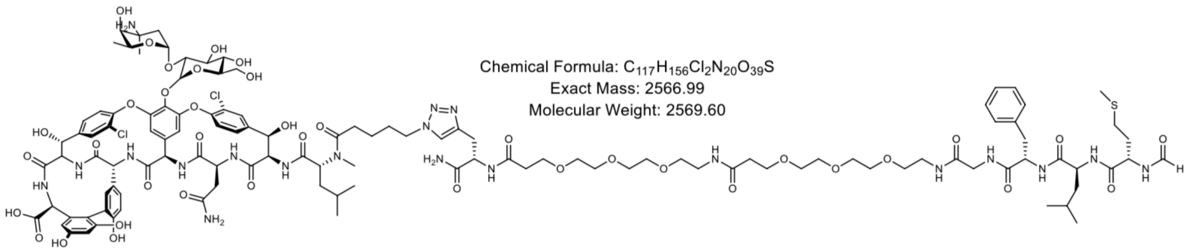

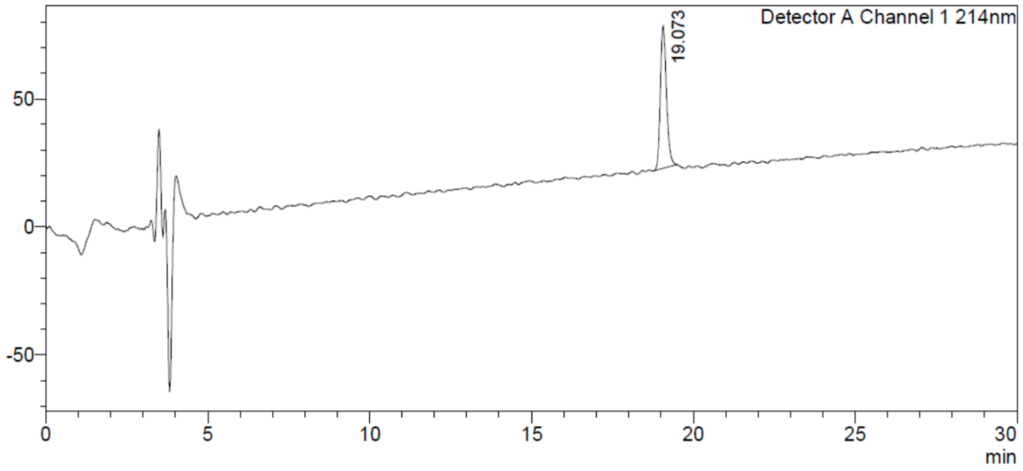

RT = 19.1 min in a gradient of 15-45% ACN in 30 minutes

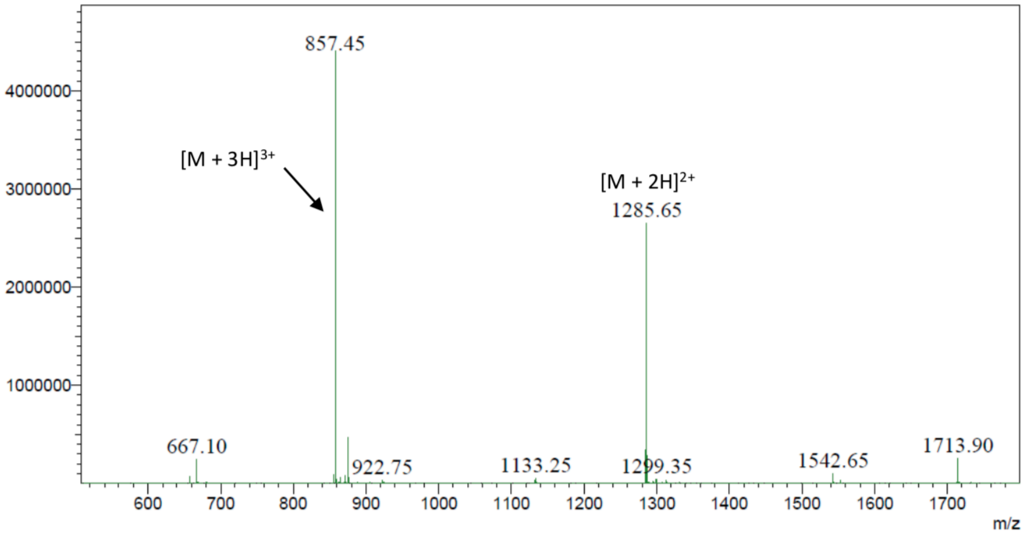

Expected MW – 2566.9 Da;
Found m/z – 2569.3 Da.

Supplementary Figure 14. Characterisation of fPeptide linked to vancomycin conjugates - continued

Structure, and LC and MS profiles for each of the conjugates (**C1-19)** used in this study.

V7
Van-V(fMLFG-Tz)

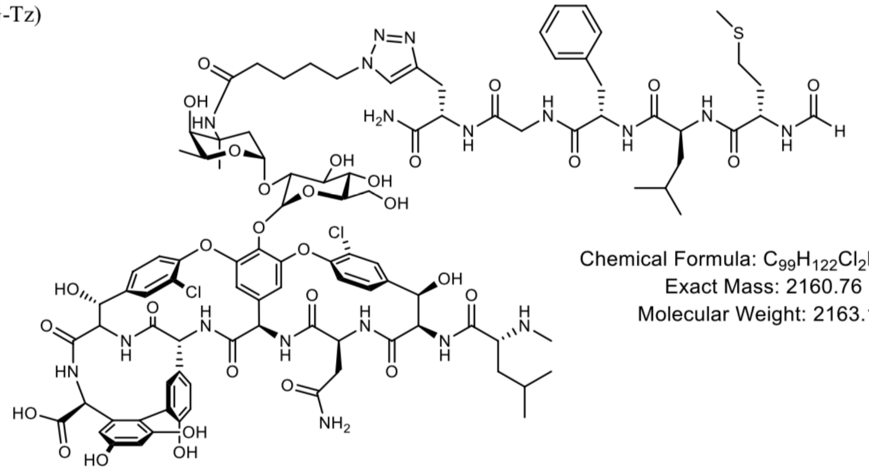

Chemical Formula: $C_{99}H_{122}Cl_2N_{18}O_{31}S$
Exact Mass: 2160.76
Molecular Weight: 2163.12

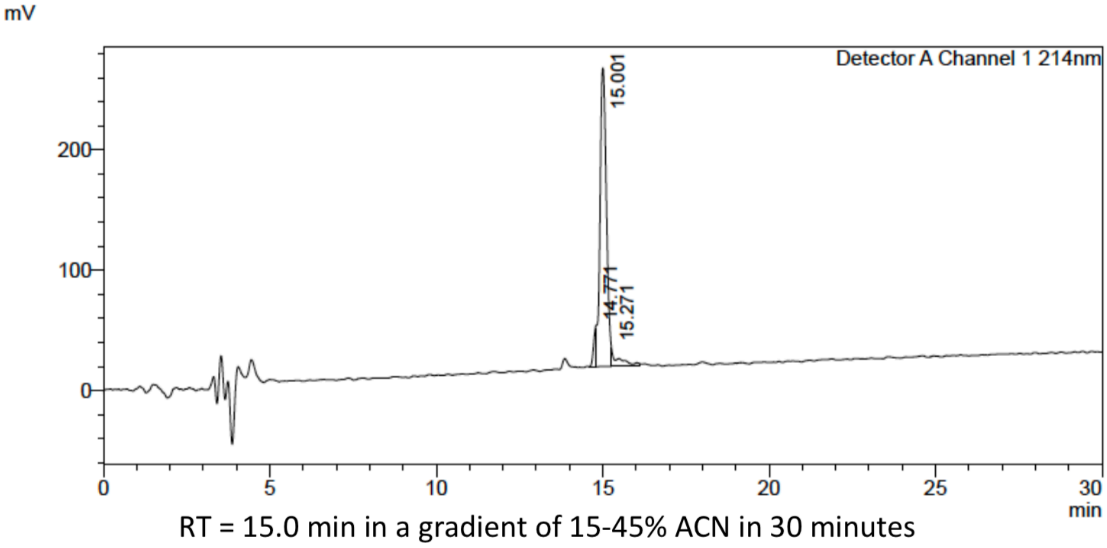

RT = 15.0 min in a gradient of 15-45% ACN in 30 minutes

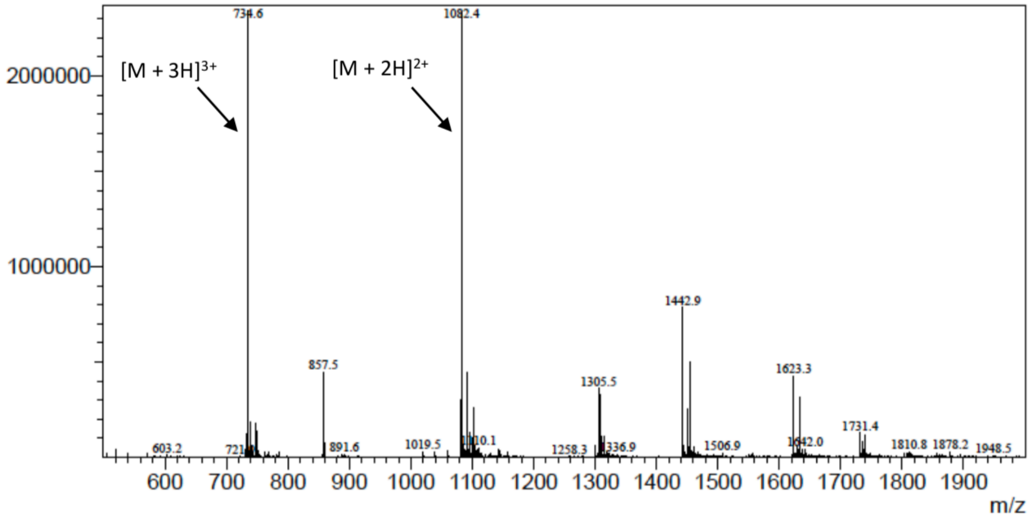

Expected MW – 2160.8 Da;
Found m/z – 2162.8 Da.

Supplementary Figure 14. Characterisation of fPeptide linked to vancomycin conjugates - continued

Structure, and LC and MS profiles for each of the conjugates (**C1-19)** used in this study.

V8

Van-V(fMLFG-PEG3-Tz)

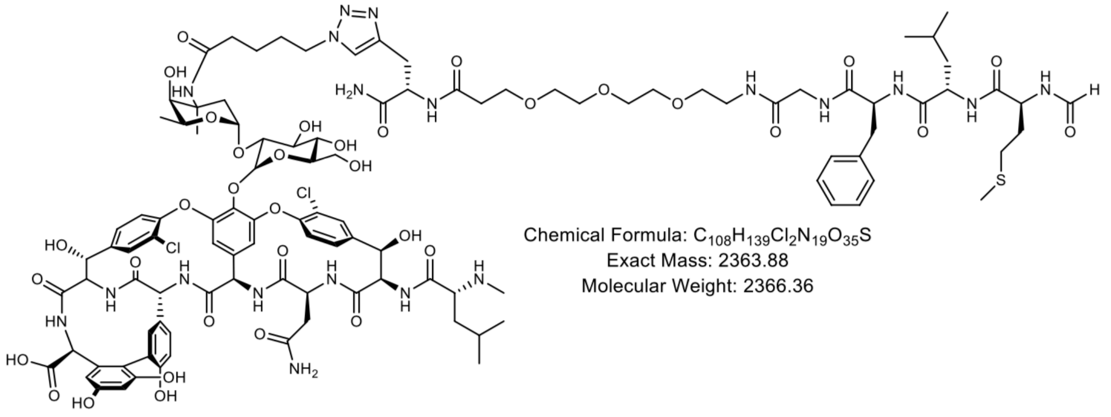

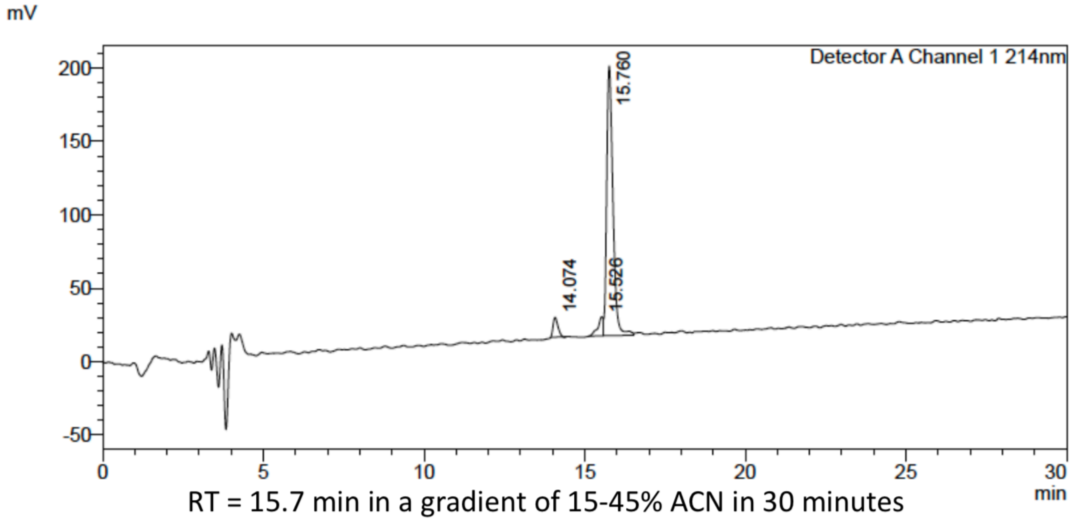

RT = 15.7 min in a gradient of 15-45% ACN in 30 minutes

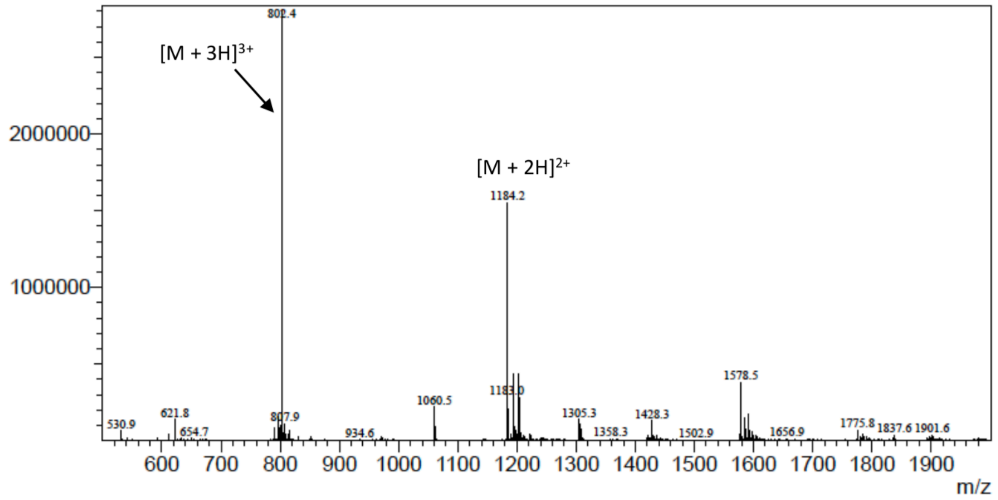

Expected MW – 2363.9 Da;

Found m/z – 2366.4 Da.

Supplementary Figure 14. Characterisation of fPeptide linked to vancomycin conjugates - continued

Structure, and LC and MS profiles for each of the conjugates (**C1-19)** used in this study.

V9

Van-V(fMLFG-(PEG3)2-Tz)

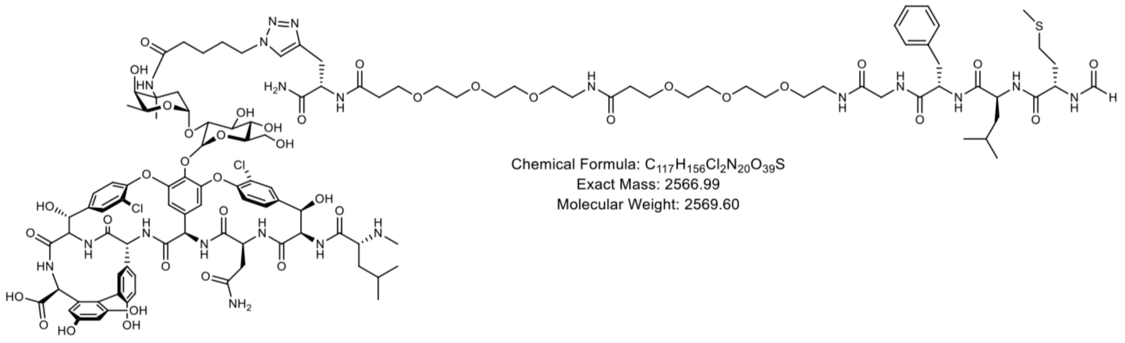

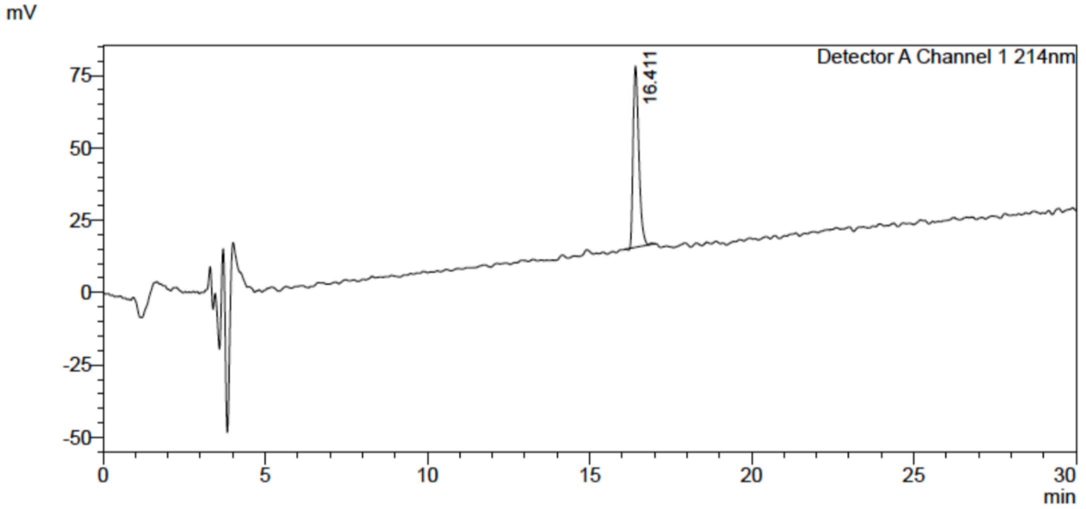

RT = 16.4 min in a gradient of 15-45% ACN in 30 minutes

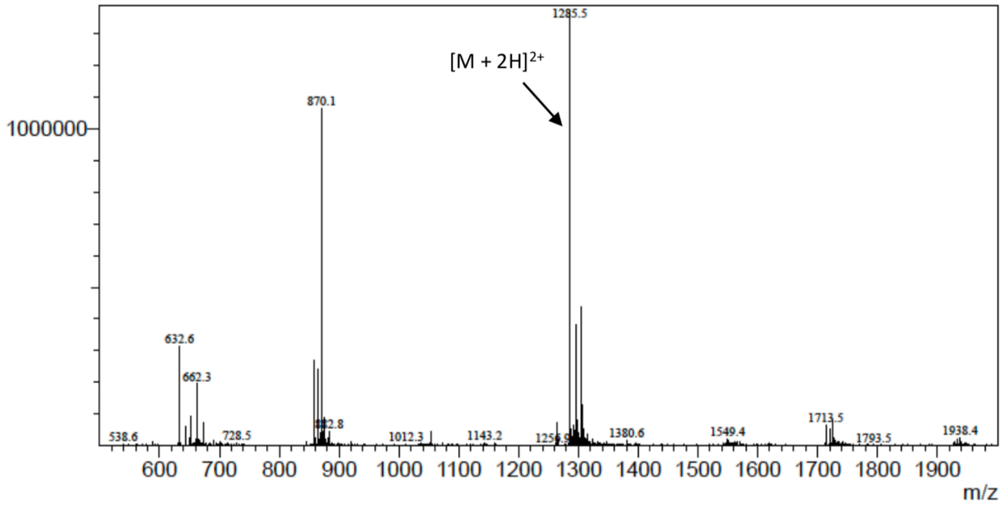

Expected MW – 2566.9 Da;

Found m/z – 2569.0 Da.

Supplementary Figure 14. Characterisation of fPeptide linked to vancomycin conjugates - continued

Structure, and LC and MS profiles for each of the conjugates (**C1-19)** used in this study.

C10

Van-C(fMLFG-PEG4-Tz)

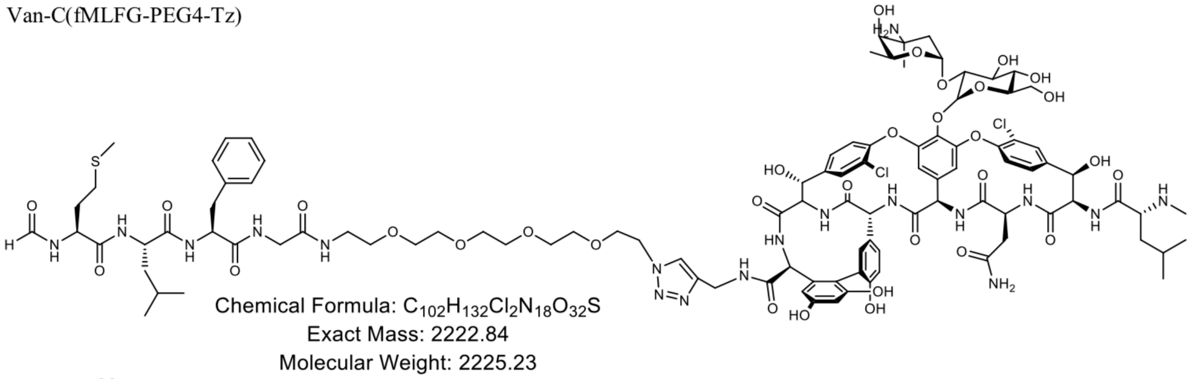

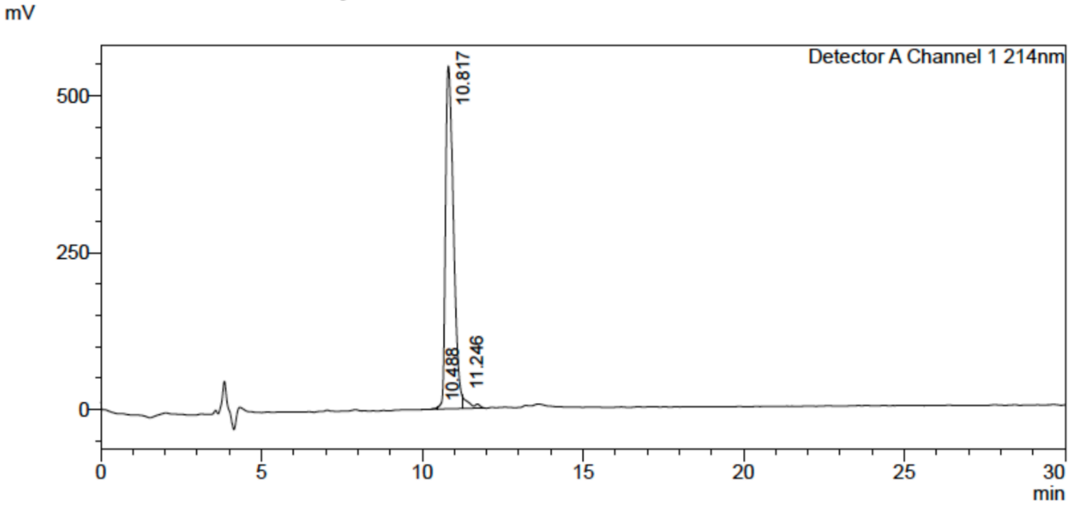

RT = 10.8 min in a gradient of 20-50% ACN in 30 minutes

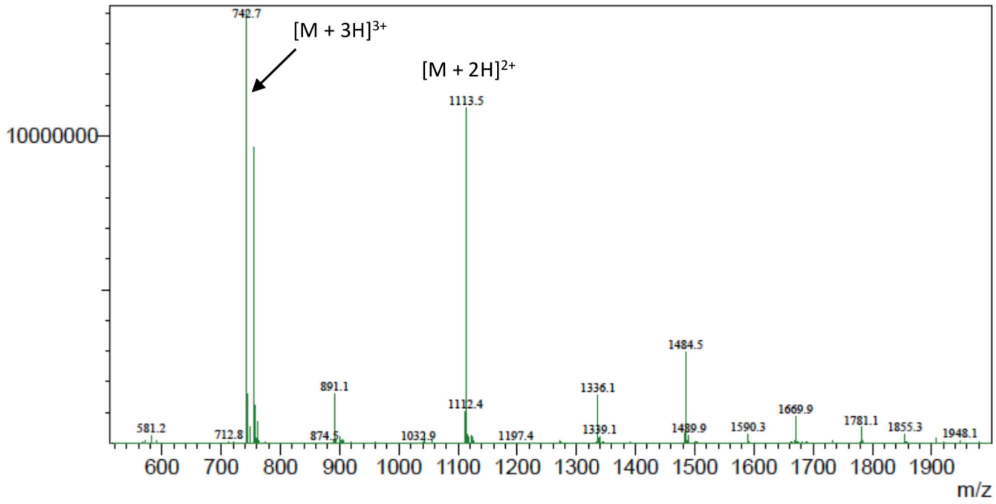

Expected MW – 2222.8 Da;

Found m/z – 2225.0 Da.

Supplementary Figure 14. Characterisation of fPeptide linked to vancomycin conjugates - continued

Structure, and LC and MS profiles for each of the conjugates (**C1-19)** used in this study.

C11

Van-C(fMChaFG-Tz)

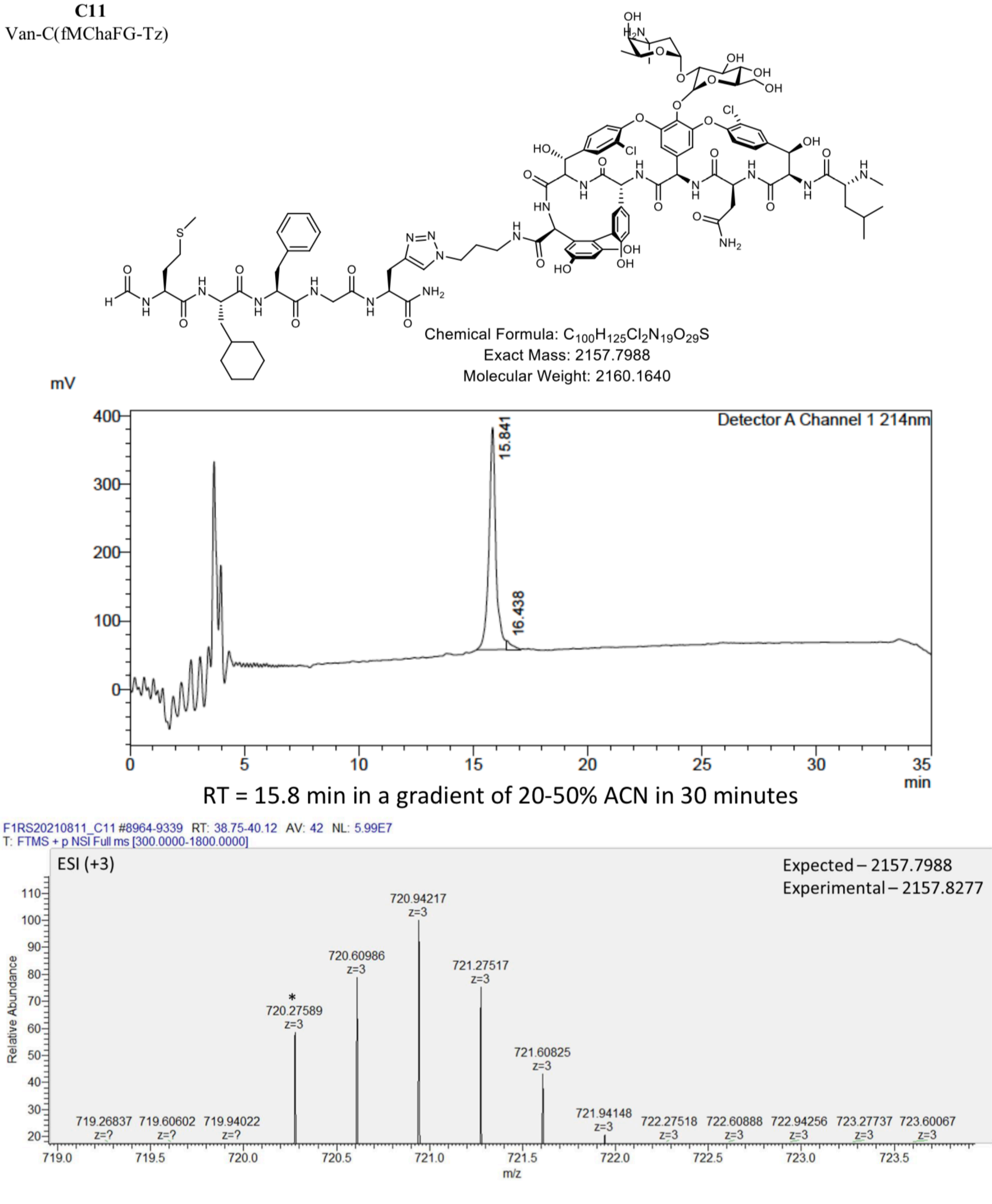

Supplementary Figure 14. Characterisation of fPeptide linked to vancomycin conjugates - continued

Structure, and LC and MS profiles for each of the conjugates (**C1-19)** used in this study.

C12
Van-C(H-MLFG-Tz)

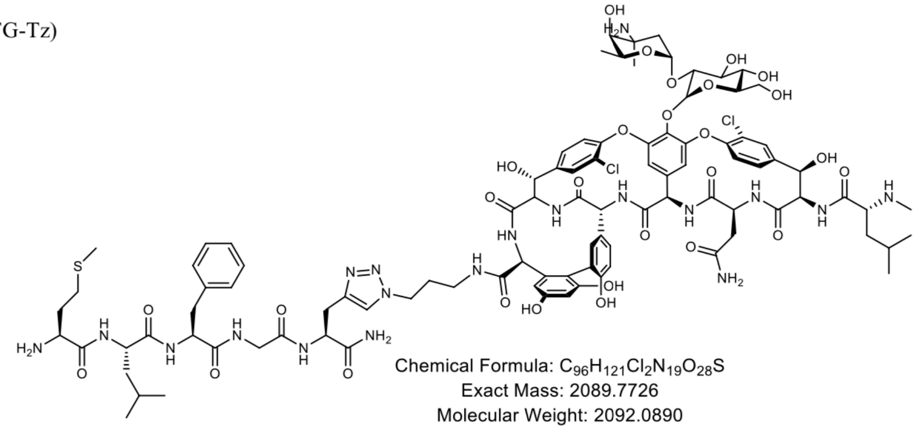

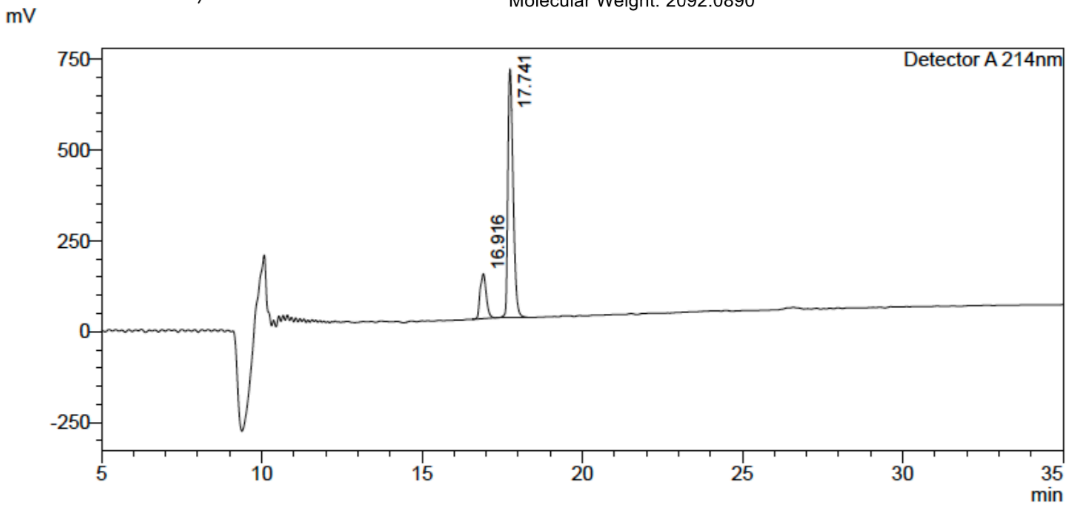

RT = 15.8 min in a gradient of 20-50% ACN in 30 minutes

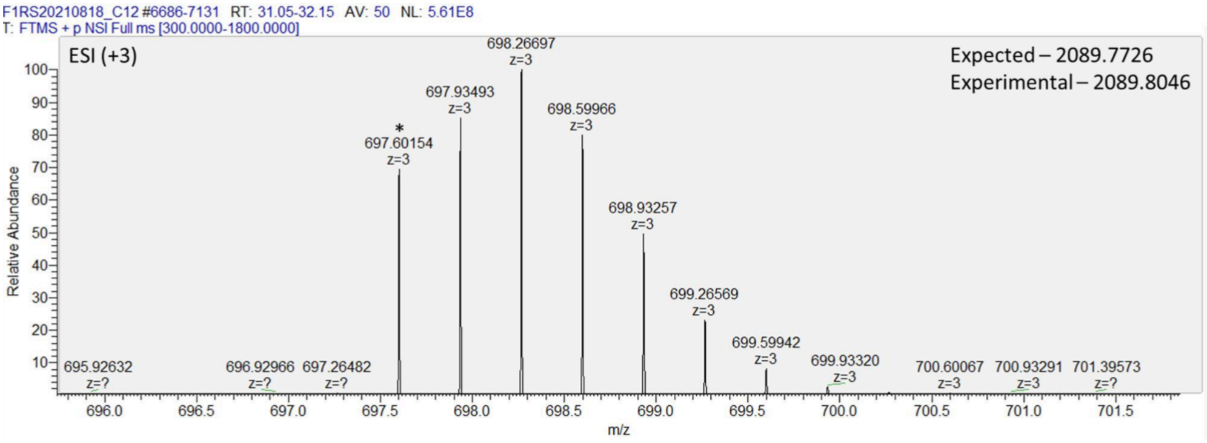

Supplementary Figure 14. Characterisation of fPeptide linked to vancomycin conjugates - continued
Structure, and LC and MS profiles for each of the conjugates (**C1-19)** used in this study.

C13

Van-C(fNleLFG-Tz)

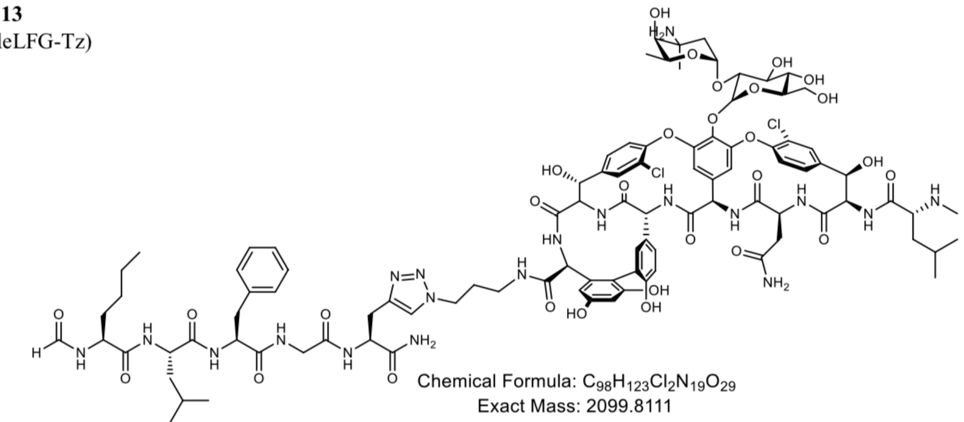

Chemical Formula: $C_{98}H_{123}Cl_2N_{19}O_{29}$

Exact Mass: 2099.8111

Molecular Weight: 2102.0660

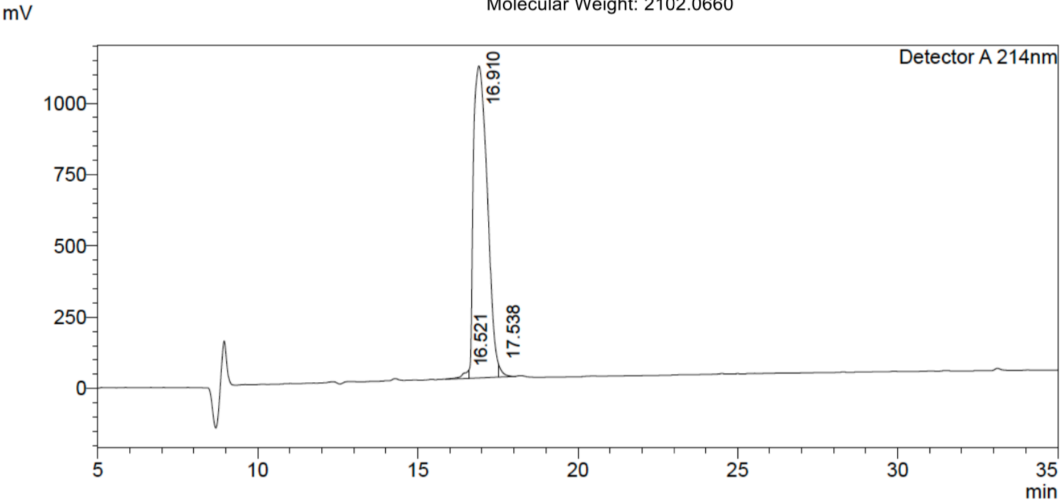

RT = 16.9 min in a gradient of 10-10-70% ACN in 35 minutes

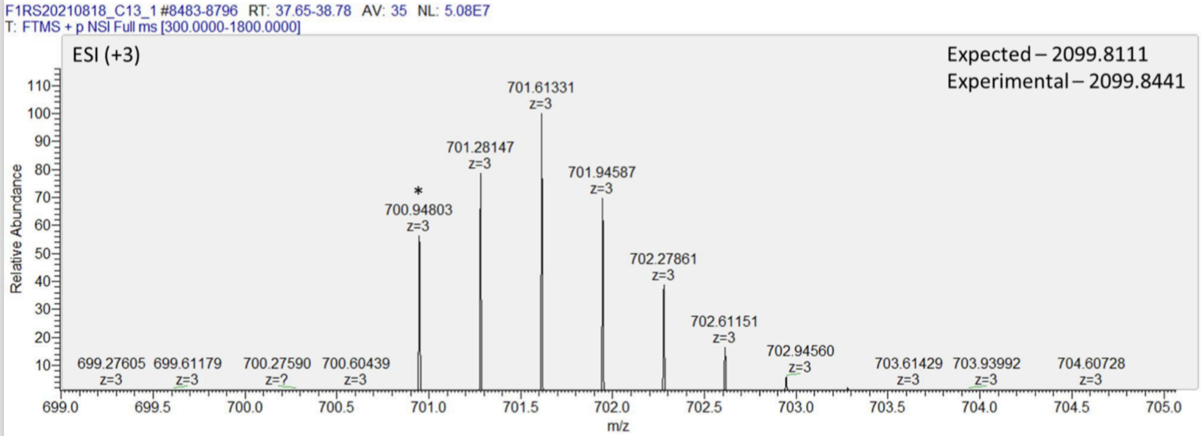

Supplementary Figure 14. Characterisation of fPeptide linked to vancomycin conjugates - continued

Structure, and LC and MS profiles for each of the conjugates (**C1-19**) used in this study.

C14
Van-C(fM-Nle-LFG-Tz)

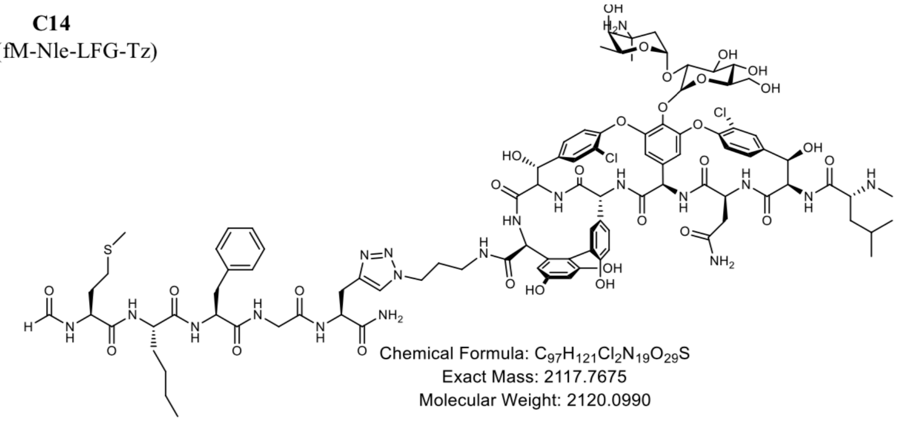

<Chromatogram>

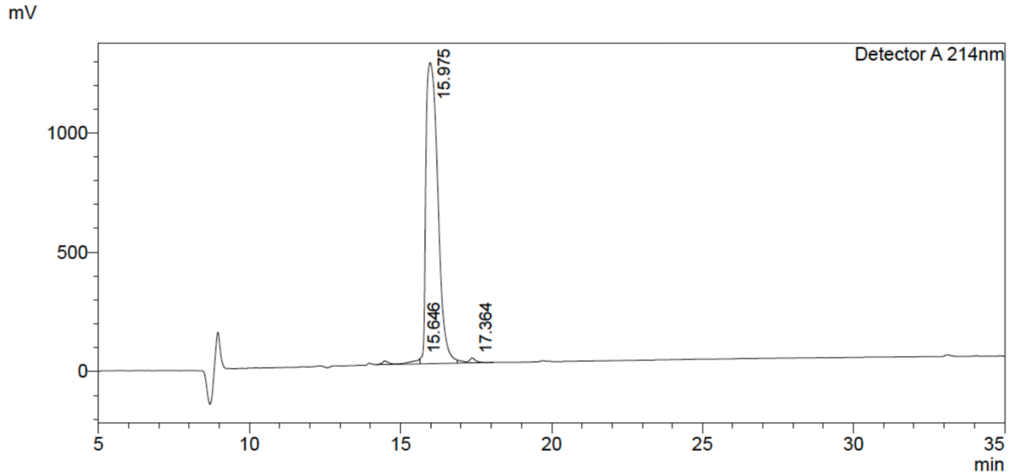

RT = 15.9 min in a gradient of 10-10-70% ACN in 35 minutes

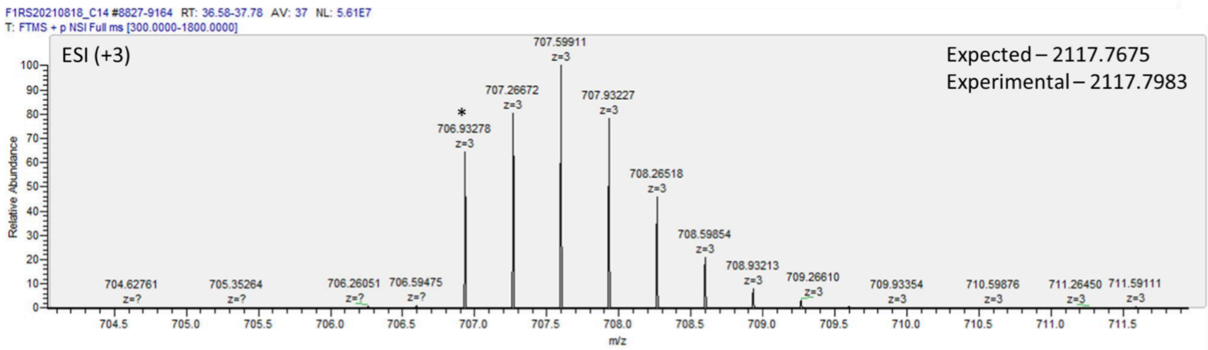

Supplementary Figure 14. Characterisation of fPeptide linked to vancomycin conjugates - continued
Structure, and LC and MS profiles for each of the conjugates (**C1-19)** used in this study.

C15

Van-C(fML-Phe(4F)-G-Tz)

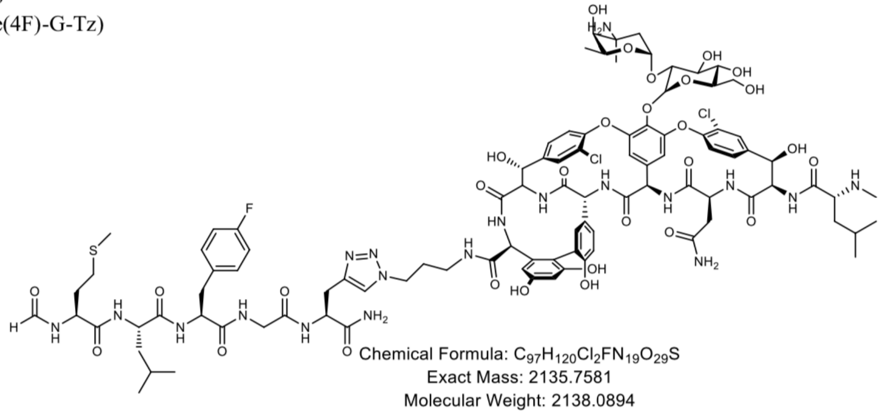

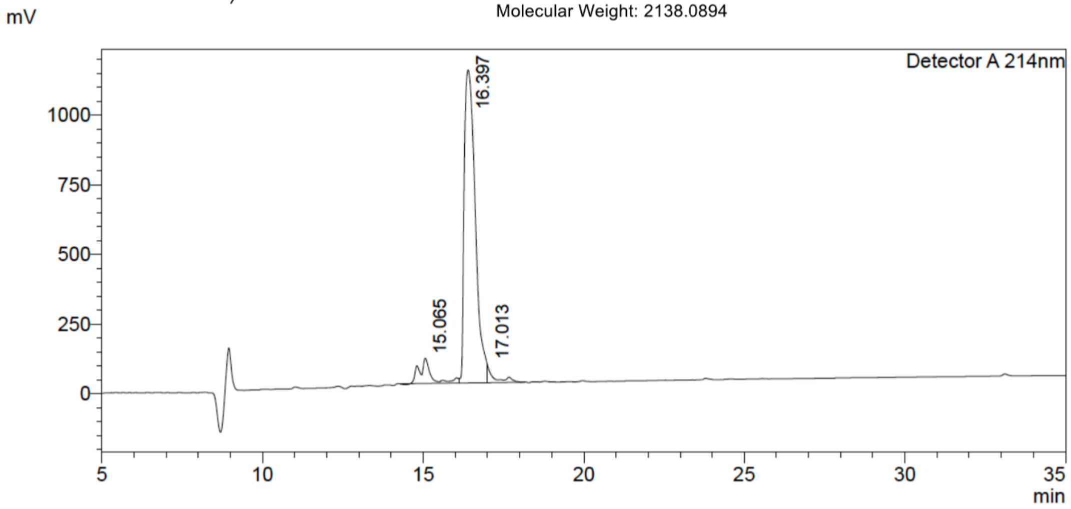

RT = 16.4 min in a gradient of 10-10-70% ACN in 35 minutes

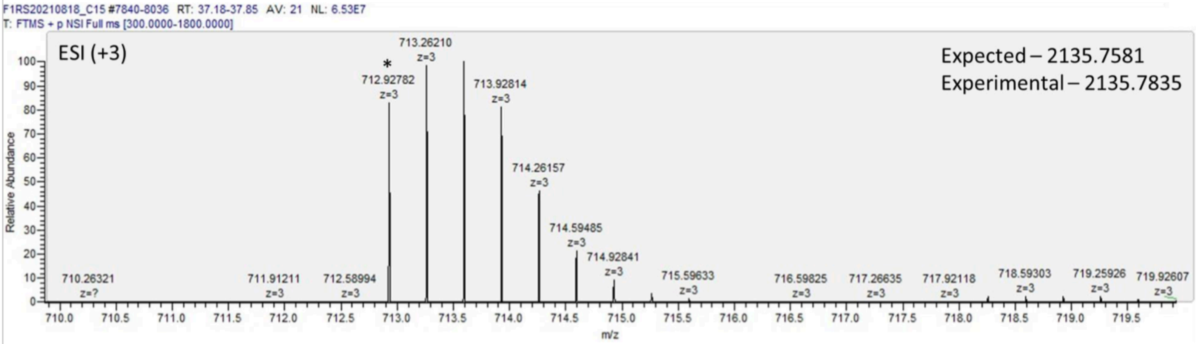

Supplementary Figure 14. Characterisation of fPeptide linked to vancomycin conjugates - continued

Structure, and LC and MS profiles for each of the conjugates (**C1-19)** used in this study.

C16
Van-C(fMLF-Pro-Tz)

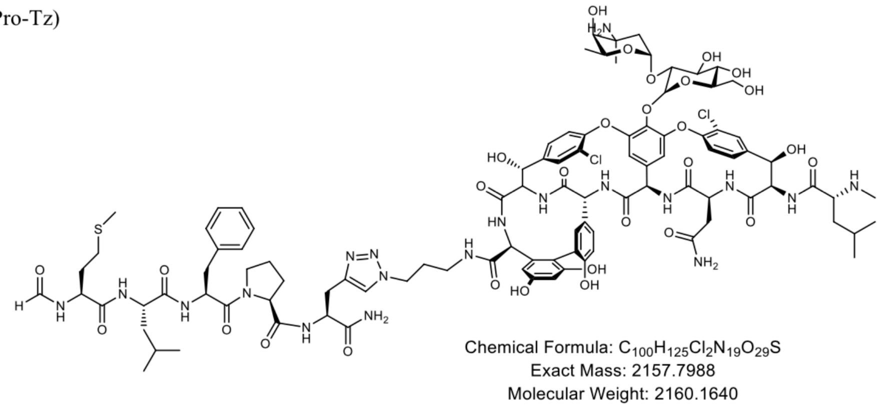

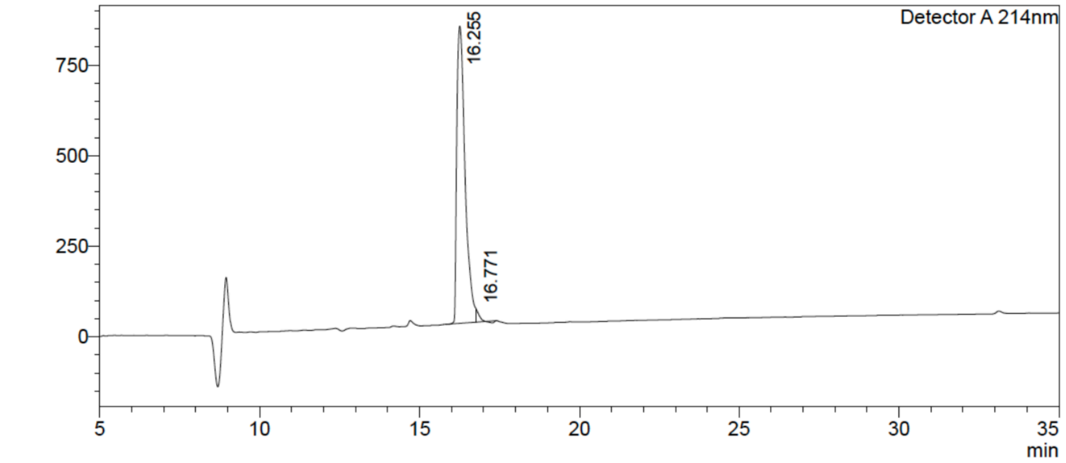

RT = 16.3 min in a gradient of 10-10-70% ACN in 35 minutes

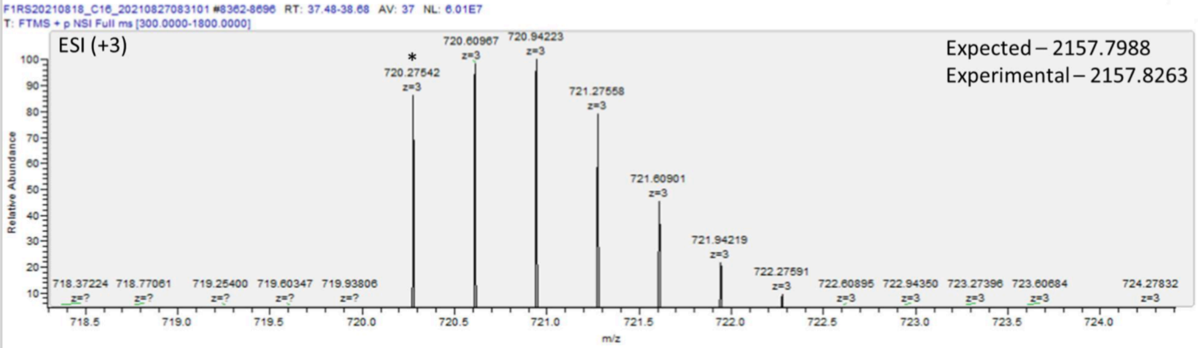

Supplementary Figure 14. Characterisation of fPeptide linked to vancomycin conjugates - continued
Structure, and LC and MS profiles for each of the conjugates (**C1-19**) used in this study.

C17
Van-C(fMLF-Lys-Tz)

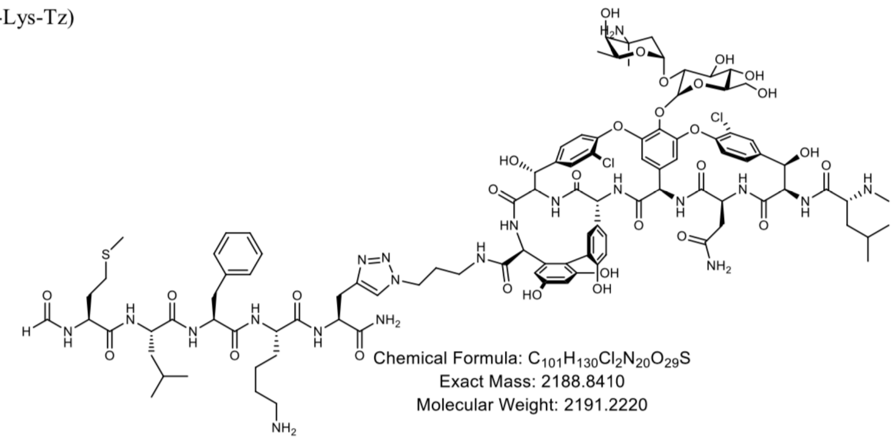

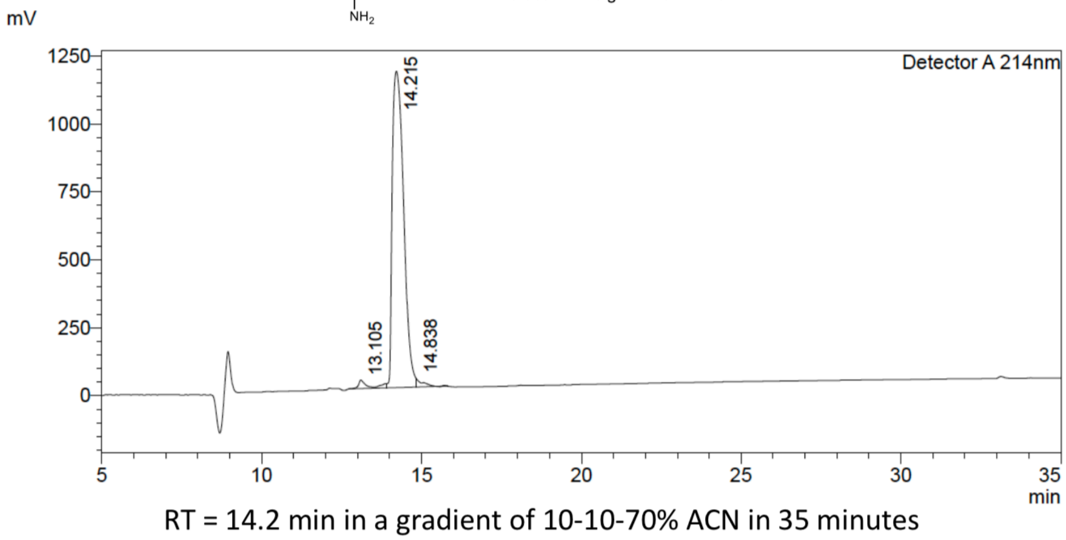

RT = 14.2 min in a gradient of 10-10-70% ACN in 35 minutes

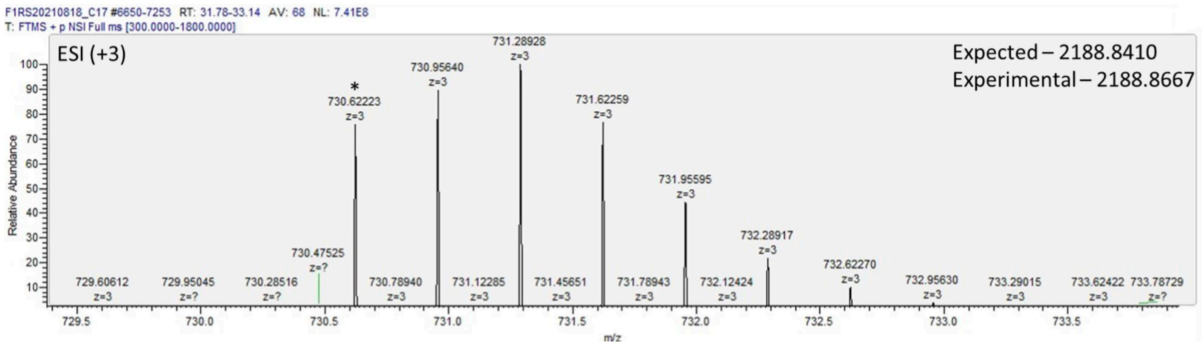

Supplementary Figure 14. Characterisation of fPeptide linked to vancomycin conjugates - continued
Structure, and LC and MS profiles for each of the conjugates (**C1-19)** used in this study.

C18
Van-C(fMLF-Leu-Tz)

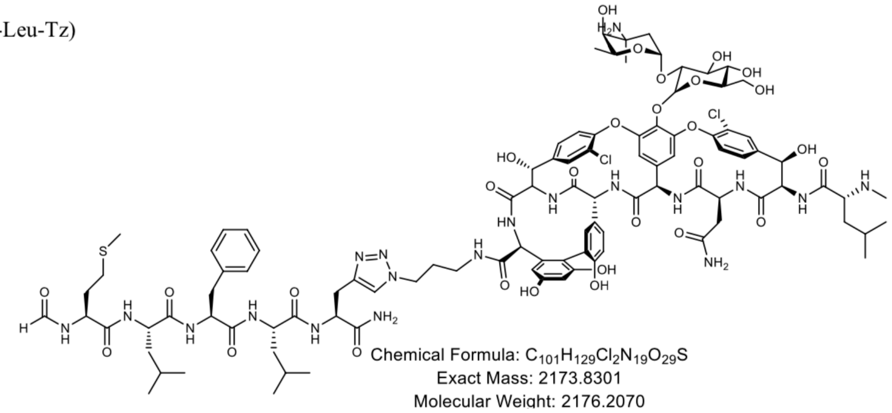

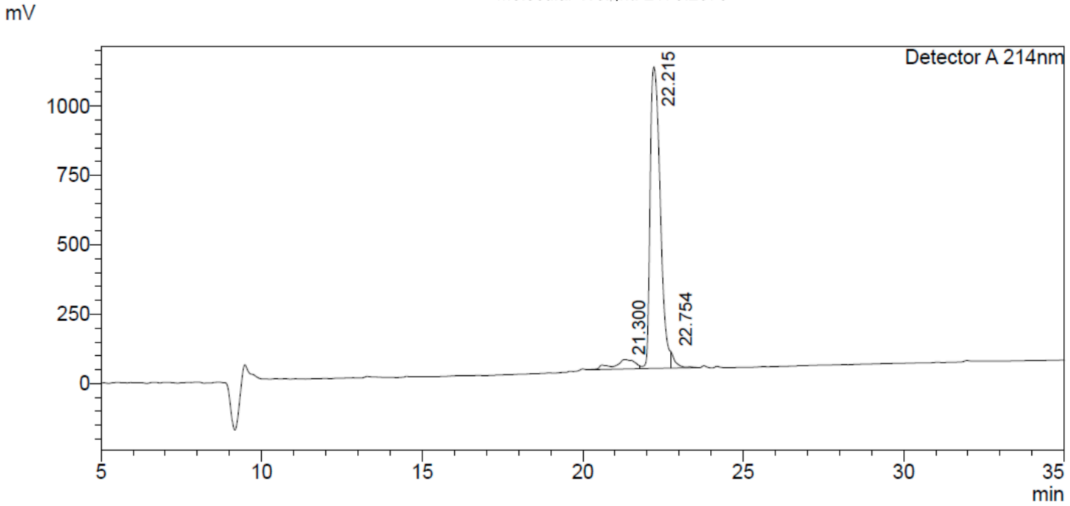

RT = 22.2 min in a gradient of 10-10-70% ACN in 35 minutes

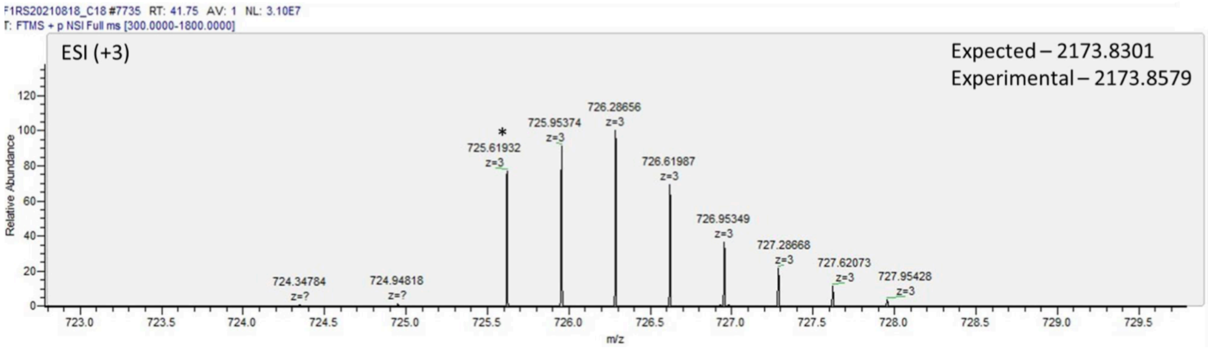

Supplementary Figure 14. Characterisation of fPeptide linked to vancomycin conjugates - continued
Structure, and LC and MS profiles for each of the conjugates (**C1-19)** used in this study.

C19
Van-C(fML-Tyr-G-Tz)

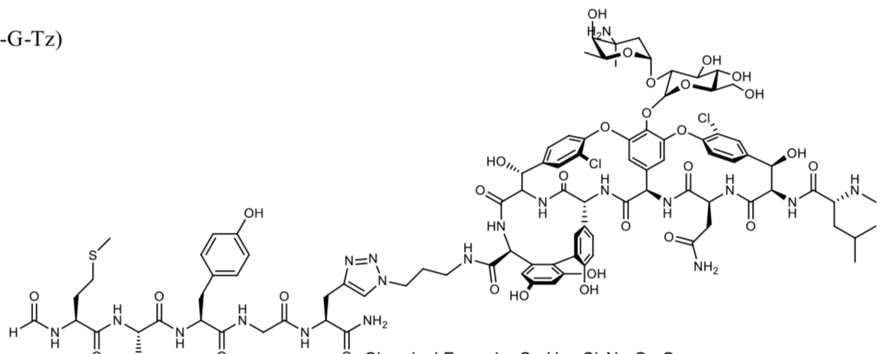

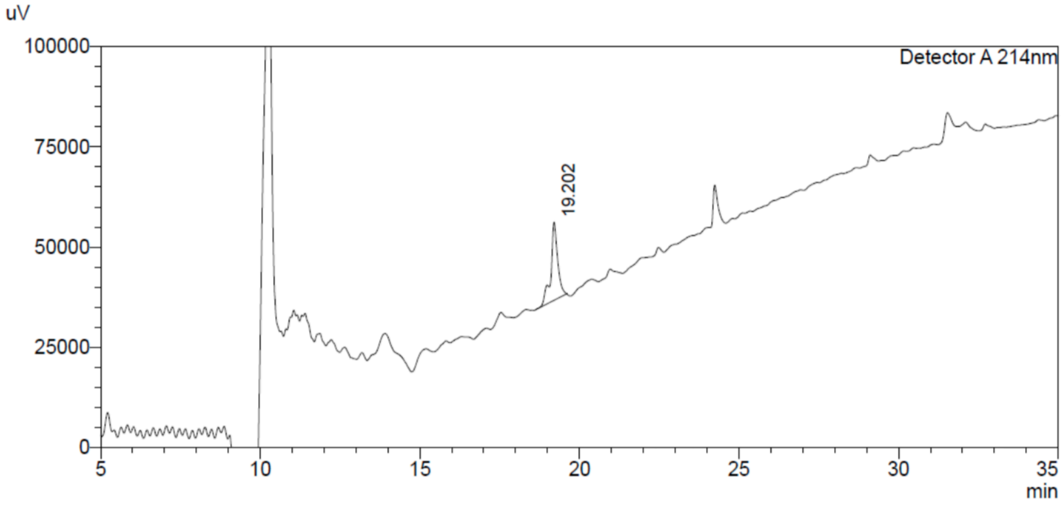

RT = 19.2 min in a gradient of 10-10-70% ACN in 35 minutes

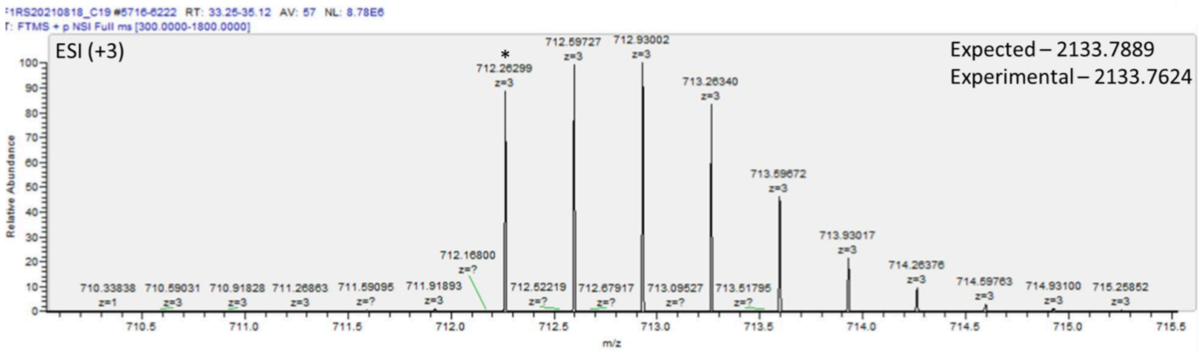

Supplementary Figure 14. Characterisation of fPeptide linked to vancomycin conjugates - continued
Structure, and LC and MS profiles for each of the conjugates (**C1-19)** used in this study.

B1
fMLFK(Bodipy)Pra-NH$_2$

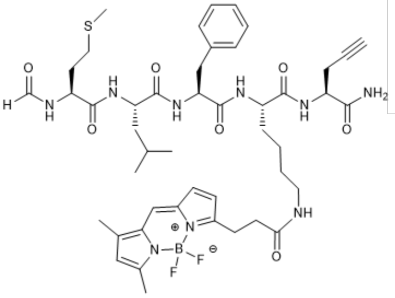

Chemical Formula: $C_{46}H_{62}BF_2N_9O_7S$
Exact Mass: 933.46
Molecular Weight: 933.92

Expected MW – 933.4 Da;
Found m/z – 933.4 Da.

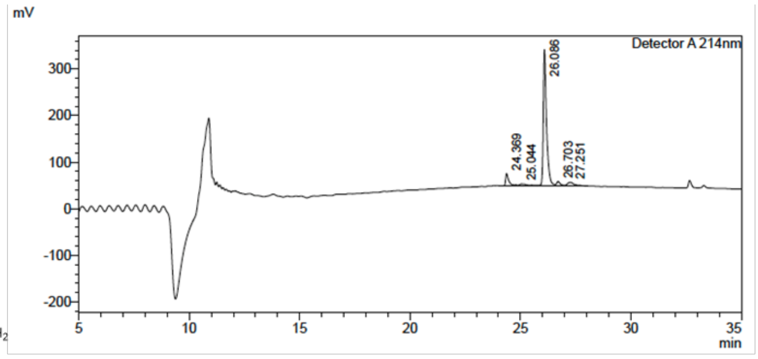

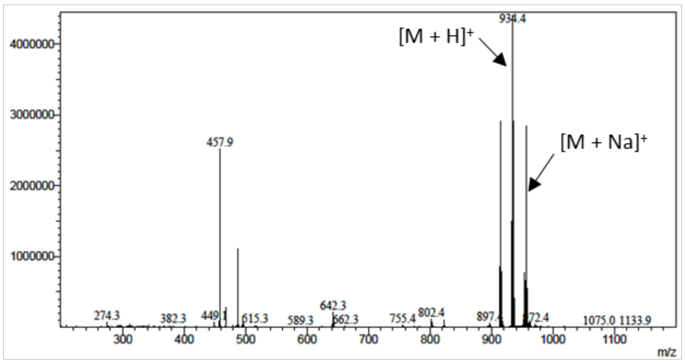

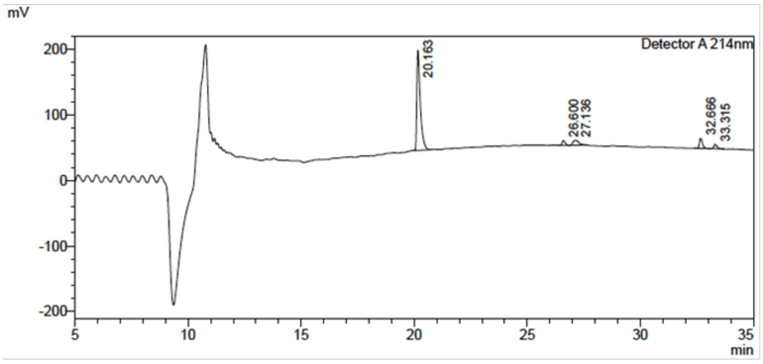

B2
Van-C(Bodipy)

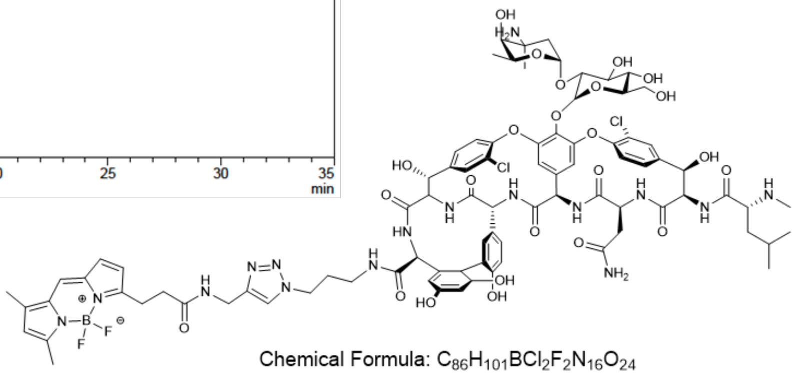

Chemical Formula: $C_{86}H_{101}BCl_2F_2N_{16}O_{24}$
Exact Mass: 1860.66
Molecular Weight: 1862.55

Expected MW – 1860.7 Da;
Found m/z – 1860.2 Da.

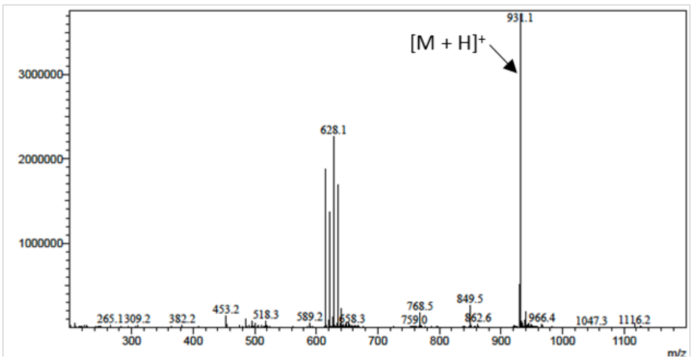

Supplementary Figure 15. Characterisation of BODIPY labeled compounds - continued
Structure, and LC and MS profiles for each of the conjugates (C1-19) used in this study.

B3

Van-C(fMLFG-Tz)-N(BODIPY)

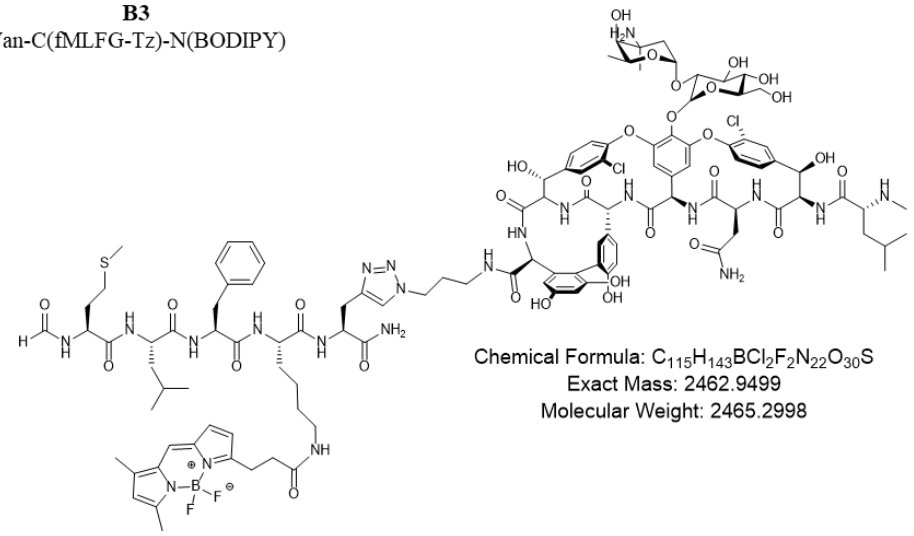

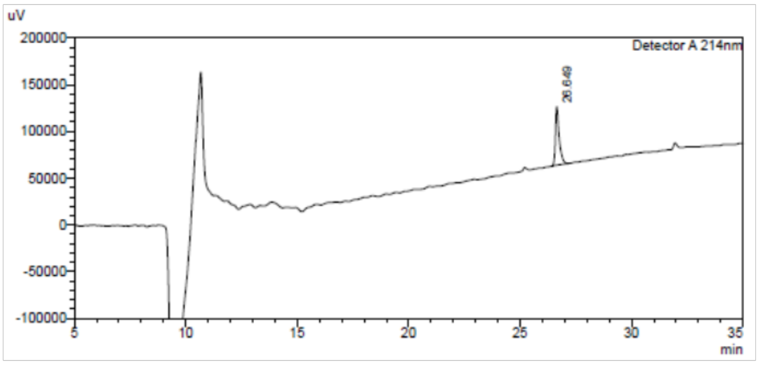

RT = 26.6 min in a gradient of 10-10-70% ACN in 35 minutes

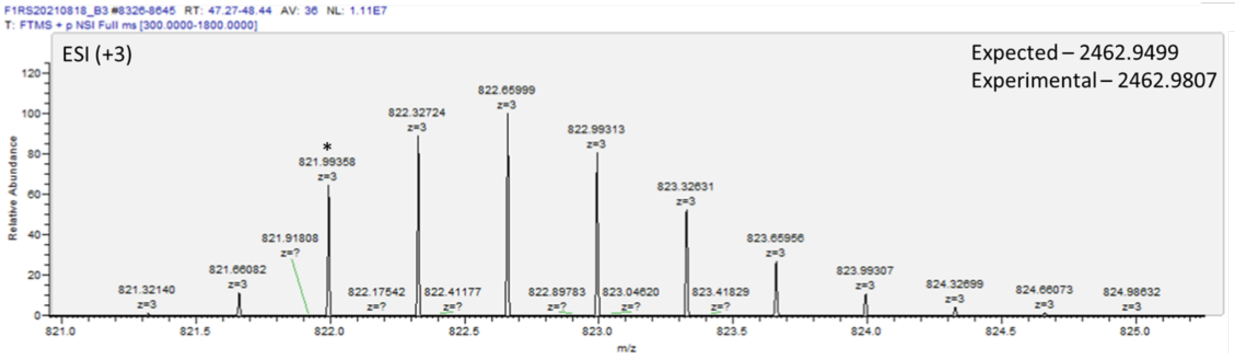

Supplementary Figure 15. Characterisation of BODIPY labeled compounds - continued

Structure, and LC and MS profiles for each of the conjugates (C1-19) used in this study.

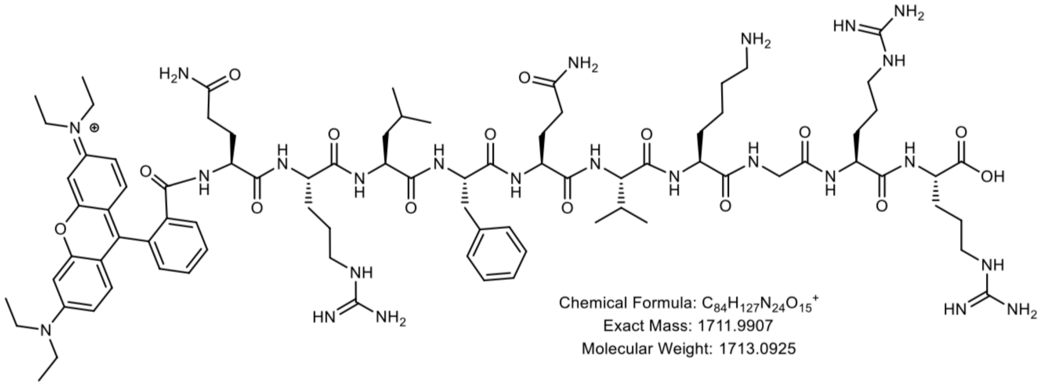

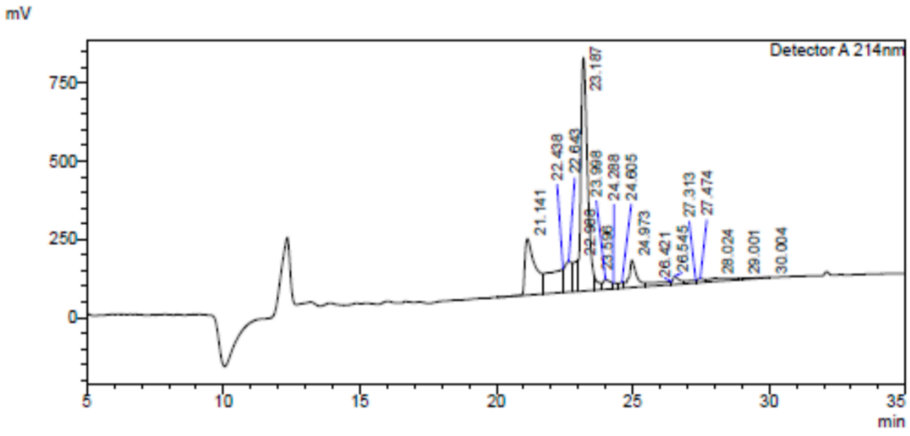

RT = 23.2 min in a gradient of 10-10-100% ACN in 35 minutes

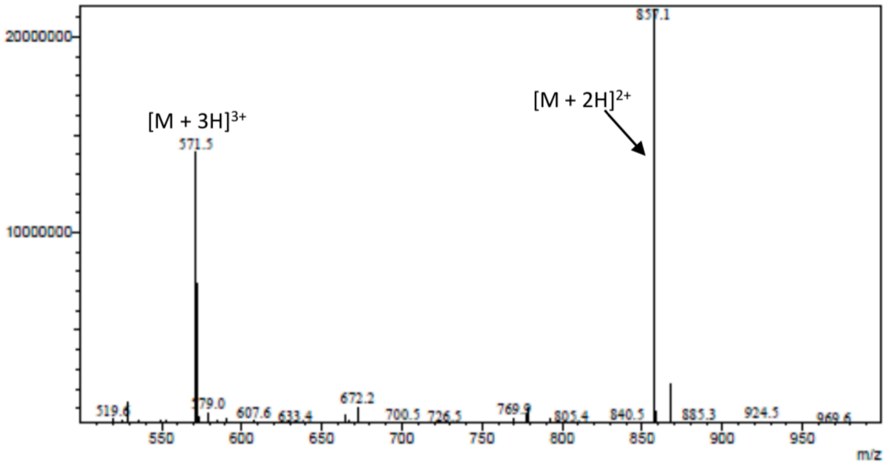

Expected MW – 1711.99 Da;
Found m/z – 1712.2 Da.

Supplementary Figure 16. Characterisation of Rhodamine B-PB10 peptide inhibitor of FRP2

Structure, and LC and MS profiles for Rhodamine B-PB10 peptide inhibitor of FRP2.

SUPPLEMENTARY TABLES

Supplementary Table 1 Antimicrobial activity of vancomycin-fMLFG conjugate against clinical *S. aureus* isolates.

		MIC (µM ±SD, n=4)			
***S. aureus* Strain**	**Antibiotic sensitivity**	**Vancomycin**	**fMLFG-vancomycin**	**Genotype**	**Reference**
ATCC 29213	MSSA	1 ±0.25	5 ±0.4	-	
A5937	MRSA	0.6 ±0.01	4.5 ±0.2	ST5	[4]
A5948	MRSA	0.3 ±0.03	2.4 ±0.2	ST8	[5]
A6224	MRSA	1.2 ±0.02	9.5 ±0.03	ST5	[4]
A6300	MRSA	1 ±0.2	5 ±0.3	ST5	[4]
A8090	MRSA	0.6 ±0.01	4.6 ±0.05	ST5	[6]
A8819	daptomycin susceptible	0.8 ±0.1	4.9 ±0.3	ST105	[5]
A9635	MSSA	0.6 ±0.01	4.4 ±0.3	ST1892	[6]
A9719	MRSA	1 ±0.3	6.2 ±1.2	ST5	[5]
A9754	MRSA	1.1 ±0.06	6.6 ±2.3	ST8	[5]

Vancomycin linked to fMLFG through the C-terminus with 4 PEG-linker (**C10**) was compared to vancomycin alone in regards to growth inhibition of Hospital acquired clinical strains of MRSA, MSSA and the American Type Culture Collection strain (ATCC 29213). A microbroth dilution assay was used to measure the growth of these strains, the minimal inhibitory concentration was determined as the concentration with 90% growth inhibition as compared to the untreated control at 20 h.

Supplementary Table 2 Characterisation of the formylated peptide library (1-27).

	FP No.	Abbreviated name	MW (Da) Theoretical	m/z Exp	LC t_R (min)	LogP	SASA
X_1	1	fMLFG-NH_2	493.6	494.3	18.8	-0.95	464.82
	2	H-MLFG-NH_2	465.6	466.3	10.9	-0.77	447.77
	3	Ac-MLFG-NH_2	507.7	508.3	18.1	-0.83	499.37
X_2	4	f-Met(O)-LFG-NH_2	509.6	510.3	14.6	-3.08	468.87
	5	f-Met(O_2)-LFG-NH_2	525.6	526.3	16.2	-3.03	481.44
	6	f-Nle-LFG-NH_2	475.6	476.3	23.0	0.04	467.01
	7	f-Nva-LFG-NH_2	461.6	462.3	20.1	-0.38	440.73
X_3	8	fM-Nle-FG-NH_2	493.6	494.3	24.4	-0.86	478.56
	9	fM-Nva-FG-NH_2	479.6	480.2	21.0	-1.28	452.51
	10	fM-tLeu-FG-NH_2	493.6	494.3	22.2	-0.76	450.72
	11	fM-Cha-FG-NH_2	533.7	534.3	30.0	-0.19	499.34
X_4	12	fML-Tyr-G-NH_2	509.6	510.3	12.7	-1.34	468.69
	13	fML-Asp-G-NH_2	461.5	462.2	9.7	-3.4	437.44
	14	fML-Glu-G-NH_2	475.6	476.2	9.6	-3.12	459.73
	15	fML-Phe(4-F)-G-NH_2	511.6	510.3	21.4	-0.79	473.36
	16	fML-Phe(4-Cl)-G-NH_2	528.1	528.3	24.5	-0.39	490.28
	17	fML-Phe(4-NH_2)-G-NH_2	508.6	509.3	7.6	-1.75	492.19
	18	fML-Phe(4-CN)-G-NH_2	518.6	519.3	14.8	-0.91	494.26
X_5	19	fMLF-Leu-NH_2	549.7	550.3	24.3	0.78	509.85
	20	fMLF-Arg-NH_2	592.8	593.3	13.8	-0.99	552.59
	21	fMLF-Lys-NH_2	564.8	565.3	13.1	-0.71	543.38
	22	fMLF-Glu-NH_2	565.7	566.2	17.1	-0.95	530.36
	23	fMLF-Gln-NH_2	564.7	565.3	15.7	-1.61	542.65
	24	fMLF-His-NH_2	573.7	574.2	13.3	-1.52	512.19
	25	fMLF-Ser-NH_2	523.7	524.2	16.3	-1.31	498.86
	26	fMLF-Pro-NH_2	533.7	534.3	17.6	-0.24	486.21
	27	fMLF-Phe-NH_2	583.8	584.3	26.5	1.22	529.13

Supplementary Table 3 High resolution mass spectrometry analysis of peptides

No.	Abbreviated name	MW Theoretical (Da)	m/z Experimental (Da)	formula	Δppm
**C1**	Van-C(fMLFG-Tz)	2117.7675	2117.7977	$C_{97}H_{121}Cl_2N_{19}O_{29}S$	14.3
**C11**	Van-C(fM-Cha-FG-Tz)	2157.7988	2157.8277	$C_{100}H_{125}Cl_2N_{19}O_{29}S$	13.4
**C12**	Van-C(H-MLFG-Tz)	2089.7726	2089.8046	$C_{96}H_{121}Cl_2N_{19}O_{28}S$	15.3
**C13**	Van-C(fNle-LFG-Tz)	2099.8111	2099.8420	$C_{98}H_{123}Cl_2N_{19}O_{29}$	14.7
**C14**	Van-C(fM-Nle-FG-Tz)	2117.7675	2117.7983	$C_{97}H_{121}Cl_2N_{19}O_{29}S$	14.5
**C15**	Van-C(fML-Phe(4-F)-G-Tz)	2135.7581	2135.7835	$C_{97}H_{120}Cl_2FN_{19}O_{29}S$	11.9
**C16**	Van-C(fMLF-Pro-Tz)	2157.7988	2157.8263	$C_{100}H_{125}Cl_2N_{19}O_{29}S$	12.7
**C17**	Van-C(fMLF-Lys-Tz)	2188.8410	2188.8667	$C_{101}H_{130}Cl_2N_{20}O_{29}S$	11.7
**C18**	Van-C(fMLF-Leu-Tz)	2173.8301	2173.8579	$C_{101}H_{129}Cl_2N_{19}O_{29}S$	12.8
**C19**	Van-C(fML-Tyr-G-Tz)	2133.7624	2133.7889	$C_{97}H_{121}Cl_2N_{19}O_{30}S$	12.4

SUPPLEMENTARY REFERENCES:

1. Schindelin, J. et al. Fiji: an open-source platform for biological-image analysis. *Nature Methods* **9**, 676-682 (2012).
2. Tailhades, J. et al. A chemoenzymatic approach to the synthesis of glycopeptide antibiotic analogues. *Angewandte Chemie International Edition* **59**, 10899-10903 (2020).
3. Forsman, H. et al. Structural changes of the ligand and of the receptor alters the receptor preference for neutrophil activating peptides starting with a formylmethionyl group. *Biochimica et Biophysica Acta* **1853**, 192-200 (2015).
4. Cameron, D.R. et al. Vancomycin-intermediate *Staphylococcus aureus* isolates are attenuated for virulence when compared with susceptible progenitors. *Clinical Microbiology and Infection* **23**, 767-773 (2017).
5. Peleg, A.Y. et al. Whole genome characterization of the mechanisms of daptomycin resistance in clinical and laboratory derived isolates of *Staphylococcus aureus*. *PLoS One* **7**, e28316 (2012).
6. Peleg, A.Y. et al. Reduced susceptibility to vancomycin influences pathogenicity in *Staphylococcus aureus* infection. *The Journal of Infectious Diseases* **199**, 532-536 (2009).
