# Supplementary material for: Antibiotic-chemoattractants enhance neutrophil clearance of Staphylococcus aureus
Source: Nat Commun. 2021 Oct 25;12:6157. doi: 10.1038/s41467-021-26244-5 (PMC8546149; doi:10.1038/s41467-021-26244-5)
Supplement: Supplementary file 3 — Reporting Summary [file 41467_2021_26244_MOESM3_ESM.pdf]

nature portfolio

Corresponding author(s): Cryle & Payne

Last updated by author(s): 19/8/21

Reporting Summary

Nature Portfolio wishes to improve the reproducibility of the work that we publish. This form provides structure for consistency and transparency in reporting. For further information on Nature Portfolio policies, see our Editorial Policies and the Editorial Policy Checklist.

Statistics

For all statistical analyses, confirm that the following items are present in the figure legend, table legend, main text, or Methods section.

n/a	Confirmed	
☐	☒	The exact sample size (n) for each experimental group/condition, given as a discrete number and unit of measurement
☐	☒	A statement on whether measurements were taken from distinct samples or whether the same sample was measured repeatedly
☐	☒	The statistical test(s) used AND whether they are one- or two-sided<br>*Only common tests should be described solely by name; describe more complex techniques in the Methods section.*
☒	☐	A description of all covariates tested
☐	☒	A description of any assumptions or corrections, such as tests of normality and adjustment for multiple comparisons
☐	☒	A full description of the statistical parameters including central tendency (e.g. means) or other basic estimates (e.g. regression coefficient) AND variation (e.g. standard deviation) or associated estimates of uncertainty (e.g. confidence intervals)
☐	☒	For null hypothesis testing, the test statistic (e.g. F, t, r) with confidence intervals, effect sizes, degrees of freedom and P value noted<br>*Give P values as exact values whenever suitable.*
☒	☐	For Bayesian analysis, information on the choice of priors and Markov chain Monte Carlo settings
☒	☐	For hierarchical and complex designs, identification of the appropriate level for tests and full reporting of outcomes
☒	☐	Estimates of effect sizes (e.g. Cohen's d, Pearson's r), indicating how they were calculated

Our web collection on statistics for biologists contains articles on many of the points above.

Software and code

Policy information about availability of computer code

Data collection	Nikon NIS element v4<br>CLARIOstarv5.20R5<br>LabSolutions v5.97SP1
Data analysis	GraphPad Prism v9<br>FIJI image J with trackmate plugin<br>Chemdraw v19<br>Phenix v1.19.2<br>Chimera v1.15<br>MARS data analysis software v3.10<br>Software has also been mentioned in text where relevant.

For manuscripts utilizing custom algorithms or software that are central to the research but not yet described in published literature, software must be made available to editors and reviewers. We strongly encourage code deposition in a community repository (e.g. GitHub). See the Nature Portfolio guidelines for submitting code & software for further information.

Data

Policy information about availability of data

All manuscripts must include a data availability statement. This statement should provide the following information, where applicable:

- Accession codes, unique identifiers, or web links for publicly available datasets
- A description of any restrictions on data availability
- For clinical datasets or third party data, please ensure that the statement adheres to our policy

Data for all figures has been provided in excel spreadsheet with the paper submission. HRMS data has been deposited online in the public available depository. Microfludic raw microscope images are availble from the authors on request via thumb drive. Desposity upload and download of this data is not feasible as each image set is over 100gb.

Field-specific reporting

Please select the one below that is the best fit for your research. If you are not sure, read the appropriate sections before making your selection.

☒ Life sciences ☐ Behavioural & social sciences ☐ Ecological, evolutionary & environmental sciences

For a reference copy of the document with all sections, see nature.com/documents/nr-reporting-summary-flat.pdf

Life sciences study design

All studies must disclose on these points even when the disclosure is negative.

Sample size	N of 4 was determined as the minimum number required to achieve the minimum group sample size by ANOVA.
Data exclusions	No data was excluded
Replication	Experiments were reproducible with experiments replicated across at least three biological replicates
Randomization	Animals were randomly assigned to groups. For other testing one a biological replicate was tested in all conditions, and therefore not requiring randomization.
Blinding	Investigators were blinded to the identity of groups for data analysis of all microscope image analysis - this included microfludics, BODIPY binding and histology. Blinding for other experiments was performed for data collection, however not possible during the data analysis.

Reporting for specific materials, systems and methods

We require information from authors about some types of materials, experimental systems and methods used in many studies. Here, indicate whether each material, system or method listed is relevant to your study. If you are not sure if a list item applies to your research, read the appropriate section before selecting a response.

Materials & experimental systems

n/a	Involved in the study	
☒	☐	Antibodies
☐	☒	Eukaryotic cell lines
☒	☐	Palaeontology and archaeology
☐	☒	Animals and other organisms
☐	☒	Human research participants
☒	☐	Clinical data
☒	☐	Dual use research of concern

Methods

n/a	Involved in the study	
☒	☐	ChIP-seq
☒	☐	Flow cytometry
☒	☐	MRI-based neuroimaging

Eukaryotic cell lines

Policy information about cell lines

Cell line source(s)	ATCC CHO
Authentication	Not applicable (generated in house)

Mycoplasma contamination	Mycoplasm-free
Commonly misidentified lines (See ICLAC register)	This cell line used is not present on the ICLAC register

Animals and other organisms

Policy information about studies involving animals; ARRIVE guidelines recommended for reporting animal research

Laboratory animals	Eight week old female Balb/c mice. Mice were housed with 12/12h light dark cycle with room temperature between 18 – 24 ?C, and 40 - 70 % humidity.
Wild animals	No wild animals used
Field-collected samples	No field collected saamples
Ethics oversight	All experiments were performed in accordance with the Animal Research Ethics Committee at Monash University (MARP/2018/012).

Note that full information on the approval of the study protocol must also be provided in the manuscript.

Human research participants

Policy information about studies involving human research participants

Population characteristics	For transwell assays: Healthy human donors aged over 18 years of both sexes, who are non-smokers and did not take anti-inflammatories or alcohol in the 48h prior. No genotype information was collected.<br>For microfluidic assays: healthy volunteers, both male and female, aged over 18 years old who were not receiving immunosuppressants
Recruitment	For neutrophils used in microfluidic devices: de-identified fresh blood was purchased from Research Blood Components, where blood samples were obtained from healthy volunteers aged 18 years or over, who were not receiving immunosuppressant agents. Biased to the population of people that is recruited by the Research Blood Components Group. Additionally venous blood samples from healthy volunteers were collected by phlebotomy after receipt of written informed consent.<br><br>For transwell assay, donors were recruited from the University population. Written informed consent from volunteers was obtained and donors were de-identified. As donors were recruited from the university population, this may bot include a true cross section of the population.
Ethics oversight	Monash University Human Research Ethics committee project #9572<br>MGH Institutional Review Board protocol 2008-P-002123

Note that full information on the approval of the study protocol must also be provided in the manuscript.
